# Reflective practice of nurse residents in the teaching-learning process in teaching hospitals

**DOI:** 10.1590/0034-7167-2023-0540

**Published:** 2024-09-06

**Authors:** Ana Carolina de Oliveira Paiva, Kênia Lara Silva

**Affiliations:** I Universidade Federal de Minas Gerais. Belo Horizonte, Minas Gerais, Brazil

**Keywords:** Learning, Nurses, Hospitals, Teaching, Professional Practice, Preceptorship, Aprendizaje, Enfermeras y Enfermeros, Hospitales de Enseñanza, Práctica Profesional, Preceptoría

## Abstract

**Objective:**

To analyze reflective practice in the teaching-learning process of nurses in residency programs in teaching hospitals in Minas Gerais, Brazil.

**Methods:**

Case study, based on the reflective practice framework, conducted in two teaching hospitals. Observation and interviews were conducted with first and second-year residents, and five participants were included for in-depth analysis, with their data subjected to frequency distribution analysis and Critical Discourse Analysis.

**Results:**

In 519 observed activities, elements of reflection were identified in 22.2%, especially active listening and expression of doubts. Discourses indicated practice as the best moment for teaching-learning due to its potential to generate reflections. Learning by doing and case discussion were considered potential strategies for reflective learning.

**Conclusion:**

Know-in-action reflection was evidenced as the predominant formative aspect for residents, with few opportunities for reflection on reflection-in-action.

## RESUMEN

**Objetivo:**

Analizar la práctica reflexiva en el proceso de enseñanza-aprendizaje de enfermeras en programas de residencia en hospitales de enseñanza en Minas Gerais, Brasil.

**Métodos:**

Estudio de caso, basado en el marco de la práctica reflexiva, realizado en dos hospitales de enseñanza. Se realizaron observaciones y entrevistas con residentes de primero y segundo año, y se incluyeron cinco participantes para un análisis en profundidad, cuyos datos fueron sometidos a análisis de distribución de frecuencia y Análisis Crítico del Discurso.

**Resultados:**

En 519 actividades observadas, se identificaron elementos de reflexión en un 22.2%, especialmente la escucha activa y la expresión de dudas. Los discursos indicaron que la práctica es el mejor momento para el aprendizaje debido a su potencial para generar reflexiones. Aprender haciendo y la discusión de casos fueron consideradas estrategias potenciales para el aprendizaje reflexivo.

**Conclusion:**

Se evidenció que la reflexión sobre la acción es el aspecto formativo predominante para los residentes, con pocas oportunidades para reflexionar sobre la reflexión en la acción.

## INTRODUCTION

Professional Health Area Residency, established by Law No. 11,129 of 2005, is characterized as service education aimed at healthcare professionals, with the exception of the medical category^(1)^. It plays a pivotal role in specialist training, aligned with the principles and guidelines of the Brazilian Unified Health System (SUS in Portuguese), and proposes the integration of theory and practice to foster critical reflection among residents regarding their experienced reality^(2, 3)^.

The implementation of Professional Health Area Residency Programs (PHARPs) in Brazil dates back over 10 years. However, studies indicate challenges related to the residents’ training process that still need to be overcome. Among these challenges are the traditional teaching model, the centrality of medical knowledge, the lack of interaction among healthcare professionals, fragmented and disconnected practices from the patient’s social context, and the inadequacy of professionals for preceptorship and critical, reflective teaching on professional practice experiences^(2, 4, 5)^.

In this sense, evidence suggests that resident training is oriented towards a biologistic model, where the patient’s view is reductionist and fragmented, emphasizing technical skills over relational ones, thus placing the student in a passive role^(2, 4, 5)^. Consequently, reflective practice in the teaching-learning process of PHARPs is weakened, focusing on procedures, technique repetition, and knowledge transmission, with limited theoretical discussion and little interaction between preceptor-tutor and service-based teaching. Additionally, the presence of preceptors without pedagogical training who juggle multiple roles and sometimes do not identify with the role of healthcare educators is noted. This creates a cycle of task transfer to residents, impacting their learning.

In this context, the question arises: How does reflective practice occur in the teaching-learning process of nurses in residency programs? What elements of reflective practice are revealed in this process? Investigating reflective practice in the teaching-learning process of nurses in PHARPs is justified by the relevance of this formative process in shaping critical and creative professionals who reflect on and about their actions. There is recognition of the role of residencies as a defense and training strategy for SUS. Therefore, the study results can strengthen this teaching model and contribute to the reformulation of PHARP pedagogical projects.

The current research is grounded in Donald Schon’s theoretical framework concerning reflective practice within the teaching-learning process^(6)^. According to Schon, professional training still leans towards technical and scientific prowess, centered on the Cartesian model, where theory is detached from practice, with the latter viewed as a byproduct of theoretical education. This approach fails to cultivate creative professionals capable of meeting workforce demands^(6)^.

From this standpoint, an alignment between theory and practice is advocated for professional training in reflective teaching. This approach encourages reflective capacity through teacher-student interaction across various practical scenarios, emphasizing learning by doing. The framework of reflective practice posits that effective professional performance arises solely from reflecting on uncertain and conflicting situations encountered in daily practice^(6)^.

It’s noteworthy that literature on reflective practice, as proposed by Donald Schön, within the teaching-learning process of nursing residency programs, remains scant nationally^(2, 3, 7, 8, 9, 10, 11, 12, 13)^. Studies primarily focus on preceptor training, residents’ and preceptors’ perceptions of the teaching-learning process in PHARPs, alongside interprofessional and continuing education within PHARPs. Hence, there persists a necessity to scrutinize the teaching-learning process in PHARPs, underpinned by a theoretical framework that bolsters the residency’s intrinsic principles, particularly reflective practical experience.

## OBJECTIVE

To analyze reflective practice in the teaching-learning process of nurses in residency programs at teaching hospitals in Minas Gerais, Brazil.

## METHODS

### Ethical aspects

This research was approved in 2021 by the Research Ethics Committee of the Federal University of Minas Gerais, in compliance with the ethical principles of Resolution 466/2012. Participants signed an Informed Consent Form and were coded to ensure confidentiality and anonymity.

### Study design

This is a qualitative study, specifically a single-case study. Reflective practice in the teaching-learning process of nurses in residency programs was defined as the case. The criteria proposed by the Consolidated Criteria for Reporting Qualitative Research (COREQ)^(12)^ were adopted.

### Methodological procedures

This study is part of a doctoral thesis conducted in three phases: document analysis, peripheral participant observation, and semi-structured interviews^(13)^, forming a data triangulation approach for the case study. This article presents data from observation and interviews.

### Study setting

The context of the case where reflective practice in the teaching-learning process occurs was the uniprofessional and multiprofessional PHARPs. The units of analysis were two teaching hospitals located in Minas Gerais, Brazil. One hospital, referred to as Unit of Analysis 1, is a general hospital specializing in trauma care, offering multiprofessional PHARPs in elderly health and uniprofessional PHARPs in Intensive Care, Emergency, and Trauma; the other, referred to as Unit of Analysis 2, specializes in women’s health with a uniprofessional PHARP in obstetric nursing.

### Data source

The study population consisted of 47 nursing residents, out of the 53 enrolled in PHARPs at the scenario hospitals, after applying exclusion criteria. Three residents from the obstetric nursing PHARP were on medical leave during the data collection period; one first-year resident of the elderly health PHARP contributed to the pilot test; and two second-year residents of the elderly health PHARP withdrew from the specialization at the time of data collection.

For participant selection, a non-probabilistic method of proportional stratified random sampling was used to enhance sample representativeness^(13)^. The population was divided into homogeneous groups: one stratum for first-year residents and another for second-year residents, considering that reflective practice progresses during professional training. Data collection was halted upon thematic saturation, confirmed by the identification of redundancy and recurrence of information. This saturation model observes the recurrence of codes or themes identified, as exemplified in the data^(14)^. This process was confirmed through the reading and pre-analysis of each collected material regarding the participants individually, after the inclusion of three first-year residents and two second-year residents.

Chart 1 presents the study population and the distribution of residents according to the units of analysis.

**Chart 1:** Study population and distribution of observed nursing residents by unit of analysis, Belo Horizonte, Minas Gerais, Brazil, 2023

Unit of analysis	PHARP	Residency year	Number of residents enrolled in PHARP	Selected resident code
1	Multiprofessional Elderly Health	1st year	2	R1
		2nd year	2	-
	Uniprofessional Intensive Care, Emergency, and Trauma	1st year	2	R4
		2nd year	1	R2
2	Uniprofessional Obstetric Nursing	1st year	24	R3
		2nd year	22	R5

*Note: PHARP - Professional Health Area Residency Programs.*

### Data collection and organization

Data collection occurred from August 2022 to July 2023. Prior to this, a pilot study was conducted. A 2-hour observation was followed by a 10-minute and 36-second interview with a first-year resident of the Elderly Health PHARP. At the observation’s conclusion, the applicability of the proposed observation script was confirmed. Regarding the interview, adjustments were made to ensure the resident’s comprehension of the questions. After revision, the questions underwent testing in a new pilot interview, where their applicability was once again confirmed. Data from the pilot study were not included in the final research.

During observation, the focus was on reflective practice from the resident’s perspective. Residents were individually observed to identify teaching-learning scenarios. Upon identification, notes were recorded in a researcher’s digital diary, including: (1) a description of the observed activity; (2) elements of reflective practice expressed by the resident; (3) actors who enhanced this reflection; and (4) moments when these teaching-learning scenarios occurred, whether during a practical activity, theory, case study, or tutoring.

It is crucial to emphasize that students demonstrate reflection through their performances (6). Thus, while observing residents, the researcher identified some indicators of reflective performance in situations where the student: (1) was surprised by something; (2) paused during the execution of an activity, accompanied by silence; (3) commented or criticized something about the activity; (4) listened with focused attention; and (5) expressed doubts. These indicators were termed as elements of reflective practice in the research.

This stage resulted in the observation of five residents for a total of 156 hours and 10 minutes. Observations took place on weekdays and weekends, during morning and afternoon shifts. The average daily observation time was 6 hours, with each resident observed for approximately 31 hours.

After completing all observations of each resident, interviews with the respective students followed. The interviews were guided by a semi-structured script containing brief descriptions of the teaching-learning scenarios observed by the researcher during data collection. Based on these descriptions, residents were prompted to reflect on their perceptions of the scene, as well as on factors that facilitated or hindered reflexivity in that situation and in other teaching-learning moments. The interviews with the five observed residents were audio-recorded, with an average duration of 19 minutes and 25 seconds, totaling 1 hour, 37 minutes, and 5 seconds.

### Data analysis

To ensure anonymity, each resident was assigned a code (R1, R2, R3, R4, and R5), which remained consistent across both observation and interview phases. Observation data were organized in an Excel spreadsheet, where the activities performed by the residents and observed by the researcher were described. Each activity was named and analyzed to identify elements of reflective practice expressed by the resident, actors that enhanced this reflection, and the stage of the teaching-learning process in which the activity occurred. Upon completing the description and analysis of activities performed by the five residents during the 156 hours and 10 minutes of observation, the researcher proceeded to compile and analyze the data using absolute and relative frequency distribution.

Interviews were transcribed using the InqScribe software, in its free version. To indicate speaker overlaps, pauses, silences, intonation, interruptions, and incomprehensible passages in the text, transcription conventions by Marcuschi^(15)^ were adopted. These conventions not only express speech but also non-verbal elements capable of demonstrating reflection, such as pauses, silences, gestures typical of surprise, and attention.

Critical Discourse Analysis was conducted from Fairclough’s perspective^(16)^ to analyze empirical data based on the manifest content in the discourses, correlating them with the theoretical framework. Operationally, an analytical matrix was developed to extract data from each interview, which was meticulously examined, identifying discursive elements line by line, paragraph by paragraph. Following this step, vertical reading of each axis and transversal reading of the material were conducted, aiming to understand in-depth the corpus of analysis that would allow the reconstruction of knowledge about the phenomenon in question.

## RESULTS

The residents participating in the study had an average undergraduate training period of 2.6 years and were all in their first specialization course. Only two of them had previous professional experience before joining the PHARPs.

During 156 hours and 10 minutes of observation, 519 activities performed by the residents throughout their respective PHARPs were analyzed. These activities were categorized into four teaching-learning moments: practical activity, theoretical activity, case study, and tutoring (Table 1).

**Table 1:** Number of activities with reflective elements, elements of reflective practice identified, and actors, according to the teaching-learning moments, Belo Horizonte, Minas Gerais, Brazil, 2023

Teaching-learning moments	Observation time	Quantitative of observed activities	Quantitative of activities with elements of reflective practice (n.%)	Quantitative of elements of reflective practice	Quantitative of actors who potentiated reflective practice
Practical Activity	137 hours and 10 minutes	500	101 (20.2)	163	143
Theoretical Activity	4 hours	4	3 (75)	4	4
Case Study	6 hours	8	7 (87.5)	9	9
Tutoring	9 hours	7	4 (57.1)	7	7
Total	156 hours and 10 minutes	519	115 (22.2)	183	163

519 activities were categorized into 30 types, as shown in Table 2, highlighting the number of activities in which at least one element of reflective practice was identified. Additionally, Table 2 presents the 13 categories of elements of reflective practice observed in the activities performed by the residents: a. attentive listening (43); b. expressing doubts (34); c. commenting or criticizing something about the activity (19); d. association (18); e. exchanging ideas/discussion (18); f. pausing during activity execution, accompanied by silence (9); g. detecting the need to change or do something different during or after the action (8); h. explaining the activity itself (7); i. insight (7); j. investigation (6); k. evaluating what was done (5); l. being surprised by something (5); m. comparison (4).

**Table 2:** Number of activities with identification of elements of reflective practice and categories of elements of reflective practice, Belo Horizonte, Minas Gerais, Brazil, 2023

Activities (n)	Activities with elements of reflective practice (n.%)	Quantitative of elements of reflective practice	Reflective practice elements identified in activities in descending order
Patient assessment (91)	14 (15.4)	23	b, d, a, f, j, e, k, c, l
Nursing prescription or documentation (73)	7 (9.6)	11	b, j, e, m, g, c, a
Patient care (57)	10 (17.5)	17	m, h, b, f, i, k, a, e
Orientation (52)	-	-	-
Transfer of care (36)	3 (8.3)	3	b, a
Clinical case discussion (33)	14 (42.4)	18	a, c, b, e, g, i, j, l
Work process management (31)	2 (6.5)	2	c, b
Execution of technique or procedure (23)	8 (34.8)	12	g, a, h, f, c, j
Nursing team management (23)	3 (13)	5	a, d, k, g
Observation of professional performing activity (21)	14 (66.7)	25	a, b, e, d, g, c, h, i, l
Analysis of exams (19)	8 (42.1)	14	b, c, e, d, a, l
Bed management (11)	4 (36.4)	7	b, i, c, a, f,
Clarification of doubts (9)	6 (66.7)	13	b, a, e, k, c
Cleaning and disinfection (6)	-	-	-
Receiving guidance (5)	2 (40)	2	a, l
Case study presentation (3)	2 (66.7)	3	d, c
Case study discussion (3)	3 (100)	4	d, a, h
Preparation of TCR (3)	3 (100)	7	c, d, k, g, a, b
Feedback (3)	1 (33.3)	1	i
Attending presentation of work (2)	2 (100)	3	e, d, c
Attending case study presentation (2)	2 (100)	2	a
Lecture(2)	1 (50)	2	d, c
Dynamic (2)	1 (50)	1	d
Preparation of case study (2)	2 (100)	3	e, d
Expressing residency experiences (2)	-	-	-
Conversation circle (2)	2 (100)	4	c, d, a
Work presentation (1)	1 (100)	1	d
Collegiate meeting (1)	-	-	-
PHARP meeting (1)	-	-	-

*Notes: TCR - Residency Conclusion Paper; PHARP - Professional Health Area Residency Programs.*

Through Table 2, seven activities are highlighted in which residents, in 100% of the scenes, expressed at least one reflective element. Of the seven identified, two were categorized as case study teaching-learning moments (participation in presentations and discussion of case studies); two as theoretical activities (giving and attending presentations of works); and one as tutoring (group discussion). The remaining two activities were classified as practical activities, as they occurred within the designated time frame for such activities. However, it is important to note that the elaboration of the Residency Conclusion Work (TCR) and case study are activities associated with tutoring and case study moments, respectively. Therefore, none of the seven identified activities is directly related to practical activity.

Attentive listening was the most commonly expressed reflective element by residents, followed by the elements “expressing doubts” and “commenting or criticizing something about the activity.” Evaluation of what was done, being surprised by something, and comparison were the least expressed elements by residents.

Residents themselves, through self-reflection, were the ones who most enhanced reflection (42.9%), followed by preceptors (25.8%) and nursing resident colleagues (9.2%). Tutors (1.2%), patients (1.2%), and teachers (0.6%) were the actors who least enhanced residents’ reflection.

The activity with the highest observation frequency was patient assessment, with 91 observed scenes, of which, in 14 (15.4%), there was identification of nine types of reflective practice elements and six actors stimulating reflection. The element “expressing doubts” stood out, repeated on seven occasions, and residents engaged in “self-reflection” in nine situations.

The activity of nursing prescription or records was the second most observed, with 73 scenes, of which, in seven (9.6%), there was detection of seven types of reflection elements, with three actors enhancing reflection. The relationship between the highest frequency of activity execution and the low number of reflection element identification is consistent with R1’s discourse. In an interview, the participant characterized this process as “tiring” and “repetitive.” Using the argumentative operator “but,” R1 established an adversative relationship about the nursing record activity, stating that, in their journey as a resident, they initially enjoyed evolving patients, but now consider the activity repetitive:

[...] *evolving is something I used to really enjoy doing before. But nowadays, it’s, it’s becoming tiresome for me, you know? Evolving. Because it’s very repetitive. That repetitive stuff.* (R1)

This discourse differs from R2’s statement, who indicated nursing recording as the activity that most stimulates reflection. The resident perceived the activity as an opportunity to read the progress notes of the multidisciplinary team and understand the patient’s medical history.

*I think it’s when I’m at, at the computer to UPDATE, and then, I always try to see what other professionals’ approach is to that patient too. So, I have my care. And then, I try to see, yeah, to really understand, like, the whole context of the patient, right? So, I think that, like, when I’m really* (+). *I’m going to... I assess my patient and I’m going to sit down to update, I try to understand his whole story, right? So, I think it’s more when I’m in front of the computer.* (R2)

Furthermore, the residents mentioned learning by doing as a teaching and learning strategy:

[...] *the teaching-learning technique is like this: “hum, I’ve never done this before. I’m going to do it”. So, I get my hands dirty and do it, you know?* (R1)

*And then, I see a lot that it’s like that. Sometimes, we’re there in assistance, we learn a lot from that, that doing. Solving that problem, right? And not always understanding what’s behind it. But we do learn, yes.* (R2)

In the first excerpt, the teaching and learning strategy “learning by doing” was considered commonplace. The argumentative operator “but”, used by R2 in the last excerpt, illustrates that even without fully understanding “this doing,” he was still able to learn. Furthermore, R2 highlighted the discussion of clinical cases as a potential strategy for teaching and learning. This assertion is supported by the discursive elements “strategy (that) might be better, that would add more, fix the knowledge better.” Thus, the need for preceptor involvement in discussions is reaffirmed, aiming to take a more active role in the process.

*And then, I think* (+) *a strategy* [that] *might be better, that would add more, would better solidify the knowledge, would be discussions in, um, within the department itself, you know? It’s, involving, um, actual discussion ABOUT the patient’s clinical situation, um, from both the resident’s perspective and the preceptor’s perspective, even.* (R2)

R1 also emphasized the importance of case discussions beyond the moments of residents’ doubts, stressing the need to “deepen” and “better understand his (patient’s) case”.

*Sometimes, I miss more of us sitting down and preceptor and, and resident, and discussing the cases more, you know? Not just, not just when we need to* [...]. [...] *sometimes there are patients that we can’t delve into, like, understand their case better, you know?* [...] *I miss this discussion, more like, working more on this issue of nursing diagnoses, interventions, not being kinda just that, the repetitive situation, you know?* (R1)

In the interviews with R3 and R4, the expression of some reflection elements, such as expressing doubts, is noticeable.

*I always go to a preceptor to clear up my doubts, always.* (R3)

*Usually, I feel quite comfortable, um, for example, if I have any doubts, to ask.* (R4)

In this context, the term “doubt” was mentioned 11 times in R2’s discourse, correlated with the act of doing and operating as an ideological rationalization to justify the set of relationships; that is, from doubt, R2 refrains from acting and then seeks guidance from the preceptor.

*I remember exactly that my doubt, actually, was whether I could try washing or if I had to aspirate. So I preferred not to do it. But that’s it. I think in... Generally, we find ourselves in situations, like, of doubt really... And then, we need the preceptor’s help.* (R2)

In terms of actors, the residents themselves engaging in self-reflection (38%) were the ones who most enhanced reflective practice during the practical activity. Next were the preceptors (25.8%) and other nurses (8%) present in the residents’ daily routine. In the excerpts below, two actors are identified: the resident themselves and the preceptor. The resident engaging in self-reflection expresses: “I’m having difficulty” and “I thought passing the catheter would be super easy. When I got there, the woman was still on a stretcher, so I asked for help from Preceptor Y” moments where R1 analyzes the situation and reflects on their own difficulties.

*Then, I have difficulty, I call Preceptor Y, “hey Preceptor Y, I’m having difficulty”. Then she guides me, you know?* [...] *when I was going to insert the catheter in her* [patient], *I thought she* [the patient] *was much younger than me, actually, so I thought it would be super easy to insert the* [catheter]. *I got there, the woman was still on a stretcher, a stretcher, that’s it, so I asked Preceptor Y for help, then Preceptor Y came over to... she went to the patient, it seems. She likes to teach.* [...] *It’s, executing the technique, that I learned.* (R1)

The role of the preceptor in stimulating reflection is evidenced when the resident explains: “So she (the preceptor) guides me” and “then Preceptor Y came over... she went to the patient, it seems. She likes to teach... Yeah, by performing the technique, I learned”.

In R1’s discourse, doubt and the attempt to do are pointed out as triggering and mobilizing elements of learning. Through doubt, the participant presents the opportunity to learn on their own initiative, rather than being directed by the preceptor. However, even though the preceptor is one of the most present actors, R2’s discourse highlights the passive position of the preceptor at certain moments of the teaching-learning process:

*But, well, most of the teaching-learning situations, I see that it’s me going after the preceptor, you know?* (R2)

The observation of residents during theoretical teaching-learning activities lasted for four hours. During this time, three reflection elements (association; commenting or criticizing something about the activity; and exchanging ideas and discussion) were identified, with two actors (self-reflection and nursing resident colleague) enhancing this reflection.

During the case study moment, residents were observed for six hours. Within this period, four reflection elements (association; attentive listening; commenting or criticizing something about the activity; and explaining the activity) were identified, with three actors (nursing resident colleague, the residents themselves in self-reflection, and a nurse) enhancing this reflection.

Finally, the observation of residents during tutoring lasted for nine hours. Out of the five activities observed, three contained three reflection elements (association; commenting or criticizing something about the activity; and attentive listening), with four actors (the participants themselves in self-reflection, tutor, nursing resident colleague, and teacher) enhancing this reflection.

Regarding these teaching-learning moments, the participants expressed:

*We lack this theoretical part of learning in residency.* [...] *But I think that, also, directing contents of specific axis, [...]. Oh, I, I think if they provided us, ah, contents, indeed, of theoretical deepening, it would be, um, interesting. Thus (+), it would help a lot in this process of integrating, right? Practice and theory.* (R2)

*I think the case studies, even. Taking a case and discussing the, the possible complications of it... Or possible positive outcomes. What exams we should pay more attention to, how to interpret. I think those things that will, will really stimulate, um, our reasoning.* (R3)

*Usually, the theoretical part we’re not having much of* [...] *Now we’ll only have the tutoring, sort of. Just lessons, but it’s not, um, not very focused on the practical part of nursing. We’re seeing more in a context, um, a general context. So, we’re seeing more about care management, like that. It’s not anything, like, directed TOWARDS the practical part of nursing. So, as we don’t have much of this practical part, I kind of try to make up for it at home, um, like, trying to study on weekends.* (R4)

## DISCUSSION

A solid and high-quality educational foundation, coupled with a variety of experiences in clinical practice, facilitate both the acquisition of knowledge and skills in a safer and faster manner and the progression of the professional to proficiency^(17)^. Thus, it is important to emphasize that theoretical teaching activities are essential, but it is practical teaching activities that allow the application of this theory and the development of skills, teamwork, leadership, and self-confidence. Practical situations expose the student to experiencing different experiences necessary for reflection-in-action in situations of uncertainty, singularity, and conflict.

The research results revealed, through the residents’ observations, that activities in which 100% of the reflection elements were identified, in absolute numbers, were underdeveloped by the residents, as they are related to the theoretical educational strategies of the PHARP. The low provision of these types of activities is in accordance with regulations that define 20% of the total PHARP workload for theoretical activities and 80% in the form of practical and theoretical-practical educational strategies^(18)^.

It was found that the activities most performed by residents in the daily routine of PHARP were related to the moment of practical activity teaching-learning. In other words, activities such as patient assessment, nursing care documentation, and patient care are the most frequent in the learners’ daily lives, but they show a low percentage of reflection element identification.

By frequently repeating the execution of a certain task, the student develops tacit action, that is, knowing-in-action. This means that the learner may, at some point, discover that they have internalized the skilled performance^(6)^. Thus, the activity that started as an imitative construction now allows the student to experience something of their own, a new knowledge of their own repertoire, available for use^(6, 10)^.

In view of this, it is inferred that, in the daily lives of the observed residents, reflection of the knowing-in-action type prevailed, a spontaneous and automatic act in which the learner performs easy sequences of activities without having to think about it. It is a tacit process that occurs spontaneously, without conscious deliberation, and that works by providing the intended results as long as the situation remains within the limits of what is learned to be treated as normal^(3, 6, 9)^. This result aligns with a Canadian study, which states that the student uses technical rationality to solve linear situations, in which the same solutions can work in different cases. However, it highlights that, in the face of uncertainty, uniqueness, and not fully known, such as the experiences of nurses in the Covid-19 pandemic, technical rationality is not always sufficient to resolve the situation. On these occasions, reflective practice is necessary to achieve attempts at resolution^(19)^.

In these circumstances, when the threshold of normality is exceeded, residents no longer act automatically and encounter unexpected situations, referred to as surprises, which can challenge the established categories of knowledge-in-action^(6)^. Thus, the occurrence of reflective practice in the daily teaching-learning processes in PHARP is identified.

In this context, residents are observed engaging in activities such as observing professionals perform tasks, clarifying doubts, and discussing clinical cases. That is, when residents can respond to unusual situations in professional practice, they mobilize not only tacit knowledge but also insights derived from observing other performances^(6, 7)^. On these occasions, the overcoming of knowing-in-action occurs, representing the most elementary level of reflection, and knowledge is demonstrated through intelligent actions, which are publicly observable physical performances.

This research captured the skilled performances of residents in attentive listening, expressing doubts, and exchanging ideas with other professionals. This finding is supported by Schön^(6)^, who suggests that during a student’s attempts to produce a design, the instructor’s instructions and receptiveness have significant potential for effectiveness, as the student listens with operative attention, ready to translate instructions into actions.

The occurrence of reflective practice and the subsequent expression of reflective elements lead to a new understanding of professional experiences, which, in turn, leads to the integration of new learning from practice and stimulates personal and professional growth. Learners who undergo this cyclical process become more reflective professionals capable of proposing new design production performances^(19)^.

Considering the above, it’s clear why the activity “observing professionals perform tasks” is one of the most stimulating for residents to express reflective elements, with attentive listening being the most common. In these interactions between students and instructors, reflective practical teaching is established, where residents not only observe skilled performances but also have the opportunity to engage in a mutually reflective dialogue with professionals. This reciprocal exchange between instructors and students tends to encourage a series of design projects, motivating students to enhance their ability to produce competent designs^(6)^.

In these scenarios, residents themselves emerge as the primary drivers of their reflections, followed by preceptors. Therefore, it’s suggested that self-reflection enhances reflective practice in the daily life of residency, particularly in situations where residents actively participate in the teaching-learning process. The interview findings corroborated this, with participants adopting critical attitudes in their speeches, identifying moments of deeper reflection, and indicating opportunities to further develop this process, towards a more proactive approach to learning.

However, it is evident that the reflection elements expressed by residents focused on reflection-in-action and reflection on action, with few opportunities for reflection on reflection-in-action. The latter refers to the act of professionals reflecting retrospectively on past reflection-in-action, which will consolidate understanding of the situation and thus provide the establishment of new strategies^(6, 7, 9)^. The relevance of nurses developing critical-reflexive thinking throughout their training is emphasized, as practical reality demands the ability to make assertive decisions based on scientific evidence and critical analyses of clinical and social contexts. Additionally, it requires the capacity to promote interprofessional questioning and discussions to ensure the quality of care^(11)^.

An integrative review study contributes to this analysis by stating that teaching strategies used by PHARP need to contribute to making the resident an active participant in their own learning process^(20)^. Learning in service is necessary to stimulate the creativity and independence of the student, as well as provide experiences that lead them to problematize and propose intervention alternatives consistent with the scenarios they experience^(8, 20)^.

It is reiterated that PHARP proposes the qualified training of healthcare professionals for the SUS, equipped to transform daily practices and understand the needs of public health. For this purpose, it constitutes a formative strategy that operates against the biomedical model and the uncritical reproduction of learners still coming from educational institutions^(21)^.

In this context, the challenges faced by nurse preceptors in integrating supervision activities and student guidance into their routine are discussed, stimulating a more active stance that promotes the student’s investigative, critical, and reflective attitudes. Studies point out the accumulation of care, teaching, and management work, which reduces preceptorship to the transfer of work routines and care activities, hampering the adequate training process of residents^(2, 22, 23)^. The relevance of preceptor pedagogical training is emphasized to implement a teaching-learning process focused on critical-reflexive thinking and action^(2, 20)^.

In the residents’ speeches, unusual situations stand out as the moments that most stimulate reflection and, consequently, learning, with the preceptor being the first reference in the face of doubt. Unusual situations reflect the repertoire of knowledge; that is, with each new experience of the learner, the professional enriches their repertoire of knowledge and experiences^(6, 10)^. Thus, unfamiliar situations become familiar because the resident has accumulated a repertoire that enables them to deal with different situations. In other words, each new experience of reflection-inaction enriches the repertoire^(6, 22)^.

Furthermore, another circumstance that arises during practical activities and contributes to the knowledge repertoire is the practice of learning by doing. Learning by doing corresponds to what is known as active experimentation, wherein the investigator in reflection-in-action confirms or refutes an action or hypothesis, thereby promoting the resident’s autonomy^(6, 9)^. In their speeches, the interviewees not only regard this practice as a natural phenomenon, through explicit evaluative statements, but also declared that they “learn a lot.”

Doubt and the attempt to act are also triggering and mobilizing elements of learning. Professional artistic talent is defined as the ability of the professional to handle the undetermined zones of practice. Responding to surprises by reformulating strategies, “he behaves more like a researcher trying to model a specialized system than as a ’specialist’ whose behavior is modeled”^(6)^. Through practical and reflective teaching, students acquire types of artistic talent essential for competence in undetermined zones of practice^(6)^.

An integrative review, conducted in the United States, listed the most frequent activities found in the literature that stimulate reflective practice in undergraduate nursing courses. The first mentioned was reflective writing, wherein students construct critical reflective journals to evaluate the course, reflect on issues during clinical placements, or identify strengths and training needs. Additionally, the study highlighted reflective discussions as the second most potent activity for reflection. According to the researchers, discussions reduce learners’ stress and anxiety^(24)^.

It is understood that the daily life of residency can be permeated by various situations of doubt and uncertainty, which should be viewed as opportunities for reflection and learning. Therefore, it is important to provide space for residents to express doubts and share experiences with preceptors and tutors.

### Study limitations

Limitations of the study may be linked to the researcher’s ability to capture reflective elements expressed by residents. While attention was devoted to all events during data collection, it is recognized that this limitation is inherent to the observation method. It is worth noting that, thanks to the adherence and collaboration of all study participants, the researcher was present in all teaching-learning moments. Moreover, it can be stated that this investigation sought rigor through triangulation, extended fieldwork, and the logic of replication.

### Contributions to nursing, health, or public policy

The research contributes to advancing knowledge in the field of health residency training, while also indicating adaptations in the organization and articulation of the teaching-learning process. PHARP is considered an important training strategy for the SUS.

## FINAL CONSIDERATIONS

In the daily life of PHARP, the most performed activities by residents were those in which they expressed fewer elements of reflective practice. Thus, reflection of the knowing-in-action type predominated in the nurse training process in residency programs, especially during practical activities. This reflection was followed by reflections of the reflection-in-action and reflection on action types, with few opportunities for learners to extend reflection to reflection on reflection-in-action. The analysis identified “listening with operative attention” and “expressing doubts” as the most expressed reflection elements by residents. Residents themselves, in self-reflection, were the most potent drivers of reflection. Learning by doing and discussing clinical cases were considered potential strategies for the teaching-learning process, with doubt and the attempt to act serving as triggering and mobilizing elements of learning. There is a need to expand spaces, times, and opportunities to discuss situations of doubt and uncertainty that promote reflection on reflection-in-action, thus enabling more critical performances by residents. Teaching-learning situations should be promoted to enhance opportunities for students to undergo diverse experiences, which facilitate the accumulation of knowledge and experience repertoire by residents, preparing them to tackle the demands of the work routine.

## Supplementary Material

0034-7167-reben-77-04-e20230540-suppl01

0034-7167-reben-77-04-e20230540-suppl02

## Data Availability

https://doi.org/10.48331/scielodata.DXHQUL
