# Supplementary material for: Reflective practice of nurse residents in the teaching-learning process in teaching hospitals
Source: Rev Bras Enferm. 2024 Sep 6;77(4):e20230540. doi: 10.1590/0034-7167-2023-0540 (PMC11382670; doi:10.1590/0034-7167-2023-0540)
Supplement: 0034-7167-reben-77-04-e20230540-suppl01 [file 0034-7167-reben-77-04-e20230540-suppl01.pdf]

A prática reflexiva de enfermeiros residentes no processo ensino-aprendizagem em hospitais de ensino

Ana Carolina de Oliveira Paiva

Kênia Lara Silva

Neste volume de material suplementar apresentamos os dados das entrevistas utilizados para a produção da pesquisa reportada.

FONTES DAS ENTREVISTAS

O Quadro 1 apresenta a identificação dos residentes, com uma breve caracterização da sua posição no Programa de Residência em Área Profissional da Saúde (PRAPS). Neste quadro são apresentados também os trechos das entrevistas tomados como dados para a pesquisa.

Quadro 1 - Identificação dos residentes, caracterização no Programa de Residência em Área Profissional da Saúde e dados utilizados para a pesquisa.

Identificação	Caracterização do Residente	Dado utilizado para Pesquisa
R1<br><br>Legenda<br>P: Pesquisadora<br>R1: Residente 1	Residente do 1° ano do PRAPS modalidade Multiprofissional em Saúde do Idoso.	*P: Então, diante desses dois relatos, queria fazer as seguintes perguntas para você: como você percebe essas cenas de ensino-aprendizagem no cotidiano da sua residência? Tipo, se elas são comuns? Como que você percebe isso?*<br>*R1: Elas... Então, elas são... Elas não, não acontecem sempre ao longo do dia. É::, e também, tipo assim, elas são bem comuns, mas não, tipo assim, tem um período que a enfermaria tá..., que o setor tá mais corrido, aí fica mais complicado de a gente buscar... Como a gente tá no segundo cenário, né, da gente, tipo, pouco mais de seis meses, eu notei que no, nesse setor do cuidado paliativo a gente teve muito mais essa, essa parte de ensino-aprendizagem, de, de, do paciente tá lá, e a gente tá à beira-leito, ter essa parte de tirar dúvida, perguntar, de esclarecer melhor. Muito mais nesse setor, a gente tá sentindo isso. É::, mas assim, é lição. As vezes eu sinto falta mais de a gente sentar e:: preceptor e, e residente, e discutir mais os casos, sabe?*<br>*P: [[Uhum.*<br>*R1: Não apenas, não apenas de, de::, de no momento que precise... Por exemplo, não há apenas um momento de feridas, mas tipo assim... Porque como, o... Acaba ficando muito corrido, né, tipo, não tem como não ficar corrido,*

		porque são muitos pacientes, é::, acaba que tem paciente que a gente não consegue aprofundar, tipo, entender melhor o caso dele, sabe? Por que é que ele tá com uma IC descompensada e, e:: tá com uma::, uma::, sei lá, uma úlcera venosa, sabe? Coisas desse tipo, eu sinto falta de, dessa discussão, mais assim, de trabalhar mais essa questão dos diagnósticos de enfermagem, das intervenções, não ficar meio::, só aquela, a situação de repetição, sabe? Acaba que esse processo de evoluç..., de sempre evoluir os pacientes, acaba deixando a gente muito preso, de tem que dar conta de evoluir tantos pacientes e acaba que a gente não consegue fazer discussão de caso. Na verdade, eu não consigo imaginar como seria diferente, sabe? Com uma realidade que seria diferente, assim. Pra gente buscar, eu acho que os dias de corrida de leito é ajuda, que, é::, que são os dias das reuniões, ajudam bastante a gente a esclarecer melhor, compreender melhor o, o::, o quadro de um paciente X ou Y que chega. Mas, sim, eu sinto isso, esse momento de ensino-aprendizagem nesse setor, eu tenho sentido muito mais forte do que no outro. No outro eu nem tinha. Só que eu acho que a demanda do outro setor era bem mais, bem mais baixa, sabe, assim. E também era uma realidade bem diferente daqui, né?<br><br>*P: Sim. É::. E quais estratégias de ensino-aprendizagem você vivencia em seu cotidiano como residente?*<br>*R1: (Não sei), estratégia de ensino aprendizagem...*<br>*P: Tipo, técnica, atividade, quais são as atividades aqui? Tipo, eu percebo que você executa técnica. Você disse que você observa. Tem alguma outra técnica de ensino-aprendizado? Como que você aprende aqui dentro?*<br>*R1: Eu aprendo muito, muito não, algumas vezes eu aprendo pegando uns técnicos, por exemplo. É::, resgate, doses de resgate, morfina. Que é uma coisa totalmente fora da minha realidade, lá do hospital escola que eu vim. Entã::o, a técnica de ensino-aprendizagem é assim: "hu::m, nunca fiz isso. Vou fazer" (risos). Aí eu ponho a mão na massa e faço, sabe?´*<br>*P: [[Uhum.*<br>*R1: Aí ( ) e faço, sabe?*<br>*P: [[Aham.*<br>*R1: É::, no restante também, tipo assim, aqui eu encontrei muito paciente co::m cirurgia de períneo, e essa cirurg., e assim, uma... muita dificuldade pra encontrar a uretra.*<br>*P: [[Uhum.*<br>*R1: Entã::o, a, a, o ensino-aprendizagem foi muito na prática mesmo, tipo...*<br>*P: Uhu::m.*<br>*R1: Vai ter...*<br>*P: [A prática cê fala, executar... fazer.*<br>*R1: É, executando a técnica.*<br>*P: [[Uhum.*<br>*R1: Tipo, é::, todas as vezes que eu tive dificuldade com, co::m paciente daqui. Eu não tenho muito já esse, assim, que eu (cho), que eu meio que chego toda hora com dificuldade, sabe? Porque::, surge sonda, ai eu peço pra passar, então eu vejo o paciente da sonda... com uma sonda, com uma sonda::, tem uma cirurgia de períneo, como eu te falei.*<br>*P: [[Aham.*

		R1: Aí eu tenho dificuldade, eu chamo Rafaela, "ô Preceptora Y, tô com dificuldade".<br>*P: [[Uhum.*<br>*R1: Aí ela vai me norteando, sabe?*<br>*P: [[Uhum.*<br>*R1: "Lembra que eu te falei sobre isso, cirurgia de períneo e tal...". Aí pede pra eu procurar, e até pra ela também acaba sendo uma novidade, né?*<br>*P: [[Sim.*<br>*R1: Já tive pacientes aqui co::m, é::, com CA de útero, e aí, um câncer de útero, né? E:: ela tava::, e ai quando eu fui passar a sonda nela, eu jurava que ela era muito mais jovem que eu, inclusive, então eu achei que ia ser super tranquilo passar a (sonda) ((risos)). Cheguei lá, a mulher ainda tava numa maca ((risos)), uma maca, isso, aí eu pedi ajuda da Preceptora Y, aí Preceptora Y veio na... ela foi pra paciente, parece. Ela gosta de ensinar. Preceptora X também. Então, foi be::m, bem isso. É::, executando a técnica, que eu aprendi.*<br><br>*P: No seu dia-a-dia, assim, o que você acha que te faz mais refletir?*<br>*R1: O que eu mais paro e fico pensando é quando:: alguma coisa que dá certo com todo paciente, não tá dando certo com aquele. Com um paciente X, por exemplo. Paciente:: (+) tem alergia, tá dando algum tanto, um tipo de alergia, alguma rejeição à hipodermóclise. Aí eu acho interessante, isso fica::, isso:: acaba, isso desperta curiosidade em mim, sabe? É::, ou se não quando:: (+) vai ser sempre bem da, do paciente. Tipo assim, evoluir é uma coisa que eu gostava muito de fazer antes. Ma::s hoje tá, hoje tá se tornando cansativo pra mim, sabe? Evoluir. Porque é muito repetitivo.*
R2<br><br>Legenda<br>P: Pesquisadora<br>R2: Residente 2	Residente do 2° ano do PRAPS modalidade Uniprofissional Intensivismo, Urgência e Trauma.	*P: Então, diante essas duas cenas, E a sua vivência também como residência, é::, como que você percebe essas cenas de ensino-aprendizado no cotidiano da sua residência?*<br>*R2: (+) Deixa eu pensar (+).*<br>*P: (Cê) percebe, tipo, elas são comu::ns?*<br>*R2: São comuns. Em muitas situações, às vezes, a gente TENTA resolver, mas a gente tem a dúvida.*<br>*P: Uhum.*<br>*R2: E aí a gente procura, né? Pela... Por quem é a nossa referência.*<br>*P: Uhum.*<br>*R2: Na situação 1 ((risos)), que foi o que aconteceu comigo, né? ((risos)) Eu lembro exatamente que a minha dúvida, na verdade, era se eu poderia tentar lavar ou se eu tinha que aspirar.*<br>*P: Uhum.*<br>*R2: Então eu preferi não fazer.*<br>*P: [[Uhum.*<br>*R2: Mas é isso. Acho que na... Geralmente, a gente se vê em situações, assim, de dúvida mesmo...*<br>*P: [[Uhum.*<br>*R2: E aí, a gente precisa do auxílio do preceptor.*

		P: É::, e quais estratégias de ensino-aprendizagem você vivencia em seu cotidiano de trabalho como residente? (+) Quais são as estratégias que, que é utilizada no dia-a-dia, pra você aprender?<br>*R2: Ah, no dia-a-dia eles, a, eles colocam a gente, né, assim, pra assumir mesmo. Então, na medida das demandas do paciente, vão ocorrendo dúvidas e é::, eu vou levando essas (+).*<br>*P: [[Uhum.*<br>*R2: Essas dúvidas pro preceptor. Ma... E...Tem alguns também que vêm e falam: "olha, tem um artigo tal, que fala sobre isso", e aí geralmente me encaminha, né?*<br>*P: [[Uhum, uhum.*<br>*R2: Então assim, eu vejo situações assim de aprend..., de ensino-aprendizado. Eles confiam a você, a, o cuidado de algum paciente, e aí: "cê tem dúvida?". NÃO FAÇA, não faça com dúvida.*<br>*P: [[Uhum. uhum.*<br>*R2: Tô, eu tô aqui! Então eu vejo muito assim. Então, como eu tô no R2, é::, eu percebo um pouco de diferença nisso também. Eles são mais autonomia pra gente.*<br>*P: Uhum.*<br>*R2: E aí::, se eu tenho dúvida, eu tenho com quem, pra, a quem perguntar, né, recorrer.*<br>*P: [[Sim, sim.*<br>*R2: Mas é, mas a maioria é::, das situações de ensino-aprendizado, eu vejo que sou eu indo atrás do preceptor, né?*<br>*P: [[Uhum, sim.*<br>*P: É::, elas permitem aprender a lidar com cenas como as relatadas? Essas estratégias de ensino-aprendizado do, do dia-a-dia, elas permitem aprender a lidar com essas cenas?*<br>*R2: Sim. Sim. Vejo que sim. É:: (+) O que eu ia falar? (+) Assim, eu, no dia-a-dia, eu vejo que, com as cenas relatadas, sim, eu aprendo a lidar, re, resolver aquelas situações.*<br>*P: [[Uhum.*<br>*R2: Que as vezes vai se... vai ter alguma coisa que é novidade pra mim, mas que pode vim a acontecer novamente, com uma certa frequência, posteriormente, né? Então eu vou aprender a lidar.*<br>*P: [[Uhum.*<br>*R2: E aí, eu vejo muito que é isso. Às vezes, a gente tá ali na assistência, a gente aprende muito a esse, esse fazer.*<br>*P: [[Uhum.*<br>*R2: A resolver aquele problema, né?*<br>*P: [[Uhum.*<br>*R2: E nem sempre entender o que que tá por trás ((risos)). Mas a gente aprende, sim.*<br>*P: É::, quais estratégias você acredita ser potencial, ser ideal, para o processo de ensino-aprendizado na residência?*<br>*R2: (+) É::, é::, tem uma cobrança muito, assim, em relação "a::h, vamos fazer estudo de ca::so", é::, então é o residente apresenta um estudo de caso.*<br>*P: [[Uhum.*

		R2: Mas não... Eu sinto falta da questão do APROFUNDAMENTO fisiopatológico me::smo, sabe? Assim, essa discussão dos porquês tal doença desencadeou outra coisa, e tal. E aí, e::u acho que (+) uma estratégia (que) talvez fosse melhor, que agregaria mais, fixaria melhor o conhecimento, seria as discussões em, é, dentro do setor mesmo, sabe?<br>*P: [[Uhum.*<br>*R2: É::, envolvendo, é::, discussão mesmo DA situação da clínica do paciente, é::, tanto do ponto de vista DO residente, quanto de vista, o ponto de vista do preceptor, mesmo.*<br>*P: [[Uhum, sim.*<br>*R2: Então acho que seria:: uma estratégia mais ideal (assim).*<br>*P: [[Sim.*<br>*R2: Pra ensinar, (pensar).*<br>*P: Sim. É::, e quais as situações do seu dia-a-dia que você acha que mais estimula a sua reflexão?*<br>*R2: (+) Eu acho que é quando EU tô no, no computador pra EVOLUIR, e aí, eu sempre busco ver qual é::, a abordagem de outros profissionais frente àquele paciente também. Então, eu tenho o meu cuidado. E aí, eu tento ver, é::, (+) entender mesmo, assim, todo o contexto do paciente, né? Então, eu acho que, assim, quando eu tô mesmo ((risos)) (+). Eu vou se... Eu avalio o meu paciente e vou sentar pra evoluir, eu tento entender toda a história de::le, né? Então eu acho que ((risos)) (é) mais quando eu tô na frente do computador.*<br><br>*P: Até nas estratégias de ensino-aprendizado, mesmo. Que que cê acha que... Alguma atividade que poderia te oferecer, ou então algo que você poderia executar, que você acha que refle, refletiria mais, pra você aprender mais?*<br>*R2: Eu acho que seria (+), deixa eu pensar. Porque tem muita coisa, assim. A gente, n, na residência a gente tem essa FALTA, assim, dessa parte teórica de aprendizado. Então eu acho que, não só... Igual, eu, como eu citei, a questão das discussões, né? Em setor. Mas eu acho que, também, direcionar conteúdos de eixo específico, igual da minha residência, que é intensivismo, urgência e trauma.*<br>*P: [[Uhum.*<br>*R2: Ah, eu, eu acho que se eles fornecessem pra gente, ah, conteúdos, mesmo, de aprofundamento teórico, seria, é::, interessa::nte. Assim (+), ajudaria bastante nesse processo de unir, né? Prática e teoria.*<br>*P: [[Uhum.*<br>*R2: É::, não sei. Talvez tentar participar de cu::rsos, també:m, né?*<br>*P: [[Uhum, uhum.*<br>*R2: De aprofundame::nto. Acho que é isso.*
R3<br><br>Legenda	Residente do 1° ano do PRAPS modalidade	*P: Uhum. É:: Diante::, diante uma situação de incerteza, ou complexa, igual essa que a gente descreveu, ou então alguma outra, como você age?*<br>*R3: Eu sempre vou em busca de um preceptor pra tirar minhas dúvidas, sempre.*

P: Pesquisadora<br>R3: Residente 3	Uniprofissional<br>Enfermagem Obstétrica	*P: E o que que você acha que poderia FAZER pra estimular a sua reflexão? O preceptor fazer… Alguma estratégia de ensino-aprendizado que você acha que você reflete mais?*<br>*R3: Eu acho que os estudos de caso, mesmo. Pegar um caso e a gente falar sobre as, as possíveis complicações daqui::lo… Ou possíveis desfechos positivos. Que exames a gente pode ficar mais ate::nto, como interpreta::r. Acho que essas coisas que vai, vai realmente estimular, assim, o raciocínio da gente.*
R4<br><br>Legenda<br>P: Pesquisadora<br>R4: Residente 4	Residente do 1° ano do PRAPS modalidade Uniprofissional Intensivismo, Urgência e Trauma	*P: E:: outras cenas, né, que você vivencia na NA residência, eu te pergunto, é, como que você percebe essas cenas de ensino-aprendizado no cotidiano da residência? Se elas são comuns, cê acha que elas não são comuns? Como que você percebe isso?*<br>*R4: Uhum. Ah, eu acho que geralmente é bem tranquilo essa, essa parte do ensino, assim. Geralmente eu me sinto bem confortável, é, por exemplo, de, de se eu tiver alguma dúvida, de perguntar. Às vezes, quando eu, o trabalho tá meio, tipo, tem muita coisa pra faze::r, ou tá tendo muita intercorrência, assim, eu às vezes acabo que fico com mais de receio de perguntar, as vezes com medo de, de as vezes tá atrapalhando, né. Mas eu acho que é bem tranquilo, assim. Tipo, igual a Preceptora Z deixa claro que não é pra gente fazer nada sem, é::, se não tiver, se tiver dúvida. Geralmente eu pergunto, e geralmente tem bastante, tipo, é::, eles ensinam bastante a parte técnica, assim. Igual, medicação, (também), que eu tenho bastante dúvida, sempre que eu pergunto, sempre respondem.*<br><br>*P: Uhum. É::, quais estratégias de ensino-aprendizagem você vivencia em seu cotidiano como residente?*<br>*R4: Eu acho que tem essas partes PRÁTICAS, mesmo, de, de chegar e questionar alguma dúvida que eu tenho, é, quando a gente tá em procedimento, né, então eu acho que é isso, é, essa questão de expor pra ver mais em procedimentos, que ai vai aparecendo as dúvidas, eu acho que essa é uma estratégia. Am.. E as partes teóricas. Geralmente a parte teórica a gente não tá tendo muito, mas eu acho que tipo, é o::, essas discussões que a gente, que, que teve lá na ( ). Acho que são essas (partes), essas estratégias.*<br>*P: Hoje, hoje, de teoria, cê tá tendo a tutoria?*<br>*R4: Teria... Agora vai ter, é... Agora vai ter só a tutoria, tipo. Só de aula mesmo, mas não é, assim, não é muito voltado pra parte prática de enfermagem. A gente tá vendo mais num contexto, é, um contexto geral. Então, a gente tá vendo mais sobre gestão do cuidado, assim.*<br>*P: Uhum.*<br>*R4: Não é nada, tipo, direcionado PRA parte prática de enfermagem. Ai, como acaba que não tem muito essa parte prática, meio que tento compensar em casa, é, tipo, tentando estudar no final de semana.*

Fonte: Elaborado pelas autoras.
