# Supplementary material for: Reflective practice of nurse residents in the teaching-learning process in teaching hospitals
Source: Rev Bras Enferm. 2024 Sep 6;77(4):e20230540. doi: 10.1590/0034-7167-2023-0540 (PMC11382670; doi:10.1590/0034-7167-2023-0540)
Supplement: 0034-7167-reben-77-04-e20230540-suppl02 [file 0034-7167-reben-77-04-e20230540-suppl02.pdf]

A prática reflexiva de enfermeiros residentes no processo ensino-aprendizagem em hospitais de ensino

Ana Carolina de Oliveira Paiva

Kênia Lara Silva

Neste volume de material suplementar apresentamos os dados das observações utilizados para a produção da pesquisa reportada.

FONTES DAS OBSERVAÇÕES

O quadro 1 apresenta as descrições das atividades desenvolvidas pelo Residente 1 (R1) do primeiro ano do Programa de Residência em Área Profissional da Saúde (PRAPS) Modalidade Multiprofissional Saúde do Idoso em 30 horas de observação.

Quadro 1 – Descrição das atividades desenvolvidas pelo R1 em 30 horas de observação.

Data Observação	Horário Início	Horário Término	Setor Observação	Descrição das atividades realizadas pelos residentes
02/08/2022	07:40	09:50	Idoso Frágil e Paliativo	R1 avalia paciente 1 sob sua responsabilidade. Conversa com os pacientes e acompanhantes; pergunta detalhes sobre a condição clínica dos pacientes, alimentação, lesão, etc.; orienta sobre prevenção de lesão e importância da ingestão de água. Além de confortar os pacientes por meio da escuta. Durante o exame físico dos pacientes, R1 realiza anotações na prancheta. Enquanto isso preceptora desenvolve outras atividades.
				R1 avalia paciente 2 sob sua responsabilidade. Conversa com os pacientes e acompanhantes; pergunta detalhes sobre a condição clínica dos pacientes, alimentação, lesão, etc.; orienta sobre prevenção de lesão e importância da ingestão de água. Além de confortar os pacientes por meio da escuta. Durante o exame físico dos pacientes, R1 realiza anotações na prancheta.
				R1 avalia paciente 3 sob sua responsabilidade. Conversa com os pacientes e acompanhantes; pergunta detalhes sobre a condição clínica dos pacientes, alimentação, lesão, etc.; orienta sobre prevenção de lesão e importância da ingestão de água. Além de confortar os pacientes por meio da escuta. Durante o exame físico dos pacientes, R1 realiza anotações na prancheta.
				Paciente 4 reclama sobre dor nas costas e solicita orientação para que alivie, assim R1 faz vários tipos de perguntas no intuito de entender o motivo e o local exato da dor à medida que realiza o exame físico minucioso na região. Não encontra nada e em seguida escreve na prancheta algumas observações, neste momento R1 faz uma pausa seguida da seguinte fala para a paciente: "irei conversar com a TO para fazer coxins na tentativa de aliviar suas dores nas costas". Além disso, solicita ao TE que se encontrava a beira leito para chamá-la no momento do banho para avaliar e fazer os curativos das LPP.

				Ao avaliar o paciente 5, R1 fala (pensando alto) que o acesso venoso periférico se encontra sem identificação, assim R1 começa a fazer perguntas para o paciente na tentativa de descobrir o dia da punção do acesso, após várias perguntas, descobriu a data de punção do acesso.
				Avalia paciente 6 e percebe que o mesmo se encontra com dor de ouvido, pergunta ao paciente se está com dor no ouvido e o mesmo responde que sim. R1 faz perguntas sobre qual o ouvido, o tipo de dor, se tem cheiro, secreção, etc. À medida que paciente responde suas perguntas, faz novas perguntas a fim de entender o problema do paciente. Ao final, faz anotações na prancheta e diz que solicitará avaliação médica.
				Avalia um paciente italiano, R1 expressa sua dificuldade para conversar com ele, tentativas de diálogo por meio de gestos, mas em um determinado ponto desiste e começa a fazer perguntas para a cuidadora do paciente, que também tem dificuldades de entendê-lo. Neste momento, TE chama R1 para avaliar e fazer o curativo de uma LPP, então a mesma solicita licença e vai em direção ao TE.
				R1 avalia LPP enquanto TE diz que está usando petrolato no local, R1 concorda com o TE e vai buscar os materiais do curativo. Enquanto realiza a higienização e troca do curativo, R1 comenta sobre a evolução da LPP com o TE, dizendo o quanto melhorou e aponta, com o dedo, para a ferida à medida que fala; pergunta a paciente se a mesma sente dor no local; e acompanhante inicia uma conversa com R1 perguntando como será o tratamento da LPP em casa e questiona o motivo de ter trocado a utilização de alginato para petrolato. R1 explica com detalhes o motivo da troca do alginato para o petrolato e em seguida explica a acompanhante o modo de fazer o curativo em casa.
				Preceptora chega ao final do curativo de R1 e diz que irá fazer um debridamento de uma LPP com a R0 e que é para R1 avisar ao TE X que o paciente Y será encaminhado para realização de Ecocardiograma transesofágico. Assim, preceptora vai para o leito iniciar o debridamento com R0 e R1 dá o recado ao TE X e em seguida, vai observar o debridamento que a preceptora e R0 estão fazendo. R0 realiza a técnica de debridamento, enquanto preceptora explica e orienta com detalhe a ação. R1 escuta a explicação, observa o procedimento com atenção, direcionando a cabeça e o olhar para o local onde R0 coloca a pinça. R1 Faz perguntas sobre como desenvolver a técnica. Em um determinado momento, R0 precisa trocar de pinça para dar continuidade no procedimento, então preceptora pergunta a R0 qual a melhor pinça a ser utilizada naquele momento, R0 responde e reinicia o procedimento, ao reiniciar, preceptora mostra as alunas a pinça que prefere utilizar para aquela determinada técnica e explica o motivo. Durante toda a execução do debridamento as três conversam sobre o desenvolvimento da técnica e sobre a LPP. Ao final, R0 com dificuldade para debridar o tecido fino e profundo, a preceptora assume a técnica e começa a executar o debridamento enquanto as 2 residentes observam e escutam a explicação da preceptora, acompanham a pinça com o olhar. Em seguida, ao realizar o curativo da LPP, R0 tem dificuldade de inserir o alginato na cavidade da lesão, R1 orienta R0 utilizar 2 pinças, pois acredita que isso irá facilitar o manuseio da placa de alginato. R0 não consegue, então preceptora realiza o curativo, orientando a melhor forma de fazê-lo.
				R1 segue com os exames físicos dos pacientes, chega no paciente 8 que está recebendo banho de leito da TE, R1 observa petéquias no corpo do paciente e comenta com TE que o paciente recebeu plaquetas na semana passada devido a plaquetopenia e as petéquias se mantêm. R1 tira foto das petéquias. Inicia as evoluções no sistema eletrônico. Ao ver preceptora, R1 a pergunta se lembra do paciente que foi transfundido com plaquetas semana passada, enfermeira diz que não. Então, R1 mostra a foto que tirou das petéquias e confirma com preceptora se aquilo é realmente petéquias, preceptora confirma.

16/08/2022	13:00	19:30	Idoso Frágil e Paliativo	Enquanto enfermeiras do plantão manhã e tarde realizam a transferência de cuidados, R0 e R1 escutam os casos e complementam informações de pacientes quando necessário ou quando enfermeira manhã faz perguntas diretamente a elas referentes a pacientes. Durante a transferência de cuidados conversam assuntos não relacionados ao serviço. R1 durante este período organiza caixas de curativos e utiliza celular em alguns momentos.
				R0 e R1 conversam sobre TCR que estão construindo sob orientação da tutora. Comentam que precisam realizar pelo menos uma entrevista para apresentar a tutora na próxima reunião online. Leem o TCLE e o roteiro de entrevista (ficam muito tempo em silêncio). R1 critica o roteiro de entrevista, fala para R0 os possíveis vieses, sobre a dificuldade dos pacientes e familiares de entenderem o que é hipodermóclise. R0 também demonstra descontentamento com o trabalho. Olham a lista de pacientes internados no setor e avaliam os casos clínicos dos pacientes que poderiam ser incluídos na pesquisa. Fazem a relação dos critérios de inclusão e exclusão com os casos clínicos do paciente. Chegam à conclusão de que apenas um paciente atende os critérios de inclusão e exclusão, programam entrevistar o familiar no decorrer da tarde.
				Evolução no Sistema Eletrônico. R1 se senta na sala e inicia sua evolução, diz a preceptora que precisa evoluir os pacientes e procedimentos que aconteceram no turno da manhã. Enquanto evolui, TE chega na sala e lê para a R1 as medicações que paciente está tomando e reclama que elas não resolverão a agitação do paciente. Nesse momento, outro TE chega na sala e fala que não administrou a medicação x deste paciente, pois ficou inseguro por ser uma dosagem alta. R1 diz a eles que médica x deixou claro que era para administrar todas as medicações prescritas, no entanto TE diz que trocou uma ideia com a médica y sobre a dosagem da medicação e a mesma concordou em não administrar. R1 fica calada e continua sua evolução. TEs saem da sala.
				R1 procura TE no corredor e pergunta aspecto de LPP, a mesma diz que não trocou o curativo pois paciente solicitou não o movimentar uma vez que está nauseado e com medo de voltar a vomitar. R1 retorna para evolução. Durante alguns momentos da evolução, R1 utiliza celular, passa a mão na cabeça e fala que está com sono.
				R0 chama R1 para vê-la passar CVD. R1 e R0 reúnem material e colocam na mesa de procedimento, enquanto R0 higieniza as mãos, R1 abre os materais na mesa de procedimento e em seguida posiciona mesa próxima a cama do paciente. R0 orienta paciente e ao abrir a fralda visualiza fezes, então inicia higienização. R1 percebe que não tem fralda, sai da enfermaria para buscar. Retorna com a fralda e entrega para R0, em seguida reúne os lixos e descarta. R0 durante a higienização da região uretral, visualiza o canal uretral, então mostra para R1 a localização. R1 se posiciona no melhor local da cama e inclina o corpo para conseguir visualizar o canal uretral. Enquanto isso, R0 conta a R1 sobre o motivo da dificuldade de passar a sonda nesta paciente no dia da admissão. R1 observa canal uretral e escuta atentamente o que R0 conta. Em seguida, R1 utiliza o celular para iluminar o canal uretral e diz para R0 para clampar a bolsa coletora a fim de evitar o vazamento da diurese para o chão. R0 inicia a passagem da sonda enquanto R1 mantem-se atenta e com o corpo inclinado para visualizar o procedimento, assim que retornou diurese pelo sistema, R1 diz "você brilhou" e sai do leito para buscar micropore. R1 retorna com um pedaço de micropore e entrega a R0 para fazer fixação do CVD no paciente. No meio do procedimento, preceptora chega e diz que irá trocar bolsa de NPT, R1 e R0 pedem a ela para aguardar um pouco, pois querem ver, preceptora diz que vai aguardá-las por 10 minutos e sai do leito. R1 vai ao encontro da preceptora e diz que o procedimento acabou.

				Preceptora e R1 vão para a sala de preparo de medicações, enquanto preceptora reúne materiais, faz desinfecção da bandeja e conversa com TEs, R1 fica lendo o rótulo da bolsa de NPT. Ao finalizar, as duas caminham para o leito do paciente. Enfermeira orienta paciente e inicia a troca da bolsa de NPT, enquanto faz o procedimento, preceptora vai explicando os passos para R1. R1 observa atentamente e vai se aproximando da preceptora e da BI a fim de facilitar a sua visualização.
				Ao finalizar a troca da bolsa de NPT, TE solicita a preceptora para avaliar uma lesão no paciente ao lado. Ao ver a lesão, diz a R1 que de acordo com aquelas características tinha uma sugestão forte de ser erisipela, descreve as características para R1 que se aproxima para visualizar melhor a lesão. R1 pergunta para a preceptora como seria o tratamento. Enfermeira diz que irá conversar com médica para definir o melhor tratamento. Assim, TE vai posicionando paciente e as duas saem do leito conversando sobre a lesão.
				R1 evolui na sala de evolução, preceptora chega e também começa a evoluir. Em diversos momentos, chegam TEs e tiram dúvidas com a preceptora, mas R1 mantém-se focada em sua evolução. Não interage com as conversas. R0 chega e chama R1 para entrevistar um familiar para a pesquisa de TCR. R1 diz que irá apenas finalizar sua evolução. Finaliza, imprime e guarda evoluções em prontuários.
				Ao finalizar evolução, R0 entrega a R1 uma placa de identificação de paciente que está sob a responsabilidade de R1 e diz que está identificando os leitos de alguns pacientes. R1 diz "nossa, eu nunca faço isso”. Então, R1 vai ao leito e cola placa de identificação do paciente, R1 aproveita e passa nos demais leitos para verificar se todos contém placa de identificação.
				R1 e R0 conversam sobre paciente que irão entrevistar, sobre o roteiro de entrevista e em seguida combinam a forma de conduzir entrevista. Combinam de R1 fazer as perguntas e R0 acompanhar e complementar se necessário. Chegam ao leito da paciente e se apresentam ao acompanhante. R1 apresenta a pesquisa para ele que responde ser necessário solicitar autorização para sua esposa que não está no hospital naquele momento. Então R1 explica da simplicidade da pesquisa, fala que não precisará de informações da paciente, apenas de sua opinião sobre a hipodermóclise que está puncionada na paciente. Enquanto isso R0 se posiciona no pé da cama e fica fazendo carinho nos pés da paciente. Não diz nada. Ao final da explicação, acompanhante concorda em participar, assim o encaminham para uma sala reservada. R1 faz toda a condução da entrevista. Ao se sentarem na sala, R1 explica e solicita acompanhante a ler o TCLE, enquanto isso R1 e R0 ficam em silêncio e utilizam o celular em alguns momentos. Após assinatura do TCLE, R1 inicia gravação da entrevista e o acompanhante à medida que vai respondendo as perguntas, R1 faz algumas anotações. Durante toda a entrevista R1 permanece atenta, em alguns momentos movimenta a cabeça (expressão de concordar com a fala), coloca a mão no queixo e seu olhar sempre direcionado ao acompanhante. Entrevistado em um momento esquece o nome da médica do plantão, R1 rapidamente diz o nome. Ao finalizar a entrevista, R1 e R0 agradecem e o entrevistado sai da sala. Em seguida fazem comentários sobre a experiência de ter entrevistado o acompanhante, R1 diz que achou tranquilo e que gostou das respostas do acompanhante. Disse ainda que as respostas foram tão completas que pulou algumas perguntas do roteiro, uma vez que o acompanhante foi respondendo antes. As duas sorriem com satisfação.
				Saem da sala onde aconteceu a entrevista e vão avaliar a paciente do acompanhante entrevistado. Ao chegarem, acompanhante mostra a hipodermóclise (HP) e o local onde disse na entrevista que está o edema, R1 avalia edema, aperta o local, pergunta se está doendo, passa a mão no local e em seguida explica ao acompanhante que o edema não está relacionado ao HP, explica os motivos e vai mostrando a pele para ele. Acompanhante mostra uma infiltração de acesso no MSE e R1 pergunta se foi acesso venoso ou HP, ele diz não saber. R1 vai fazendo perguntas a ele para tentar descobrir, ao final conclui que a infiltração foi proveniente de acesso venoso. R1 e R0 saem e caminham para o posto de enfermagem.

				Enfermeira do plantão noturno chega e todas vão para a sala de supervisão de enfermagem, conversam assuntos não relacionados ao trabalho e vão trocando de roupa. Em seguida, enfermeira do noturno e do dia se sentam em cadeiras onde está a mesa da supervisão, R1 se senta ao lado da enfermeira do dia e R0 fica encostada na bancada. Inicia-se a passagem dos casos clínicos pela enfermeira do dia, R1 continua organizando seus pertences na mochila, utiliza o celular e em alguns momentos complementa a fala da enfermeira em relação as informações dos pacientes. Além disso, dependendo da situação diz: "sem condições", "precisa conter".
19/08/2022	07:00	13:00	Idoso Frágil e Paliativo<br><br>Aula Teórica - Auditório	Chego ao plantão com a transferência de cuidados em andamento. Presentes na sala enfermeira do noturno, diurno e 2 residentes. Enfermeiras na mesa passando os casos e residentes sentadas em cadeiras mais afastadas escutando os casos. Em alguns momentos R1 que está sentada mais próxima a enfermeira preceptora inclina o corpo para visualizar o que a preceptora escreve em passômetro e em outros momentos complementa as informações de alguns pacientes. Em um determinado momento, enfermeira do noturno destaca para a do diurno sobre dose elevada de morfina que paciente está tomando, R1 neste instante pergunta em voz baixa para a preceptora a dose usual que pacientes recebem morfina, preceptora responde e R1 diz "nossa, então está muito alta mesmo". Ao final da transferência de cuidados, enfermeira do noturno sai da sala e preceptora coloca uniforme e conversam assuntos aleatórios com as residentes.
				R1 abre as caixas de curativos e inicia o levantamento das coberturas que precisa solicitar no sistema para o dia. Então olha nas caixas de coberturas o que já tem disponível e avalia na lista de pacientes internados na unidade aqueles que tem lesões. Assim, à medida que faz o levantamento, R1 fala para a preceptora a quantidade que solicitará e preceptora vai concordando. Em alguns momentos pergunta à preceptora a quantidade que ela costuma solicitar em sua rotina de trabalho. Preceptora em alguns momentos responde, mas acrescenta que estava afastada e consequentemente não conhece os pacientes, é para solicitar o que achar pertinente. Ao final, R1 vai para o computador e inicia as solicitações das coberturas no sistema eletrônico. Imprime, assina e solicita TE para buscar.
				R1 e R0 avisam para preceptora que estão descendo para o auditório no 1º andar para assistir a aula teórica sobre o controle da dor. Ao chegar no auditório, aula já em andamento, R1 assenta em cadeira no meio da sala, local onde está vago. (não levou papel e nem caneta para realizar anotações). Auditório com uma média de 30 residentes da equipe multiprofissional, incluindo médicos. R1 durante grande parte da aula mantém-se com braços cruzados e olhando para a apresentação, em alguns momentos se movimenta na poltrona. Ao final da aula, abre para discussões e perguntas, vários residentes se pronunciaram, R1 mantém-se em silêncio. À medida que os alunos faziam as perguntas, R1 se movimentava na poltrona para identificar a pessoa que se pronunciava. Ao finalizar a aula, R1 e R0 saem juntas da sala e caminham no sentido de retornar para o setor. No percurso R1 elogia a aula e discutem problemas que veem no dia a dia e que foi discutido durante a aula.
				Ao retornar para o setor, vê que os materiais de curativo solicitado mais cedo já chegaram ao setor, então certifica se não está faltando nada, neste momento R1 identifica que por mais atenção que teve ao fazer as solicitações, ainda esqueceu de pedir gaze.
				R1 aproveita que o plantão está tranquilo e tira uma dúvida com a preceptora sobre os volumes das medicações que podem ser infundidas por meio da hipodermóclise. Preceptora explica para R1 e aproveita e exemplifica com um caso de um paciente internado na unidade. Enfermeira do cuidado paliativo chega durante a explicação e se envolve no assunto, aproveita e expõe para a preceptora os problemas que está tendo com o referido paciente para controlar sua dor. Relata sua preocupação com as elevadas doses de analgésicos que está recebendo e troca uma ideia com a preceptora da implementação de terapias não medicamentosas no tratamento do paciente. Durante a conversa entre preceptora e enfermeira, R1 permanece atenta e expõe sua opinião durante a conversa. As três permanecem em pé no meio do corredor conversando sobre os problemas que o referido paciente está gerando para a equipe. Em um determinado momento, R1 coloca sua mão no braço da preceptora e

				pergunta novamente sobre o volume das medicações administradas através de HP (neste momento, sinto que R1 ainda não sanou suas dúvidas sobre o assunto). Preceptora responde de forma suscinta e retorna sua conversa com a enfermeira do cuidado paliativo que finalizam a conversa definindo solicitar ajuda da psicologia. Então, enfermeira do paliativo vai embora e R1 leva preceptora para a sala de medicações e mostra a ela o cartaz sobre as medicações que podem ser infundidas na HP, em seguida retoma a pergunta inicial sobre volumes das medicações em HP.
				Separa material para punção de HP, aparentemente confiante no preparo, aspira soro fisiológico, coloca álcool em gaze. R1 já no leito do paciente começa a explicar detalhadamente a técnica de punção de HP para TE, fala sobre os locais indicados para a punção, posição do jelco, volume a ser administrado para avaliar a permeabilidade, diz ao TE que irá puncionar na região de anterior de coxa. Preceptora chega e R1 diz a ela que irá puncionar na porção de anterior de coxa, então preceptora pergunta: "porque não punciona no vasto lateral da coxa? Tem mais tecido adiposo aqui!". R1 diz a ela os motivos que prefere puncionar em anterior de coxa, no entanto preceptora fala que vasto lateral da coxa está com mais tecido adiposo, sendo mais seguro a técnica, em seguida sai do leito para atender o telefone. R1 então diz pensando alto: "se ela disse, irei puncionar na lateral". R1 executa o procedimento e ao final injeta 1 ml de soro fisiológico no acesso a fim de checar permeabilidade. Liga bomba de infusão, programa infusão de medicação e sai do leito e segue para outra punção de HP. Demonstra confiança na execução da técnica.
				Enfermeira preceptora, R1 e R0 ficam no posto de enfermagem e preceptora começa a explicar a elas os cuidados com as medicações que podem ser administradas na HP. R1 não demonstra interesse na conversa, usa o celular diversas vezes. Ao final, R0 inicia uma conversa no sentindo de encontrar um tema para seu projeto de TCR, conversam sobre higiene bucal, diabetes, fratura de fêmur e LPP. R1 calada na conversa. Ao entrar no assunto de LPP, preceptora envia um folder para o WhatsApp das residentes sobre lesão de kennedy, abrem o folder no formato online e começam a ler. R1 se demonstrou interessada, começa a comentar sobre as figuras do folder e ao final pergunta para preceptora sobre o tratamento destes tipos de lesão.
				Aproveitam o momento de conversa com a preceptora, então R0 pergunta sobre o cateter duplo J, associando ao caso de um paciente internado. R1 animada diz: "quero saber também". Começam a conversar sobre o caso do paciente, então preceptora aproveita que médica passa pelo corredor e a chama para detalhar mais sobre o conhecimento do cateter. Médica solicita, explica com detalhe o caso da paciente. R1 atenta a conversa, faz perguntas a médica sobre o assunto, durante a conversa franze a testa (R1 transparece estar achando difícil entender a explicação), diz "irei estudar isto em casa". Residente de medicina para no corredor e fica escutando a explicação também.
				R1 se senta no computador e inicia suas evoluções.
22/08/2022	07:00	13:20	Idoso Frágil e Paliativo	Preceptora atrasada, R1 pega todos os casos da enfermeira do plantão noturno, anota em passômetro os pontos relevantes. Em outros momentos pergunta se paciente x irá de alta, se paciente y irá realizar a tomografia hoje. Quase no final da transferência de cuidados, preceptora chega e fica escutando a passagem de plantão. Após enfermeira do noturno ir embora, R1 realiza a transferência de cuidados para preceptora. Esta, em alguns momentos, não entende determinadas propostas de pacientes, R1 consegue esclarecer. Exemplo: porque esta paciente não foi de alta? Ela já tinha leito! R1 responde que família não veio buscá-la, por isso não foi embora de alta.
				Organiza a escala de leitos com os TEs, vai perguntando para cada TE os leitos que estão assumindo, R1 diz que tem 1 leito sem TE, então um TE explica a R1 como eles se organizam e que na verdade todos os leitos estão cobertos. R1 atenta a explicação, apaga tudo que havia escrito e refaz a escala conforme explicação do TE. Durante a explicação do TE, R1 eleva sobrancelhas e balança cabeça concordando com a fala dele. Em seguida, inicia teste do desfibrilador, laringo e checa medicações multidoses.

					Checa caixa de curativos e faz levantamento das coberturas que necessitam ser solicitadas para utilização no dia. Em alguns momentos se demonstra insegura em relação a quantidade de determinada cobertura a solicitar, então confirma com preceptora se tal quantidade é adequada para o dia. Inicia solicitação das coberturas e insumos no sistema de prescrição eletrônica, ao solicitar gaze aberta estéril, R1 esquece como o nome deste insumo está descrito no sistema eletrônico, então pergunta para um TE que está no computador ao lado que fala alguns sinônimos para a gaze. R1 comenta que não tem descrito nenhum daqueles citados e continua a ler os nomes no sistema, à medida que lê vai dizendo "não é esse", em seguida diz "deve ser esse aqui". Finaliza as solicitações, imprime, assina e solicita TE para buscar. Ao chegar os insumos no setor, R1 abre o saco e rapidamente confere se a gaze que solicitou é a que realmente precisa, R1 fica feliz e conta para as pessoas ao redor que acertou o nome da gaze.
					Telefone toca, R1 atende, enfermeira do PA perguntando se pode subir com o paciente, R1 solicita aguardar um minuto na linha, então pergunta ao TE responsável pelo leito se está sabendo do caso e se o leito está pronto. TE diz não saber de admissão e que o leito está disponível. Em seguida vai a preceptora e pergunta se sabe sobre a admissão, responde que sim, mas não tem o caso do paciente. Então R1 retorna ao telefone, pega o caso do paciente e autoriza receber o paciente. R1 desliga o telefone e retorna ao TE para passar o caso para ele.
					Atende o telefone, perguntam se paciente x está de jejum, R1 fica uns instantes em silêncio, franze a testa e coça a sobrancelha. Então, solicita um minuto na linha e vai ao paciente e pergunta a ele se está de jejum. Paciente confirma o jejum e R1 confirma no telefone. Finalizada a ligação, R1 pega na mesa da supervisão o impresso de solicitação de exame e preenche. Preceptora chega à sala e R1 a pergunta se o paciente x fará ultrassom, preceptora diz que sim. R1 comunica ao TE que paciente irá fazer ultrassom, que por sua vez pergunta a R1 se é necessário levar o prontuário para o exame. R1 diz que não sabe e sai para perguntar a preceptora que explica o motivo de levar o prontuário na realização do exame. Ao final confirma com TE a necessidade de levar o prontuário, explicando ao TE o motivo.
					TE chega a R1 dizendo que paciente tem apenas HP e que as medicações prescritas são para administração endovenosa. R1 pega prescrição e solicita ao médico alterar as medicações da prescrição para infusão em HP. Ao ler prescrição, sai e pergunta a preceptora se o referido paciente pode ficar com apenas 1 HP, pois está recebendo medicações x, y e z. Preceptora explica as interações medicamentosas para R1 e em seguida R1 diz, então vai precisar puncionar mais um HP. Assim, retorna ao TE e explica a importância de puncionar novo HP.
					Preceptora pergunta a R1 se o paciente que irão admitir tem o perfil de idoso frágil a ponto de justificar admiti-lo nos leitos de idoso frágil, R1 para e pensa por uns instantes e responde que sim, em seguida descreve o perfil do paciente de forma a justificar seu perfil como idoso frágil.
					TE pergunta a R1 se realmente vai usar PVPI na ferida do paciente, então R1 vai ao paciente, avalia a ferida, diz "uhm", em seguida solicita TE para manter a conduta, explica que é necessário usar o PVPI para secar a ferida e orienta como deve ser feito o curativo.
					Avalia paciente 1: conversa com paciente, avalia e ao final diz ao paciente o quanto ele melhorou, sai feliz do leito.
					Avalia paciente 2: conversa com paciente à medida que palpa o abdome, tenta descobrir onde o paciente está sentindo dor. Avalia seus pés, diz ao paciente que está com edema nas pernas, palpa panturrilha, pergunta se tem dor. Comenta que os dedos dos pés estão muito frios, pergunta ao paciente se ele sente os dedos dos pés, faz teste de sensibilidade. Em seguida pergunta sobre alimentação, fezes, LPP. Paciente conta a R1 sobre possível cirurgia abdominal e demais problemas de saúde e familiar. R1 permanece em pé, escuta paciente.

				3° paciente: separa materiais para fazer curativo, chega no paciente e no acompanhante e diz que irá trocar os curativos. Pergunta ao TE responsável se precisa fazer medicação de resgate para dor, TE diz não estar prescrito. R1 não diz mais nada, solicita acompanhante a ajudá-la a lateralizar a paciente para iniciar os curativos. Abre os materiais e enquanto retira o curativo da pele da paciente, diz: "parece estar menos secretiva, está com menos gazinha", ao finalizar a retirada do curativo fala: " a lesão melhorou do último dia que eu fiz o curativo". Lava a ferida e diz "a lesão não está mais secretiva". Realiza o curativo em silêncio e concentrada. Ao final diz: "que felicidade". Cobre ferida com alginato de prata e gaze estéril. R1 não demonstra dificuldade ou insegurança para executar a atividade. Diz a acompanhante "ela está sentindo menos dor também, né?!". Enquanto identifica curativo e despreza materiais no lixo e organiza para trocar o próximo curativo da paciente, pergunta a acompanhante se paciente passou bem a noite, se alimentou, evacuou, etc. Realiza o segundo curativo, também elogia a progressão da ferida. Na terceira LPP, antes de retirar o curativo diz a acompanhante "essa tem mais gaze", acompanhante pergunta "R1, essa tem mais secreção, né?!" R1, "sim, precisa lavar bem a lesão antes de fazer esse curativo", R1 começa a explicar os motivos que a acompanhante precisa dar mais atenção a referida ferida. Ao cobrir a LPP, gaze não fica bem adaptada na ferida, vai mudando a posição da gaze até conseguir cobrir toda a lesão. R1 e acompanhante tiram fotos das 3 LPP. R1 ao finalizar todos os curativos diz: "bons tempos em paciente x, antes a senhora não ficava quieta durante o curativo e ainda sentia muita dor! Muito bom".
				4º paciente: Separa novos materiais de curativo e ao chegar no leito do paciente, percebe que o pacote de gaze estéril que havia deixado separado para o paciente foi usada. Fica com cara de incomodada, chega para os TEs e pergunta quem pegou o pacote de gaze que ela havia deixado separado. TE diz ser ele e pede desculpas. Vai para o computador, faz nova solicitação, imprime e vai a farmácia buscar novo pacote. Ao retornar, encontra com a preceptora e comenta sobre a evolução das feridas da paciente 3, mostra fotos e diz estar feliz com a evolução. Preceptora fala que no dia seguinte elas farão a prescrição de alta da paciente e que vão prescrever alginato de prata, R1 pergunta se a instituição vai fornecer as placas de alginato para paciente levar para casa. Preceptora diz que não e explica os motivos. Preceptora fala sobre o curativo que acabou de fazer no paciente y, explica detalhes dos passos da troca do curativo. Diz que aspirou com agulha de insulina os flictemas e conta o que espera após o procedimento que executou. Mostra foto para R1 do antes e depois do procedimento. R1 atenta a fala da preceptora, expressa sua empolgação e diz "eu queria ter visto".
				R1 vai para o 4º paciente realizar uma nova troca de curativo. Explica ao paciente e acompanhante que irá trocar o curativo. Abre e higieniza a ferida de erisipela. Acompanhante diz: "as bolhas romperam", R1: "isso era o esperado", em seguida começa a explicar para a acompanhante como é o tratamento destes tipos de feridas. Durante a conversa, TE que está dando banho no paciente ao lado chama R1 para avaliar lesão, R1 abre a cortina, olha e avalia a ferida do paciente que se encontra no banho. Orienta TE a utilizar petrolato na ferida. Retorna para a troca do curativo do paciente 4, pega uma gaze e fica apertando a gaze sobre a ferida para drenar. R1 chama a preceptora e mostra a lesão para ela, pergunta se precisa drenar mais. Preceptora pergunta o aspecto da secreção e R1 responde. Preceptora vai explicando o que R1 precisa fazer e R1 vai executando, enquanto isso acompanhante fica olhando. Em alguns momentos, R1 pergunta se o paciente sente dor. Acompanhante pergunta a R1 se está melhorando, R1 diz que sim e explica a acompanhante o tipo de cicatrização que aquela ferida está seguindo. Preceptora sai do leito para atender telefone. R1 para por uns segundos, olha para a ferida e procura a preceptora com o olhar, achando que ela estava dentro da enfermaria. Então R1 chega ao corredor e chama a preceptora. Neste momento R1 demonstra insegurança na continuidade do curativo. Preceptora chega e R1 pergunta o que ela acha que deve usar de cobertura em cada local da ferida. Preceptora explica e sai novamente do leito. Assim R1 começa a cobrir a ferida com a cobertura que preceptora orientou, utiliza placa de petrolato e depois diz "e agora?" (percebo R1 com dificuldade técnica para fazer o curativo). Logo em seguida, pega nova placa de petrolato, corta e distribui em todos os pontos da lesão. Ao final diz ao paciente: "sua ferida melhorou muito, estamos usando pouco petrolato agora". Coloca uma atadura para cobrir

				a lesão, quando finaliza vê que não ficou bom e coloca uma segunda atadura. Finaliza o curativo, identifica, guarda materiais e despreza outros materiais.
				Acompanhante chama R1 e mostra edema na perna do paciente onde tem um HP. R1 avalia, pergunta ao paciente se sente dor no local e explica a acompanhante que o acesso não está bom. Diz que vai puncionar um novo. Vai ao posto de enfermagem, separa material, ao abrir os materiais a beira leito, percebe que esqueceu o jelco, retorna ao posto e busca. Comunica paciente que irá puncionar outro HP, punciona e checa a permeabilidade. Liga soro no novo HP e retira HP antigo. Ao retirar o HP antigo, olha para o jelco e faz uma cara de assustada e mostra a acompanhante dizendo: "olha como o jelco está torto!"
				Avalia o 5º paciente, conversa com familiares e paciente. Pergunta sobre alimentação, fezes, urina, etc. Paciente começa a dizer frases desconexas e R1 percebe que paciente está confuso, faz perguntas para confirmar lucidez. Então volta sua conversa para os familiares. Explica aos familiares o motivo das fezes líquidas e da SVD.
				Avalia 6° paciente. Paciente fala sobre perna inchada, avalia, palpa e continua a fazer perguntas para o paciente. Movimenta suas pernas e pergunta sobre dor.
25/08/2022	13:00	19:30	Idoso Frágil e Paliativo	Transferência de cuidados realizada entre R0 (estava no turno da manhã) e R1 (chegando para trabalhar no turno tarde). R1 escuta e escreve pontos importantes. Alguns momentos faz perguntas para entender os detalhes dos casos dos pacientes como, horário que realizará raio-x, se psicologia está acompanhando, se dieta já foi liberada, etc. Ao final, R1 divide os pacientes entre ela e a preceptora.
				Prepara material para punção de HP, ao chegar no paciente percebe que esqueceu o jelco, então retorna para buscar. Comunica paciente sobre a punção de HP e pergunta o local que o paciente prefere a punção, perna ou abdômen, paciente diz não fazer diferença. R1 punciona em coxa direita, testa a permeabilidade do acesso, faz curativo e inicia infusão de SF a 40 ml/h.
				R0 passa pelo setor e ao conversar com R1 expressa suas inseguranças com o estudo de caso que irá apresentar na próxima 5ª feira. R1 escuta e ajuda R0, fornecendo dicas para estudar sobre o paciente. R1 pergunta qual é o paciente do estudo de caso e qual o diagnóstico. Então R1 e R0 leem no passômetro o caso da paciente. R1 fala sobre alguns cuidados de enfermagem como não pular a medicação de resgate para a dor do paciente, ainda comenta que alguns profissionais preferem antecipar o horário de medicações para dor do que fazer o resgate, salienta ser importante as outras residentes entenderem a importância do resgate (referencia sua fala com a palestra que participaram sobre controle da dor). Orienta levar fotos da LPP e levantar os motivos que a ferida não progrediu para cicatrização, além de dar ideia para elencar as dificuldades que o serviço social estava tendo para lhe dar com a família. Enquanto R1 fala, R0 concorda, anota e complementa a fala de R1 com outras ideias.
				Hundle (reunião das principais chefias da instituição como BC, CTIs, clínica médica, PA, que acontece 3 vezes ao dia no intuito de otimizar a gestão de leitos). R1 e preceptora caminham em direção ao PA para participar do Hundle, neste período preceptora explica o que é hundle e como funciona, à medida que preceptora explica, R1 permanece atenta com o olhar direcionado a preceptora e faz perguntas como: o que significa a palavra hundle e se estas reuniões resolvem o problema de leitos ociosos no hospital. Ao chegar no PA, preceptora entra na sala que se encontra cheia e é pequena. R1 fica parada na porta, encostada na parede escutando. O chefe da gestão de leitos vai perguntando o número de leitos vagos, óbitos, altas efetivadas e previstas e emergência social que pode ser resolvida de imediato, preceptora responde. Reunião dura 10 minutos, ao final elas retornam para o setor de origem. No caminho de retorno, R1 comenta que achou efetiva a conversa e rápida. Disse ainda, ter achado interessante, pois uma comunicação rápida, conseguiu resolver o problema de falta de leito na terapia intensiva.

				Retorna para o setor e inicia a avaliação de pacientes. 1º paciente: conversa com paciente e acompanhante, pergunta sobre alimentação, eliminação, conforto e sono.
				2º paciente: durante a avaliação paciente pergunta onde fica o jardim do hospital, R1 não sabe responder, paciente detalha mais como é o jardim. R1 diz a ele que ficou curiosa para saber onde fica este local e que vai procurar saber onde fica. Paciente reclama que não tem lugar para andar no hospital, R1 diz que vai tentar resolver. (R1 não procurou saber onde fica o jardim).
				Sai da enfermaria e inicia avaliação do 3º paciente, pergunta sobre alimentação, eliminação, dor, faz exame físico, olha as medicações que estão infundindo.
				TE chega e pergunta à preceptora se o ECG está bom, preceptora diz que sim, mas complementa que a amplitude está ruim. R1 que se encontra ao lado da preceptora pergunta o motivo das ondas QRS estarem espaçadas. Preceptora explica e ao final, R1 diz para a preceptora dar uma aula de ECG para as residentes, preceptora diz não ter domínio a ponto de dar uma aula.
				Médico entrega prescrição médica e explica a R1 sobre a gravidade do paciente. Explica os motivos que o paciente não tolera a implantação de Stent. R1 fica atenta a fala, eleva sobrancelhas e fica com cara de assustada à medida que o médico explica ao quão complexo é o caso do paciente. Ao final, R1 apraza prescrição médica e sai para entregar ao TE. Neste momento, explica a gravidade e complexidade clínica do paciente para a TE, conforme explicação médica, e ressalta a importância de iniciar as medicações do paciente naquele instante.
				Prepara material para SVA, durante a separação dos insumos, R1 diz pensando alto "acho que estou esquecendo de alguma coisa", então olha para os materiais e passa um a um dizendo os nomes na tentativa de lembrar o que falta. Nesse meio tempo, acompanhante chama R1 para ajudá-la pois a paciente está querendo descer da cama. Ajuda a acompanhante posicionar paciente no leito, na mesma hora paciente se movimenta e tenta novamente descer do leito. Acompanhante diz: "vai precisar conter desse jeito". R1 tenta segurar as mãos da paciente com o cobertor e depois sai da enfermaria, chega para a preceptora dizendo sobre a agitação da paciente. Preceptora e R1 chegam ao leito novamente e R1 diz: "acho que ela perdeu o AVP". Preceptora pega paciente e a coloca sentada a beira leito, R1 fica no pé da cama observando. Preceptora pede a R1 para buscar oxímetro, R1 busca e retorna colocando no dedo da paciente (Sat. O2 95%). Em seguida, R1 abre cortina e diz "só dela conseguir olhar um pouco para a janela já é melhor". Acompanhante diz que queria levá-la ao banheiro, então preceptora pede a R1 para buscar bala de oxigênio, preceptora e R1 conectam paciente na bala de oxigênio e acompanhante leva paciente ao banheiro e em seguida anda um pouco com a paciente pelo corredor.
				Volta a preparar materiais de SVA, orienta paciente e explica o procedimento. Avalia bexigoma, pergunta a paciente se sente dor quando aperta a região inferior do abdômen, paciente diz que não. Pergunta se ela está com vontade de urinar e qual foi a última vez que passaram SVA nela, não sabe responder. Abre materiais e organiza mesa de procedimento. Acompanhante pergunta o motivo da passagem de SVA, R1 explica que precisa coletar amostra de urina para cultura. Acompanhante sai do leito e R1 inicia o procedimento. Paciente tímida, não deixa R1 fazer higienização, R1 pacientemente explica da importância e com jeitinho convence a paciente. Durante a passagem da SVA, R1 abre os grandes lábios e fica parada por uns instantes procurando a uretra, encontra e passa SVA. Durante o procedimento paciente não colabora muito, mas R1 segura do procedimento, soube conduzir a paciente, R1 começa a falar mais firme e não dá muitas alternativas para a paciente. Despreza lixo, diurese, organiza o leito e a paciente, ao final identifica o frasco de diurese. Ao encontrar com a preceptora no café fala sobre suas dificuldades durante a passagem de CVA, fala que a paciente não colaborou e com isso teve dificuldade para visualizar o canal da uretra, mas que ao final deu tudo certo.

				TE chama R1 e avisa que paciente está com epistaxe peri CNE, R1 antes de avaliar já solicita preceptora para ir com ela no paciente. Chegam no paciente, preceptora avalia e movimenta a sonda, R1 observa com cara de assustada e em um determinado momento agacha para conseguir visualizar melhor o sangramento. Preceptora comenta as possibilidades de causa daquele sangramento e diz que não precisam se preocupar, apenas continuar observando. Saem do leito. R1 comenta com preceptora que nunca tinha visto uma epistaxe por causa de CNE.
				Inicia as evoluções no sistema eletrônico. R1 mantém-se concentrada, olha suas anotações em passômetro e digita no computador. Recebe diversas interrupções, mas não dá atenção a elas. Preceptora avisa a R1 que paciente que acabou de chegar da admissão, faleceu. R1 faz uma expressão de assustada e volta a evoluir. Pergunta para a preceptora que está evoluindo ao seu lado se paciente x está com dreno de tórax, preceptora não escuta, R1 volta a evoluir. Depois de um tempo movimenta na cadeira e diz em voz alta " ahh está sim! Vi ele no corredor segurando o dreno".
				Preceptora solicita R1 avaliar paciente x, R1 chega, observa paciente e tenta conversar com ela e não consegue (paciente sonolenta e com esforço respiratório). TE que está no leito também está avaliando paciente e checando os sinais vitais. R1 sai para chamar o médico e ao encontrar, fala que paciente está sonolenta. TE chega ao médico durante a fala de R1 (TE corta a fala de R1) dizendo os sinais vitais da paciente. R1 ignora a situação e sai para conferir as caixas de curativo. TE chega a R1 e a orienta (TE orienta com uma fala mais nervosa e sem paciência) sobre a importância de levar os dados vitais ao médico ao comunicar uma situação mais grave, diz que antes de comunicar é necessário avaliar, fazer exame físico do paciente e verificar se não há nada de errado antes de sair chamando o médico. R1 concorda com a fala da TE, balançando a cabeça, percebo que R1 ficou sem graça com a situação e continua mexendo nas caixas de curativo. TE sai.
				R1 admite paciente, realiza exame físico rápido, checa vazão de O2 e escuta o TE falar sobre o que já avaliou do paciente. Ao final, vai para o computador e faz sua evolução.
				R1 encontra com preceptora no posto de enfermagem e percebe que ela está resolvendo as questões administrativas do óbito. R1 pergunta como funciona o fluxo de óbito e os documentos necessários para fechamento do óbito. Preceptora à medida que vai andando, vai explicando a R1 todas as etapas. R1 caminha atrás da preceptora atenta a explicação, em alguns momentos balança a cabeça no sentido de estar concordando. Ao final, R1 diz que no próximo óbito quer fazer todo este processo sozinho para ver se aprendeu.
				Ao final da explicação, retorna a evoluir, TE chega e solicita atadura, sai para buscar atadura junto ao TE, neste momento, médico chega para pegar a atadura com o TE e os 2 saem. R1 pergunta pensando alto "o que está acontecendo gente?", ninguém responde e ela retorna a evoluir.
				Transferência de cuidados entre preceptora e enfermeiro noturno. R1 troca de roupa, guarda pertences pessoais, vai ao banheiro, ao finalizar, fica encostada na parede escutando. Nos momentos que está na sala, complementa alguns casos de pacientes com informações adicionais.
01/09/2022	14:00	16:30	Estudo de Caso Auditório	**1ª Apresentação de Estudo de Caso: manejo da dor. Dupla: Residente X e R1.** Residente X inicia a apresentação do caso clínico falando sobre a fisiologia da dor, enquanto isso R1 fica sentada na primeira fileira do auditório logo a frente da residente x. Durante a apresentação, R1 permanece sentada com os dois pés no chão (sentada na beirada da poltrona e sem encostar suas costas) e com o tronco inclinado para frente. Durante toda a apresentação da residente x (50 minutos), R1 fica atenta a sua fala, em alguns momentos balança a cabeça no sentido de estar concordando com a fala. Olha para a residente x e para os slides durante toda a apresentação. Enfermeira do cuidado paliativo interrompe a apresentação e associa o que a

				residente x está falando sobre dor com o caso de uma paciente que está internada no 6º andar ala A. R1 movimenta o seu corpo para conseguir olhar para a enfermeira falando, R1 identifica qual é a paciente que a enfermeira está exemplificando e fala o nome da paciente em voz baixa, ao final da fala da enfermeira, R1 dá uma risadinha e diz "é assim mesmo" (no sentido de estar concordando com a fala). Durante a apresentação, residente x faz uma indagação e continua sua fala, neste instante R1 responde a pergunta realizada em voz baixa. Residente x ao responder sua indagação feita durante a apresentação, R1 movimenta a cabeça no sentido de concordar com a resposta. Residente x ao apresentar esquece o nome do aparelho que dá choque e de forma imediata R1 a ajuda dizendo "desfibrilador".
				Término da apresentação da residente x, R1 caminha em direção ao quadro e dá continuidade a apresentação do caso clínico. A apresentação da R1 é voltada para o caso de uma paciente que permaneceu internada no 6º andar ala A e R1 identificou dificuldade de a equipe realizar o manejo da dor. Durante a sua apresentação R1 passa segurança em sua fala e domínio do assunto. Apresenta um protocolo construído pela instituição para os residentes de medicina realizar o manejo da dor nas enfermarias. Neste momento em que R1 apresenta o protocolo, em várias oportunidades ela olha para a enfermeira da clínica da dor, esperando que a mesma faça algum comentário. No entanto, nada acontece. Em um determinado ponto de sua apresentação, R1 começa a dar exemplos de medicações presentes nas prescrições médicas que tem a finalidade de aliviar a dor e as relacionam com o conhecimento teórico apresentado pela residente x. R1 ao final da apresentação faz uma crítica dizendo que infelizmente a equipe multiprofissional ainda não está preparada para tratar a dor de seus pacientes, fala um pouco dos métodos não farmacológicos, expõe algumas situações e algumas formas que podem melhorar o processo.
				R1 finaliza a apresentação, então a coordenadora do programa diz que sentiu falta da residente x e R1 abordarem a SAE no estudo de caso, R1 neste momento diz "nossa!! É mesmo...". Então coordenadora realiza um intervalo de 15 minutos. No intervalo, todas as residentes se reúnem e ficam conversando dentro do auditório. Iniciam uma conversa sobre a LPP do caso clínico apresentado no slide da residente x e R1 com a LPP de uma outra paciente. R1 fala que as lesões são parecidas e compara o tratamento entre as duas lesões, ao final diz que não entende o motivo de uma lesão ter fechado e outra não. Então, residente y diz que a condição clínica entre as duas pacientes era muito diferente e que isso deve ter interferido no tratamento. R1 explica com detalhe como realizava o curativo da paciente do caso clínico.
				**2ª Apresentação de Estudo de Caso: Status epilepticus. Dupla: Residente Y e R2.** R1 se senta próximo ao computador e fica passando os slides da apresentação. Durante a fala das colegas em um momento inclina muito o corpo no sentido de melhorar sua visualização de um desenho que está sendo apresentado e explicado no slide. R1 olha com atenção para o slide e para a colega que está falando.

Fonte: Elaborado pelas autoras.

O quadro 2 apresenta as descrições das atividades desenvolvidas pelo Residente 2 (R2) do segundo ano do PRAPS Modalidade Uniprofissional Intensivismo, Urgência e Trauma em 33 horas de observação.

Quadro 2 - Descrição das atividades desenvolvidas pelo R2 em 33 horas de observação.

Data Observação	Horário Início	Horário Término	Setor Observação	Descrição das atividades realizadas pelos residentes

02/08/2022	10:10	12:00	Semi-intensivo	R2 evolui os seus pacientes no sistema eletrônico. Durante toda a evolução, R2 mantém-se concentrada olhando para o computador e digitando, em alguns momentos lê suas anotações no passômetro e retorna para a evolução.
				R2 prescreve os materiais que irá utilizar para o curativo, vai a farmácia buscar o material e em seguida caminha para o leito do paciente. Antes de iniciar o curativo, R2 explica o procedimento ao paciente e inicia o curativo. Enquanto R2 realiza a higienização do coto, o paciente diz a ela para utilizar hidrogel com petrolato na ferida, R2 diz que irá avaliar a ferida e definir a conduta, mas já antecipa que as duas coberturas não podem ser utilizadas em um mesmo local. Explica a ferida da amputação para o paciente, detalha os locais onde colocará hidrogel e onde colocará petrolato. Neste momento, a preceptora chega e fica observando. O paciente pergunta a R2 se aquele hidrogel é puro ou se está associado à colagenase, R2 fica alguns instantes em silêncio e em seguida lê o rótulo do produto e responde que é puro. Preceptora fornece materiais para a R2 fazer o curativo e continua observando, em alguns momentos realiza comentários sobre a evolução da ferida. Ao final, R2 pergunta ao paciente se o curativo está confortável ou apertando o coto, ele responde que está bom. R2 reúne os resíduos e despreza na lixeira. Ao realizar a higienização das mãos, R2 lembra que não identificou o curativo, então retorna e identifica. Em seguida checa prescrição e guarda no prontuário do paciente.
				Antes de iniciar a evolução do curativo, TE pede a R2 para solicitar no sistema materiais para punção de acesso periférico, na ocasião R2 pergunta se o acesso tem retorno e TE diz que o acesso está ruim.
				Inicia evolução da troca de curativo, após uns minutos R2 vai ao paciente e pergunta quando aquela ferida no coto abriu, paciente não sabe responder, assim R2 retorna para o computador, procura e lê evoluções médicas sobre a história do paciente. Preceptora chega à sala e R2 pergunta se ela sabe quando a ferida do paciente abriu, preceptora diz não saber e orienta R2 a ler a evolução médica e a de enfermagem de admissão do paciente. Retorna para a sua evolução de troca de curativo e para as leituras das evoluções dos profissionais sobre a história do paciente.
				Durante a leitura das evoluções, TE chega e comunica leito de alta do paciente y. Assim, R2 liga na enfermaria e realiza tentativa de transferência de cuidados do paciente de alta, no entanto enfermeira nega a pegar o caso naquele momento. R2 pergunta a enfermeira do andar o tempo para o leito ser liberado, o horário que pode ligar novamente para realizar a transferência de cuidados e quais são as pendências para que o leito ocupado seja liberado. Desliga o telefone e retorna para a leitura das evoluções. TE chega à sala de evolução e R2 comunica que o leito ainda não está disponível para levar o paciente de alta, explica os motivos para a TE.
				Preenche passômetro de enfermagem para realizar a transferência de cuidados para a enfermeira da tarde.
30/08/2022	13:10	19:40	Semi-intensivo	R2 prepara material para passar CVA em paciente que se encontra em protocolo, chega ao paciente e explica procedimento, abre e organiza materiais na mesa de procedimento. Retira fralda da paciente e higieniza, à medida que vai passando a sonda, R2 vai conversando com paciente sobre assuntos aleatórios. Passagem de sonda sem dificuldade, R2 transparece segura e confiante nos passos da técnica. Recolhe os materiais, despreza resíduos e quantifica volume urinário. Fala com paciente o volume que foi drenado e sai do leito.

					R2 percebe movimento em enfermaria ao lado e vai olhar o que está acontecendo. Ao chegar visualiza a equipe organizando materiais para uma troca de cânula de TQT. Presença de médico, fisioterapeuta, enfermeira preceptora e TE. R2 começa a auxiliar a equipe, abre os materiais e ajuda a posicionar paciente. Demonstra ter conhecimento das etapas do procedimento. Durante o procedimento houve necessidade de pegar um fio de sutura, R2 sai para buscar, no meio do caminho volta e pergunta para a preceptora se o fio é pego na farmácia mesmo, preceptora concorda. Término do procedimento. R2 durante todo o procedimento fica mais de expectadora, mas durante todo o tempo atenta as necessidades da equipe e a qualquer comentário que havia sobre necessidades, já saía para resolver. Além disso, ficou atenta aos passos que a preceptora desenvolvia durante o procedimento, ficou acompanhando preceptora com o olhar; aproximou-se dela durante a administração de sedação e chegou a perguntar a preceptora se o acesso já havia sido testado antes de iniciar a administração dos sedativos.
					Término da troca de cânula de TQT, R2 inicia sua evolução de passagem de CVA, ao final procura prescrição médica para checar o procedimento, mas não encontra o procedimento prescrito. Então diz a preceptora, que orienta solicitar ao plantonista a prescrição.
					TE solicita a ajuda de R2 para mudar seus pacientes de decúbito, as duas reposicionam 4 pacientes. R2 auxilia a TE sem dificuldades. R2 com boa performance ao manipular os pacientes.
					Preceptora pergunta a R2 onde está o monitor de transporte, R2 diz que não sabe e sai pelas enfermarias procurando. Pergunta aos TEs, que também não sabem. Então começa a checar os monitores que estão conectados nos pacientes e o encontra monitorizando o paciente x. Chega à preceptora e fala onde está o monitor, em seguida retorna para o posto de enfermagem. Plantão muito tranquilo, com 9 pacientes de alta, então fica muito tempo sentada no posto de enfermagem utilizando celular ou conversando assuntos aleatórios com a equipe de enfermagem.
					NIR liga perguntando se tem leito disponível, R2 diz que não e desliga o telefone. Em seguida abre o passômetro e conta o número de pacientes de alta, faz o levantamento de quantos pacientes tem leito e quantos não tem e diz: "não vai ter leito não". Preceptora chega no posto de enfermagem e R2 diz a ela, "o NIR solicitou vaga para admissão, mas estamos com 9 pacientes de alta e todos sem leito". Preceptora diz: "tem que esperar sair leito". Preceptora começa a dizer que os médicos esquecem de passar nas enfermarias dando alta para os pacientes, então o setor semi-intensivo fica com esse tanto de paciente de alta sem leito nas enfermarias. R2 diz "Vou escrever neste papel aqui todos os pacientes de alta, assim que o NIR começar a ligar nos falando as altas, vamos escrevendo aqui para os TEs verem e ir liberando os pacientes".
					R2 comenta com a preceptora sobre a apresentação de um caso clínico que fará nas próximas semanas e a convida para assistir. Começam a conversar sobre o caso clínico, R2 conta que será sobre um paciente que chegou na UPA convulsionando e que mesmo administrando diazepan e dormonid, o paciente não parava de convulsionar. Então fala que na sua apresentação irá focar nos cuidados de enfermagem e falar sobre as medicações que normalmente são utilizadas no tratamento. Em seguida, preceptora conta um caso que vivenciou de uma paciente que passou toda a manhã convulsionando. R2 pergunta as medicações que utilizaram na época e como ela conduziu o caso. Preceptora conta com detalhes sua experiência e falam sobre a fisiologia da convulsão.
					Enfermeira entrega a R2 folha da CCIH que acabou de imprimir e solicita colocar no painel do posto de enfermagem. R2 pega folha e lê, franze a testa e pergunta a função daquele impresso. Enfermeira diz ser o impresso de controle de precauções de contato dos pacientes internados na unidade, explica que aquela folha deve ser impressa diariamente e colocada no quadro do posto de enfermagem. Assim, a equipe tem o controle dos pacientes em precaução, após a fala da enfermeira, R2 diz "ahhhh... entendi" e balança a cabeça no sentido de estar concordando. R2 coloca folha no painel e fica anotando em seu passômetro os pacientes que estão em precaução.

				Senta-se no computador e lê os resultados das gasometrias dos pacientes que está sob sua responsabilidade. Alguns resultados alterados faz cara de espanto e registra resultados alterados no passômetro. Não tomou nenhuma iniciativa ou comunicou sobre alterações.
				R2 atende o telefone e recebe leito de alta do paciente x, comunica a TE. Abre caderno de altas da unidade e começa a anotar os horários que recebeu o número do leito e que comunicou ao TE.
				Médico chega e pergunta o volume que foi drenado no CVA, R2 responde e solicita prescrição do procedimento. Médico diz que irá prescrever e já pergunta o número da sonda que utilizou. R2 recebe prescrição, checa e grampeia com a prescrição médica do paciente.
				Recebe mais um leito de alta, comunica ao TE e depois faz anotações no caderno de alta. Preceptora chega e R2 pergunta o conceito de "hora de alta NIR" e já fala o seu entendimento. Preceptora então, explica o conceito correto e R2 diz que sempre escreveu o horário errado no caderno "Nossa... eu sempre fiz errado". Pega borracha e apaga os horários que havia escrito e coloca o correto. Plantão muito tranquilo, ficam muito tempo no posto de enfermagem conversando e rindo.
				R2 recebe outro leito de alta e comunica ao TE. Enfermeira escuta e comenta que paciente é muito sofrido, que utiliza CVD desde dezembro de 2021 e que a sonda vai sendo apenas trocada para evitar infecção. Preceptora complementa que paciente tem uma LPP importante na região sacral por ser cadeirante e diz que o tratamento da lesão já até levou a confecção de colostomia, mas que a ferida não fecha. Neste instante R2 que estava escutando a fala da preceptora e da enfermeira diz: "nossa!!! é por isso então que ele tem colostomia? Eu não sabia disso!!" R2 fala com cara de surpresa.
				Enfermeira comenta sobre o post de WhatsApp que uma colega de trabalho colocou no grupo e a repercussão que causou. Diz que o post causou uma discussão bacana e que não sabia que diferentes concentrações de citrato influenciava os exames de coagulação. (preceptora também está no grupo do WhatsApp e entendeu a conversa). Preceptora e enfermeira começam a conversar sobre os motivos de utilização do citrato e R2 tenta acompanhar a conversa, mas não consegue. Ao final R2 pede a preceptora para enviar o post para ela ler e diz "eu não entendo nada disso".
				Preceptora explica para R2 seu planejamento de capacitação sobre administração de medicamentos que dará para a equipe de TEs. R2 vai concordando com a fala, balançando a cabeça e dizendo "aham". R2 opina dizendo para a preceptora fazer um treinamento mais rápido e mais prático, pois ela acredita que as pessoas aprestam mais atenção. R2 conta sobre um treinamento de administração de medicamentos que participou, descreve detalhes da metodologia da capacitação, o que aprendeu e elogia o treinamento, ao final diz "quem sabe não faz um parecido?" Preceptora concorda e diz que vai chamá-la para ajudar no treinamento.
				R2 atende o telefone e recebe 2 casos de pacientes de admissão. Durante a transferência de cuidados, R2 pergunta o volume drenado pelo dreno de tórax, em seguida diz "nossa!!!", fala com voz assustada. Desliga o telefone e passa com detalhe os 2 casos para a enfermeira e a preceptora, fala que o dreno de tórax já drenou 700 ml de sangue e diz "muito, né?", enfermeira e preceptora apenas concordam. Ao final, vai nos 2 leitos vagos e olha se estão limpos e montados e em seguida passa os casos para os TEs.

				TE chega ao posto de enfermagem e diz que paciente x perdeu o AVP. Preceptora e R2 vão para o leito e começam a avaliar MMSS procurando veias, não encontram. Preceptora pergunta a R2 se ela deseja tentar puncionar veia jugular, R2 diz sim. Então preceptora posiciona paciente e explica o procedimento a ela. Enquanto isso, R2 prende o cabelo e se posiciona na cabeceira da cama. R2 avalia a região, preceptora segura a pele do pescoço do paciente para ajudar na visualização da veia, R2 posiciona o jelco para iniciar a punção e diz que a cabeceira da cama está atrapalhando. Retira a cabeceira e volta a posicionar o jelco. Paciente movimenta um pouco o pescoço e R2 diz "paciente acordada é ...". Antes de puncionar, fica procurando a veia e avaliando o ponto onde irá inserir o jelco, em seguida punciona veia, ao checar retorno começa a voltar sangue, mas depois para de ter retorno. Preceptora diz para puncionar um pouco mais embaixo, mas R2 diz que não gosta de puncionar mais para baixo pois o jelco fica dobrando. R2 pega seringa para checar retorno e pergunta se a preceptora já desceu o soro para testar retorno, preceptora diz que sim. Então R2 retira o jelco e diz que não puncionou. Começam a avaliar novamente o pescoço da paciente e preceptora diz "será que a veia estourou?" R2 diz "talvez puncionar mais em baixo como você disse, deve dar certo".
				Preceptora inicia a punção, R2 observa com atenção, acompanha com o olhar e ajuda a preceptora segurando a pele da paciente, preceptora também não consegue. Preceptora solicita ajuda a enfermeira que inicia tentativa na outra veia jugular, R2 pergunta se quer que clareie e ligue a lanterna do celular. R2 também fornece os materiais de punção para ela. R2 continua atenta a punção. Enfermeira punciona rápido e R2 diz "o acesso está melhor que um central". Enfermeira sai e R2 agradece, faz o curativo e ao finalizar, olha para o curativo e diz "o curativo vai soltar", então pega outro filme transparente e reforça o curativo. Preceptora diz a R2 que o acesso que tentaram pegar deve ter transfixado, R2 diz "ahhh.. sim... pois no início quando puncionei, tinha retorno". R2 monitoriza paciente, retorna com a cabeceira da cama, eleva grades, arruma paciente no leito e sai do leito. Vai para o computador evoluir a punção.
				Final do plantão mais agitado, apressam as atividades para receber a admissão. Impressora estraga o que gera um estresse na equipe. Admissão chega às 18:50, R2, enfermeira e 2 TEs recebem paciente, organizam paciente no leito, trocam fralda e roupa de cama. R2 monitoriza paciente e se apresenta ao paciente. Em seguida pega os frascos de swab, identifica e coleta swab nasal e perianal. Médico chega e avalia o paciente, não fala nada com a equipe. R2 organiza soro e liga na BI. Preceptora inicia a realização de ECG, R2 diz para a preceptora que será difícil fazer o ECG, pois paciente não para de movimentar. Então, enquanto preceptora segura os MMSS do paciente, ela vai orientando R2 a como mexer no aparelho de ECG. Ao finalizar o ECG, faz curativo de CVP. Preceptora diz que parece que paciente tem problema de vista, R2 pergunta ao paciente que não responde, então R2 se aproxima ao paciente e começa a fazer uns testes para verificar a visão.
				R2 atende o telefone e recebe a notícia que um TE faltará no plantão noturno. R2 pega a escala e já fala com a preceptora que vai faltar um TE no plantão. Preceptora diz que irá evoluir a admissão e que é para ela reorganizar a escala de leito do plantão noturno. R2 se sentou no posto de enfermagem e começou a avaliar escala. R2 monta uma escala, mas ao finalizar, olha de novo para a escala e apaga tudo que fez. Enfermeira passa e pergunta porque apagou tudo, R2 diz que da forma como havia feito ficaria pesado para os TE x e Y. Enfermeira dá uma ideia para R2 e ela diz "é mesmo, fica bom assim". R2 constrói uma nova escala e sai avisando aos TE do noturno que já estavam pegando plantão.
				Retorna ao posto de enfermagem, finaliza suas evoluções e faz diagnóstico e prescrição de enfermagem do paciente admitido. Vai ao leito de admissão e identifica o leito com uma placa de identificação. Ao final, diz a preceptora que já está indo embora e despede da equipe.

31/08/2022	14:00	15:30	Auditório	Residente Z iniciou a apresentação sobre Suporte Básico de Vida, coordenadora do programa ficou passando os slides da apresentação e R2 ficou sentada na terceira fileira do auditório, com a perna cruzada, mão no rosto, cotovelos sobre a poltrona e encostada na poltrona. Ficou com o olhar direcionado para a residente z durante a apresentação e alguns momentos utilizava o celular. No decorrer da apresentação, R2 tira sua mão do rosto e coloca na cabeça, deixando sua cabeça mais deitada possível sobre a mão (aparência de estar com sono). Em um momento, utilizou o celular e mostrou uma imagem para a colega que estava sentada ao lado. Não realiza anotações. Bem no final da apresentação da residente z, R2 interrompe a fala da mesma e corrige uma fala da residente Z. Diz "na verdade, não se utiliza nenhuma sedação no pós-PCR imediato, pois é importante avaliar o Glasgow deste paciente". Além desta fala, R2 aproveita e complementa que na prescrição de enfermagem deste paciente também é importante prescrever cuidados relacionados a hipotermia terapêutica.
				Residente Z finaliza sua apresentação e R2 caminha em direção ao quadro para dar continuidade ao assunto. A apresentação é guiada pelos slides, lê o que está escrito nos slides, mas depois explica e exemplifica com situações do dia a dia das residentes. Além disso, relaciona os materiais ilustrados nos slides que são utilizados durante uma parada cardiorrespiratória com os materiais dos setores em que estão na prática. R2 passa segurança em sua fala. Realiza uma apresentação rápida e objetiva. Durante sua apresentação, R2 ao falar sobre a qualidade das compressões referencia sua fala com a fala da residente z. "Como disse a residente z, as compressões para ser de qualidade ...".
05/09/2022	13:00	19:20	Semi-intensivo	R2 pega os casos dos pacientes junto a preceptora, no período da manhã R2 estava no CTI2, no período da tarde vem para o semi-intensivo para acompanhar a sua preceptora que está escalada para o semi-intensivo. R2 senta o lado da preceptora e de frente a enfermeira da manhã. Durante toda a transferência de cuidados R2 faz movimentos com a cabeça, no sentido de estar concordando com a fala e em outros momentos diz "aham", faz anotações das propostas e pontos relevantes para o plantão. Faz algumas perguntas relacionadas aos pacientes como "qual a vazão da nora?"; "vai puncionar CDL?". Enfermeira que está fazendo a transferência de cuidados responde R2. Ao final, R2 diz a preceptora que irá almoçar rapidinho e retorna para realizar a tomografia do paciente x.
				Após 30 minutos, R2 retorna para a unidade e começa a preparar paciente para realizar tomografia. Pega maleta de transporte, bala de oxigênio, monitor multiparâmetro e suporte de soro e coloca na cama. Retira as bombas de infusão dos suportes e passa para o suporte de soro acoplado na cama. Explica ao paciente que será levado para realizar tomografia. Em seguida, chama o TE para fazer o duplo cheque dos materiais necessários para o transporte e confirma que está completo. Chama o residente de medicina para acompanhá-los no transporte. Transporte realizado pelo R2, residente de medicina e TE. Durante o deslocamento foram conversando sobre assuntos aleatórios, ao chegar na sala, passam paciente para a mesa do tomógrafo e vão para a sala onde ficam vendo o paciente e o monitor. Ao finalizar o exame, retornam para o semi-intensivo e organizam paciente no leito.
				R2 se senta para evoluir, equipe de TE conversando ao seu redor, mas mantém-se concentrada em sua evolução. Preceptora chega e se senta no computador ao lado, comenta com R2 que ainda está selecionando as medicações que incluirá no treinamento para a equipe de enfermagem. R2 fala de algumas medicações que acha importante incluir e diz "são medicações que direto geram discussões aqui".
				Ao finalizar sua evolução, R2 solicita a preceptora para ler uma parte da sua evolução e pergunta se normalmente é assim que evolui um paciente com gota. Preceptora lê e diz que está bom a forma como escreveu.

				R2 ainda sentada na sala de evolução, pergunta a preceptora como ela classificou a lesão por pressão nos calcâneos do paciente x. Preceptora diz que classificou como estágio I, R2 diz "não concordo não, tem tipo um hematoma no local, um hematoma abaixo da bolha". Preceptora diz que ainda não viu a lesão, R2 detalha mais ainda as características da lesão para a preceptora que volta a evoluir. R2 volta a mexer no computador. Quando preceptora finaliza suas evoluções, R2 a chama para avaliar a lesão do paciente. As duas avaliam os calcâneos, ficam em dúvida se a bolha está com sangue para caracterizá-la como tissular profunda. Coordenadora de enfermagem e enfermeira passam pelo corredor, param para conversar com a preceptora, e então começam a avaliar a lesão e opinam sobre o estágio. R2, preceptora, enfermeira e coordenadora discutem sobre a LP, R2 fica atenta a conversa. Chegam à conclusão que é lesão tissular profunda, então coordenadora diz ter em sua sala duas placas de espuma, pergunta se querem colocar no local, preceptora e R2 concordam. Coordenadora busca as placas e começam a colocar as placas no calcâneo. R2 coloca as placas e preceptora diz que se fosse lesão superficial, não estaria com o líquido de sangue dentro da bolha. R2 concorda com a fala e acrescenta "depois precisamos confirmar se essa espuma são 7 dias de permanência, você sabe?". Preceptora não sabe o tempo de permanência. Até o final do plantão não buscaram pela informação de tempo de permanência.
				Ao finalizar, R2 começa a preencher o formulário de avaliação da qualidade da espuma que a coordenadora de enfermagem solicitou preencher. Preceptora fica ao seu lado dando suporte no preenchimento. R2 preenche formulário cuidadosamente para não errar, demonstra insegurança com as informações que precisa escrever no formulário, então olha para preceptora que percebe sua dificuldade e insegurança. Assim, preceptora começa a dizer a R2 o que é importante ser inserido no formulário. Após a sua fala, R2 diz "não seria mais lógico fazermos essa avaliação daqui 7 dias? Assim conseguimos acompanhar a qualidade da espuma, sua aderência na pele...". Preceptora concorda com a fala de R2 e sugere deixar o formulário na prancheta do paciente e acompanhar diariamente a espuma na pele da paciente.
				R2 pergunta a preceptora como libera entrada de visitante extra, preceptora explica e R2 escreve memorando liberando visita adicional para o paciente.
				Resolve problemas de bomba de infusão e monitor apitando. Em alguns momentos bomba de infusão apita por obstrução, em outros para indicar término de infusão, etc.
				Paciente chama R2 e queixa de vontade de urinar, R2 diz ao paciente que ele está com SVD, então não precisa se preocupar que a diurese sai automático à medida que o organismo produz a urina. Mas paciente insiste que está com muita vontade, R2 coloca uma luva de procedimento e avalia presença de bexigoma, palpa região supra púbica e pergunta ao paciente se há presença de dor, paciente diz não estar com dor. Então avalia se o circuito da SVD está dobrado e percebe que não. Prepara material e faz teste de obstrução de sonda, pelo teste confirma que o circuito não se encontra obstruído. Caminha em direção ao TE responsável pelo paciente e pergunta se o paciente está urinando, TE responde que paciente já urinou 700ml e que acabou de fazer uma furosemida. R2 diz "ahh... então não está obstruído não!"
				TE chega e comunica que terá punção de CDL no paciente y, R2 vai ao computador e solicita materiais de punção de CDL, imprime e vai a farmácia satélite buscar materiais. Entrega os materiais ao TE.
				R2 passa pelo corredor e percebe que paciente se encontra desmonitorizada, começa a monitorizar a paciente, mas ela não deixa, R2 insiste na monitorização e explica a paciente a importância de monitorizá-la. Ao finalizar a monitorização, olha para o monitor e vê uma frequência cardíaca de 28 bpm e diz "nossa senhora!" (R2 não faz nada, pois na transferência de cuidados, enfermeira da manhã havia comentado que a paciente ficava com a FC muito baixa). R2 coloca paciente sentada no leito e oferece o jantar para a paciente. Ao final, sai do box e comenta com preceptora que nunca tinha visto uma FC tão baixa, diz ainda que paciente não sente nada com a FC baixa.
				R2 evolui os seus pacientes no sistema eletrônico e faz fechamento do plantão.

				Ao finalizar, auxilia a preceptora no procedimento de punção de CDL. Pega folha de procedimento cirúrgico e preenche à medida que os materiais vão sendo utilizados. Por fim, fornece materiais para o médico fazer o curativo. Sai do procedimento e auxilia técnico a posicionar outro paciente e colocar placa de raio x. Após o raio x, auxilia novamente a retirar a placa e posiciona paciente novamente.
09/09/2022	07:00	13:20	Semi-intensivo	Quando R2 chega ao plantão, sua preceptora já havia pegado os casos dos pacientes. Então preceptora passa para R2 apenas os pacientes sob a sua responsabilidade. R2 escuta, anota algumas informações relevantes em seu passômetro e à medida que preceptora realiza a transferência de cuidados, R2 diz "aham..." ou balança a cabeça.
				Ao finalizar a transferência de cuidados, R2 se levanta e caminha em direção ao carrinho de PCR. Realiza teste do desfibrilador, laringo e checa presença de tábua de PCR e número do lacre do carrinho. Ao realizar o teste do laringo, identifica que está faltando uma lâmina, então vai ao outro carrinho e encontra. Ao final preenche caderno de controle de teste de desfibrilador e carrinho de PCR. R2 faz as mesmas atividades no segundo carrinho de PCR.
				Bomba de infusão (BI) apita, R2 caminha em direção a BI e lê no visor "obstrução". Começa a verificar o que está infundindo em cada equipo e em cada via do CVC. Neste instante, fica um tempo parada com a testa franzida e olhando para o CVC. Depois de um tempo, olha em direção ao corredor na tentativa de chamar alguém, não passa ninguém, então volta a manipular os equipos e silencia novamente a BI. Sai do leito e vai em direção a TE responsável pelo paciente e diz: "Você quem está com o paciente X não é? Acho que o midazolam precipitou!". TE diz: "deve ter infundido meropenem na mesma via do midazolam!". R2 e TE retorna para o leito do paciente e observam as conexões do CVC. R2 diz: "parece que já precipitou dentro da veia". TE sai do leito e R2 começa a fazer exame físico do paciente, à medida que faz o exame físico vai fazendo anotações no passômetro. Verifica PIA, CVD, checa pupilas, etc. Ao final, observa novamente o CVC.
				Avalia o segundo paciente, verifica prescrição médica. Examina os MMII, palpa panturrilha, pergunta ao paciente se ele sabe qual dia é hoje, qual mês e ano nós estamos e o que tem de comorbidade. Checa datas de CVPs e curativos, ao final anota informações no passômetro.
				Avalia o terceiro paciente, checa PIA, CVC, PIC e CVD, checa medicações que estão infundindo, olha os rótulos e quantidade; palpa abdômen e checa pupilas. BI apita "bateria acabando", então ajuda TE a resolver problema de mal contato da tomada. Sai do leito e avisa a preceptora que irá tomar café.
				Ao retornar do café, senta-se ao lado da preceptora para evoluir. Comenta com a preceptora que assustou ao avaliar o paciente 3, pois na 3ª feira o paciente estava bem e achou que ele receberia alta no dia seguinte e hoje ao avaliar se deparou com o paciente todo invadido. Preceptora diz que não sabe o que aconteceu, então R2 diz que vai olhar no passômetro médico para entender o que aconteceu. Retorna para sua evolução e em alguns momentos lê passômetro médico, verifica impressos de sinais vitais e lê anotações de seu passômetro. Ao finalizar evolução os 3 pacientes, elabora os diagnósticos e prescrição de enfermagem.
				R2 comenta sobre a precipitação do midazolam no CVC e pergunta o que fazer nesta situação. Preceptora caminha em direção ao paciente e fala para R2 que normalmente a precipitação obstrui a via do CVC, diz que é necessário aspirar a medicação precipitada, mas não garante que 100% da medicação conseguirá ser aspirada. Preceptora desconecta o equipo de medicação precipitada do CVC e começa a fazer bolus na BI com o objetivo de sair todo o volume que receptou. Pega uma seringa e começa a aspirar a via, vem retornando à medicação precipitada com sangue. Neste momento R2 encontra-se ao lado da preceptora e vai a auxiliando fornecendo os materiais necessários. R2 atenta a fala da preceptora e observa com atenção as técnicas que preceptora desenvolve. Ao final, R2 verifica os calcâneos do paciente e conta para a preceptora que ela e a

				preceptora da tarde colocaram espuma nos calcâneos do paciente e que agora acompanham diariamente a qualidade da espuma para preencher formulário de qualidade do insumo.
				R2 e preceptora passam por um paciente que está recebendo banho no leito, preceptora observa pele da paciente e acha estranho paciente ter LPP, R2 então olha no passômetro data de admissão da paciente no setor e diz: "paciente chegou dia 7 de setembro não deu tempo de ter essa hiperemia na sacral e trocantérica", preceptora diz que vai ler a evolução da admissão da paciente e sai para buscar filme transparente. R2 segue preceptora e diz "não deu tempo de abrir lesão não, deu?", preceptora diz: "acredito que não".
				R2 vê que a corrida de leito médica está acontecendo nos pacientes da preceptora chega próximo e fica em pé escutando. Apenas o médico falou.
				Chega uma TE externa no box de um de seus pacientes e R2 percebe, vai em direção a ela e pergunta "o que vai fazer aí?" funcionária responde que irá fazer o 3º EEG no paciente para ver se ele mantém status epiléptico. Funcionária explica com detalhes que é necessário repetir o EEG, pois houve redução da dose de midazolam e ácido valproico. R2 escuta com atenção a funcionária, balança cabeça concordando com a fala e diz "ahh entendi, não sabia que o acompanhamento era assim!" e depois se senta na sala de enfermagem e registra em sua evolução o que a funcionária a explicou.
				Organiza materiais para passagem de CNE, ao se aproximar do paciente e explicar o procedimento, TE diz: "não acha melhor eu fazer o Diazepam primeiro?", R2 concorda com a fala. (paciente muito agitado, tentando descer da cama e já contido por risco de queda e tentativa de retirada de dispositivos invasivos). Então, R2 vai preparando fixação de CNE. Vai ao médico e pergunta se paciente que foi avaliado pela fono vai receber CNE também, médico diz que sim. Retorna ao leito, monitoriza paciente agitado, pede a ele para ficar mais calmo. Ao finalizar a monitorização percebe que saturação de O2 está mais baixa. Posiciona cateter nasal. Curva de saturação ruim, R2 e preceptora fica um bom tempo tentando fazer oxímetro funcionar, paciente não colabora. Preceptora diz a R2 que o oxímetro é pediátrico, então é mais difícil de pegar mesmo. Diz que não tem oxímetro na unidade. Preceptora chama fisioterapia para avaliar paciente, que aumenta vazão de oxigênio. R2 olha no parâmetro do paciente a vazão do oxigênio previamente e fala com a preceptora. Médico passa e diz que paciente está taquicárdico e solicita um ECG. R2 comenta com preceptora "até parece que paciente vai ficar quieto para fazer esse ECG", R2 sai e começa a acompanhar corrida de leito de seus pacientes.
				Ao acompanhar corrida de leito, R2 atenta a fala do médico e residente de medicina, acompanha com o olhar as pessoas que falam, anota informações importantes em seu passômetro. Preceptora também acompanha a discussão de casos, ficam em silêncio durante toda a corrida de leito.
				Ao final da corrida de leitos, R2 sai para evoluir, utiliza suas anotações como referência. Durante a evolução de seus pacientes, R2 também lê evoluções da equipe médica e abre aba do sistema eletrônico para ver os sinais vitais dos pacientes. Preceptora entra na sala e entrega um leito de alta para R2.
				R2 finaliza suas evoluções e sai para buscar impresso de transferência de cuidados. Retorna para o computador e inicia o preenchimento do impresso. Ao preencher o impresso, R2 abre evoluções da equipe médica e do TE, olha suas anotações no passômetro e então escreve no impresso o histórico do paciente, locais de CVP, recomendações, etc.
				Ao terminar de preencher o impresso de transferência de cuidados, R2 vai em direção a TE responsável pelo paciente de alta e comunica leito. Além disso, diz "vi no sistema que o CVP do paciente vence hoje, pode puncionar outro, por favor? Vou passar o caso do paciente!". Caminha até o telefone e inicia a transferência de cuidados do paciente, R2 passa as informações confiante do que está falando e com domínio do caso. Ao finalizar, volta para a TE e diz que vão remanejar alguns pacientes na enfermaria para então, receber o paciente.

				Organiza material para passar CNE no paciente que irá de alta. Orienta paciente e inicia a passagem do cateter. Paciente foi colaborativo durante o procedimento, R2 pedia para ele engolir e abaixar o queixo, paciente fazia tudo que era solicitado. R2 cuidadosa e segura. Ao final faz fixação do cateter. Aspira o cateter, ausência de estase. Ausculta a região epigástrica e depois diz ao paciente: "deu certinho, o senhor só não pode arrancar, se não vou precisar passar de novo". Guarda o fio guia na gaveta e despreza resíduos. Sai do box e chega para preceptora e diz "deu certo! Passei na narina direita de jeito que me falou!". R2 diz com a face de alegria.
				R2 e preceptora vão em direção ao paciente agitado, seguram para ele não cair da cama. Médico chega com prescrição de Diazepam (IM). TE chega no box e R2 sai para passar outro CNE.
				R2 organiza material para passar CNE no paciente que a fonoaudióloga avaliou. Orienta o paciente e inicia a passagem do cateter. Ao tentar passar na narina esquerda, R2 percebe resistência e paciente movimenta a cabeça na tentativa de sair. R2 retira CNE, passa mais xilocaína geleia e diz para paciente ajudá-la. Tenta novamente na narina esquerda, percebe novamente resistência, movimenta cabeça do paciente, faz outra tentativa e não consegue. Retira CNE. R2 diz ao paciente que vai tentar passar o CNE na narina direita, diz ao paciente que a esquerda tem resistência. Passa CNE na narina direita, consegue descer com o cateter. R2 cuidadosa e segura. Ao final faz fixação do cateter. Aspira o cateter, ausência de estase. Ausculta a região epigástrica e não consegue escutar o som, realiza o teste mais 2 vezes e nada. Fica uns instantes parada. Então retira a fixação e volta um pouco com a sonda, depois desce novamente. Ausculta e diz ao paciente que precisa fazer um raio x para confirmar o posicionamento. Reúne os resíduos e despreza.
				Evolui no sistema eletrônico os procedimentos. Enfermeira do vespertino chega e R2 começa a passar os seus pacientes, enquanto preceptora terminava suas evoluções. Enfermeira recebe os casos e diz que conhece todos os pacientes. R2 passa os pacientes dizendo detalhes de cada um, como propostas definidas na corrida de leito e pendências de cada paciente.
13/09/2022	07:00	13:30	CTI	Transferência de cuidados entre a enfermeira do noturno, preceptora e R2. R2 escuta os casos e faz anotações em passômetro. Enfermeira do noturno ao contar caso clínico do paciente, R2 pergunta: "porque ele está ficando bradicárdico assim?". Enf. responde: "não sei, cardiologia vem avaliar, acreditam ser problema no CDI". R2: "Nossa"
				Ao finalizar as transferências de cuidados, R2 sai da sala de enfermagem e inicia a avaliação de seus pacientes. 1º paciente: chama pelo paciente, pergunta sobre dor, verifica medicações que estão nas bombas de infusão, palpa abdômen, verifica PIA, CVC e CVD e ausculta CNE.
				Paciente 2: paciente contido no leito, confuso e agitado. Paciente com fala desconexa, R2 não consegue entendê-lo. Organiza o leito do paciente, diz pensando alto "bagunça". Tenta monitorizar paciente, mas não consegue, paciente arranca. Tenta novamente, consegue checar dados e anota nos impressos. Identifica que paciente está hipertenso, pega prescrição médica e começa a ler, parece procurar alguma medicação específica.
				Segue para o paciente 3 e começa a verificar os impressos da prancheta do TE. Fisioterapeuta chega próximo ao paciente e diz: "esse paciente está todo pintado, não é varíola, não?" R2 diz: "ele está assim desde ontem!" Fisio: "será que é reação a ATB? Não fiquem encostando muito nesse paciente não, hein!" R2: "isso apareceu no domingo". Fisio sai do leito e preceptora chega e começa a pesquisar no google como é o aspecto das feridas de varíola, R2 olha para a tela do celular e depois as duas vão em direção ao paciente e avalia a sua pele. Preceptora: "do outro lado (do corpo) está pior!" R2: "não está parecendo varíola não!". Preceptora sai em direção a equipe médica, ao retornar para o leito do paciente diz "ninguém sabe o que é". R2 realiza um exame físico minucioso no paciente e diz a preceptora "esse paciente está tomando ivermectina e vários anticonvulsivantes". Preceptora: "tem que pensar em isolamento" R2: "se está suspeitando que é algo, tem que pensar em isolamento!". Preceptora e R2 ficam observando a pele do paciente e pensando o que poderia ser aquelas pústulas e pápulas.

				Sai do paciente e vai para sala de prescrições e começa a evoluir os 3 pacientes. Preceptora passa e diz "R2 não deixe de evoluir não" (evoluir as lesões de pele). R2: "já evoluí ontem, hoje vou escrever que piorou". Na evolução dos pacientes preenche aba SAE, integridade cutânea, diagnósticos e prescrição de enfermagem. Olha anotações em passômetro e registra.
				Passa pelo paciente 2, vê médica avaliando paciente e diz "este paciente está hipertenso". Médica "mas nessa agitação!". Médica pede para retirar contenção dos pés para avaliar se conseguem dar alta no paciente. R2 solicita TE para retirar e avaliar.
				Realiza testes dos desfibriladores, laringos, verifica se carrinhos de PCR estão lacrados e ao final preenche e cola no caderno de teste do desfibrilador os impressos.
				Retorna do café e oferece café da manhã para paciente 2, vai colocando mingau na boca do paciente que tem dificuldade para aceitar. Insiste por um bom tempo e com paciência.
				Prepara paciente para tomografia. Pergunta para preceptora se durante o transporte vai infundindo propofol. Preceptora e R2 monitorizam paciente, pega maleta de transporte. Monitor não liga, R2 diz "estava acusando bateria carregada quando peguei", sai do leito para trocar o monitor. R2 pergunta "vamos levar contido?" Preceptora: "acho melhor". R2 saliniza uma via do CVC. Fisio deixa uma parte do circuito do VM de transporte cair no chão e reclama em voz alta dizendo ser o último circuito da unidade. Enquanto tenta encontrar outra conexão, R2 olha para o VM e circuito que já estão conectados no paciente e franze sua testa, diz "tenta pensar em uma conexão que dê para substituir", fisioterapeuta e R2 fazem uma "gambiarra" na conexão do VM, testam a "gambiarra" e checam se deu certo. Ao ver que deu certo dão uma risada e saem para a TC (residente médico, R2 e preceptora). Ao chegar na TC, passam paciente para a mesa. TE da tomografia começa a manipular a bala de O2 e R2 diz "deixa na bala mesmo", TE "melhor passar para a parede". Exame mais demorado para ser feito, pois paciente agita, residente de medicina solicita administrar um bolus de sedação. Retornam para o CTI e organizam paciente e materiais no box.
				R2 chega para TE e pergunta se ela trocou curativo do paciente 2, TE diz que não. Então chega para a preceptora e explica para ela o aspecto da lesão e diz que não sabe se a lesão é proveniente de agitação, de contenção ou se é uma LPP. Vão ao paciente, preceptora o segura para R2 fazer a troca do curativo. Conversam sobre a ferida, cogitam algumas possibilidades do motivo de ter aparecido a lesão e definem utilizar petrolato no local. Paciente não colabora durante a troca do curativo, R2 tem dificuldades de higienizar e colocar a cobertura. Preceptora diz que paciente está fazendo muita força contrária que está difícil de segurá-lo. R2 começa a trocar o curativo mais rápido e ao finalizar diz "vou colocar mais filme, da forma que ficou vai sair rapidinho (o curativo)".
				Início da corrida de leito, anota as propostas no passômetro, em um determinado momento o barulho ambiente aumenta, R2 se aproxima da residente de medicina que está falando sobre os casos. Fica com o olhar direcionado para o profissional que fala. Durante a corrida de leito, fica ao lado da preceptora a maior parte do tempo. Durante a corrida de leito, recebe leito de alta da enfermeira responsável pela gestão de leitos.
				Término da corrida de leito, avisa para a TE responsável pelo paciente 2 que o mesmo está de alta e que o leito de alta já saiu. Diz que irá fazer a transferência de cuidados. Pega o impresso de transferência de cuidados, preenche de acordo com suas anotações e passômetro médico que está impresso na bancada do posto de enfermagem. Lê o passômetro médico e à medida que lê, vai anotando algumas informações no impresso de alta. Ao finalizar, liga para a enfermaria e recebe a notícia que o leito não está disponível mais. Então avisa a preceptora que diz que vai avisar para o NIR.

				Senta-se para evoluir a lesão do paciente 2, durante a evolução se levanta e vai na preceptora e pergunta se lesão por fricção é considerada LPP. Preceptora diz não saber, enfermeira que está ao lado da preceptora começa a pesquisar no google, lê em voz alta o que encontrou e preceptora e R2 ficam escutando. Ao final da leitura ficam discutindo sobre o que leram/escutaram e ficam discutindo sobre a classificação da lesão e concluem que não é uma LPP. R2 então ao sair da sala, preceptora diz em voz alta "olha no folder de lesões daqui do hospital, talvez tem alguma coisa". R2 sai em direção ao folder que está colado na parede, lê e diz pensando alto "não é LPP". Volta a sala dos enfermeiros e diz a preceptora que não é LPP. R2 então, explica para preceptora e enfermeira sobre outras lesões que também estão descritas no folder como lesão por pressão proveniente de dispositivos como three way, lesões por esparadrapo e micropore e lesão de Kennedy. Ao final da fala, sai e retorna a evoluir.
				Termina evolução e anda pelo CTI observando os pacientes, vê paciente com saturação de 62%, para assustada, mas percebe que nutricionista está avaliando o paciente e ao chegar no box, nutricionista diz que a saturação já está subindo. R2 espera um pouco, olhando para o monitor, mas por estar subindo muito lento, sai do leito, procura fisioterapeuta e fala sobre a saturação. Retorna ao paciente e eleva FIO2. Fica no box até paciente se recuperar.
				Recebe leito de alta do paciente 2, liga na enfermaria e realiza a transferência de cuidados. R2 fala ao telefone de forma segura. Ao final da ligação, fala com TE que já pode levar paciente de alta. Então ajuda a TE a organizar paciente para sair de alta.
15/09/2022	14:00	15:30	Sala de aula	R2 apresenta estudo de caso sobre traumatismo craniano por meio de slides. Apresenta fisiopatologia da hipertensão intracraniana; tratamento; monitorização e cuidados de enfermagem; etc. R2 faz sua apresentação se guiando pelos slides, demonstra domínio sobre o assunto, mas passa que está nervosa. Falas frequentes: "eh..."; repete com frequência o barulho da saliva ao exercer pressão da língua no céu da boca. À medida que fala, movimenta suas mãos, articulando e encostando uma a outra. No slide em que explica a curva da PIC ao monitor, R2 diz: "nossa... Estou nervosa!". Ao falar sobre o valor sustentado de PIC diz "como o preceptor disse aquele dia, podemos deixar a DVE aberta, assim quando a PIC estiver maior que 20 mmHg, ocorre drenagem espontânea".
				Ao final da apresentação, coordenadora cobra a presença dos diagnósticos e a prescrição de enfermagem na apresentação. R2 diz que havia colocado na apresentação, mas depois retirou por achar repetitivo. R1 diz que não entendeu a explicação da curva de PIC, R2 explica novamente, utiliza o desenho do slide para auxiliá-la. Em seguida, enfermeiro complementa a explicação. R2 fica olhando atentamente para o enfermeiro, balança a cabeça em alguns momentos da fala.

22/09/2022	14:30	17:00	Sala de aula	Enquanto T1 discursa sobre o programa de residência, R2 fica olhando para T1 e balançando a cabeça no sentido de estar concordando com a fala. Em alguns momentos, R2 ri de algumas falas engraçadas da T1. Outra professora se apresenta, fala sobre sua trajetória acadêmica e profissional, R2 continua atenta a fala. T1 solicita as residentes a se apresentarem. Primeira residente se apresenta e fala sobre as suas experiências. Expõe que não está gostando do bloco cirúrgico, T1 explica o motivo e a importância de os residentes terem a experiência dos processos de trabalho do BC. R2 continua atenta à conversa, direcionando seu olhar para quem fala. T1 fala sobre a possibilidade de o programa fazer parceria com o SAMU, R2 sorri, no sentido de ter gostado da informação que escutou. T1 fala das dificuldades durante a pandemia. Segunda residente se apresenta. R2 se apresenta, fala o ano em que formou, onde trabalhou, sobre sua formação, ano e local. Ao final, T1 começa a explicar o cronograma da residência, o motivo de folgarem nas quintas feiras a tarde, sendo uma quinta destinada ao estudo de caso e a outra quinta destinada a preparação do estudo de caso. R2 continua balançando e concordando com a fala de T1. T1 fala sobre a distribuição da carga horária na residência, R2 solicita fala e explica a divisão da carga horária nos eixos comuns, transversais e específico. Então T1 cita as dificuldades para fornecer aulas teóricas e R2 acrescenta que se sente prejudicada por não ter tempo para estudar e descansar, por trabalhar de segunda a sexta 12h. No decorrer da conversa, T1 percebe que está havendo um mal-entendido e esclarece que as tardes das quintas-feiras são destinadas aos estudos de caso e a tarde de qualquer outro dia da semana destinada para a tutoria. Durante a fala de T1, R2 começa a rir e diz "melhor assim". Então T1 continua sua explicação, enquanto escuta R2 mexe no cabelo e fica com o olhar vago (no sentindo de estar pensando em outras coisas). Encosta a cabeça na parede e continua olhando para T1. T1 fala sobre a construção do TCR e R2 diz "aham" e ao final explica de forma superficial a rede de assistência à saúde de BH.

Fonte: Elaborado pelas autoras.

O quadro 3 apresenta as descrições das atividades desenvolvidas pelo Residente 3 (R3) do primeiro ano do PRAPS Modalidade Uniprofissional Enfermagem Obstétrica em 32 horas e 10 minutos de observação.

Quadro 3 - Descrição das atividades desenvolvidas pelo R3 em 32 horas e 10 minutos de observação.

**Data Observação**	**Horário Início**	**Horário Término**	**Setor Observação**	**Descrição das atividades realizadas pelos residentes**
15/09/2022	07:00	12:40	Ambulatório Saúde da Mulher	Primeira consulta: avalia se paciente é apta para inserção de DIU. Paciente entra no consultório e R3 inicia a consulta com a entrevista da paciente. Ao final, R3 diz a paciente que a mesma está apta para inserir DIU. Então orienta paciente em relação ao TCLE e a impresso sobre a responsabilização da paciente em relação a veracidade das informações fornecidas durante a entrevista. Enquanto paciente lê o TCLE e impresso, R3 sai do consultório e vai a secretaria e pergunta: "meninas, aquele

				impresso de inserção de DIU é assinado aqui na recepção, não é?" segue com a fala que paciente chegou ao consultório com o impresso em branco. Então, uma das secretárias diz que a orientação foi alterada e que agora, o impresso é preenchido na sala de espera ou consultório e orientado pelo profissional durante a consulta. Então, R3 sai e procura a preceptora a fim de entender melhor o fluxo, ao conversar com preceptora, a mesma confirma a alteração do fluxo. R3 diz que não sabia e retorna para o consultório. Ao chegar no consultório, inicia a orientação sobre inserção do DIU. Utiliza uma almofada pequena em formato de útero e um DIU para fazer toda a orientação de inserção e cuidados para manutenção do DIU. Paciente escuta toda a explicação e assina as documentações. R3 sai do consultório para buscar materiais e paciente entra para o banheiro para colocar a camisola.
				Paciente se deita na maca e R3 diz que iniciará o procedimento. Inicialmente faz o toque uterino, durante o toque fica em silêncio e fecha os olhos, dando a sensação de que ela está muito concentrada na ação que desenvolve. Ao final diz "deu para sentir direitinho o colo". Prepara o material na mesa de procedimentos e enquanto isso, orienta paciente sobre o retorno da consulta e monitorização do DIU. À medida que desenvolve o procedimento vai avisando a paciente o que ela está fazendo. Introduz o espéculo vaginal, observa, higieniza e pinça o colo. Diz a paciente que fará a medida do colo, salienta que irá doer. Em seguida, tenta inserir o DIU, sem sucesso. Diz a paciente que está difícil a inserção. Faz novamente a histerometria e em seguida fica parada olhando para o espéculo. Diz a paciente sobre a resistência que sentiu ao inserir o DIU e que chamará outro profissional para fazer a inserção. Sai do consultório, procura preceptora, a mesma encontra em atendimento. Então, pede ajuda para outra enfermeira, explica com detalhe sua dificuldade de inserir o DIU.
				Enfermeira e R3 vão para o consultório e se deparam com paciente saindo do banheiro e dizendo que está com uma cólica muito forte. Enfermeira diz para prescrever medicação para dor, R3 se senta e começa a preencher prescrição. Pergunta qual medicação que deve prescrever e enfermeira responde dizendo 2 medicações diferentes. R3 começa a prescrever, fica parada com a caneta e olhando para o papel, em seguida diz: "dipirona é 500 mg, não é?", Enf. "não, 1g". R3 demonstra insegurança neste momento diz: "confundo um pouco isso". Enfermeira começa a ditar o que é necessário prescrever, dizendo o nome da medicação, dosagem, diluição e via de administração. Enfermeira pega prescrição e leva no posto de enfermagem, enquanto isso R3 pede para paciente se deitar e aguardar a dor passar. Enfermeira retorna e esperam cerca de 20 minutos a paciente se acalmar.
				Paciente autoriza o reinício do procedimento. Enfermeira então, introduz espéculo. R3 fica atrás da enfermeira observando tudo que enfermeira executa e a auxilia. Enfermeira diz que a visualização está difícil e paciente diz estar com dor. TE chega e administra as medicações. Esperam mais uns 15 minutos. Enquanto isso, R3 explica com mais detalhes todos os passos que desenvolveu e o que viu ao tentar inserir o DIU. Enfermeira inicia novamente o procedimento, insere o espéculo, visualiza o colo uterino, faz a histerometria e insere o DIU. Durante todo o procedimento, R3 se posicionou atrás da enfermeira e inclinou seu corpo para frente para facilitar a visualização do procedimento. Ao final, enfermeira diz para R3 olhar onde está o fio do DIU e explica como deve deixá-lo. Em seguida, reúnem material e desprezam resíduos. Enfermeira sai do consultório e R3 finaliza a consulta fazendo um resumo das principais orientações e paciente vai embora. (primeira consulta e inserção de DIU extensa, duração de 2 horas. R3 sai para tomar café)
				Quando R3 retorna do café, enfermeira chama R3 e conta sobre a história da paciente que acabou de atender, fala detalhes. Ao final, enfermeira comenta sobre a primeira consulta e orienta a R3 sobre a recomendação dos estudos de não tentar inserir o DIU mais de 2 vezes. Então orienta R3 de nas próximas situações, ao não conseguir inserir o DIU da primeira vez, chamar a preceptora para que ela tenha a oportunidade de passar. R3 escuta a orientação, balança a cabeça concordando com a fala.

				Segunda consulta: avalia se paciente é apta para inserção de DIU. Paciente entra no consultório e R3 inicia a consulta com a entrevista da paciente. Ao final, R3 diz a paciente que a mesma está apta para inserir DIU. Então orienta paciente em relação ao TCLE e a impresso sobre a responsabilização da paciente em relação a veracidade das informações fornecidas durante a entrevista. Paciente diz que trouxe o DIU mirena para ser implantado. Orienta paciente a ir ao banheiro para colocar camisola e sai do consultório. Encontra com preceptora e diz que tem um DIU de mirena para inserir. Preceptora pergunta o motivo dela não querer inserir, R3 diz ser um DIU muito caro, que fica com medo de perder o DIU. Então preceptora e R3 vão para o consultório.
				Preparam o material, paciente se deita na maca e preceptora inicia o procedimento. Ao inserir o espéculo vaginal, preceptora chama R3 e pede a mesma para descrever o aspecto da parede do canal vaginal. R3 diz: "nossa, estou achando sem coloração e pouca secreção". Preceptora diz a paciente que vai solicitar um beta-HCG antes de fazer a inserção. Paciente diz ser histerectomizada. R3 diz: "você não me disse isso! É muito importante você me falar a verdade!". Então na conversa com paciente, preceptora e R3 descobrem que paciente quer colocar o DIU para interromper o sangramento contínuo que a mesma tem. R3 insere as informações adicionais no impresso e solicita paciente para assinar o TCLE e impresso novamente. Preceptora insere o DIU e R3 se posiciona atrás da preceptora, inclina o corpo e observa todos os passos executados. Ao finalizar o procedimento R3 diz a preceptora "o mirena é mais fácil para passar, né!". Preceptora "sim, mais prático". Preceptora sai do consultório e R3 finaliza as orientações do paciente. Quando paciente está saindo do consultório, R3 diz que ela está esquecendo de levar o folder de orientação e a documentação, paciente diz que pode jogar tudo fora, neste momento R3 balança a cabeça de um lado para o outro no sentido de "não tô acreditando nisso". R3 então, sai do consultório e procura preceptora e diz: "tive muito cuidado com os impressos, pedi a paciente para assinar tudo!" depois desta fala, R3 faz seus comentários em relação a postura da paciente durante a consulta e inserção do DIU e sobre a omissão de informações. R3 se demonstra indignada com o que presenciou. Preceptora orienta como proceder nestes tipos de situações.
				Após conversar com preceptora, R3 vai ao banheiro, toma uma água e utiliza o celular por um tempo. Depois vai para o posto de enfermagem conversa assuntos não relacionados ao trabalho. Preceptora chega no posto de enfermagem, R3 se aproxima dela e pergunta: "o que você achou do colo daquela paciente?". Preceptora detalha sua percepção sobre o colo e explica o motivo que a fez dizer que iria solicitar um beta HCG. Então R3 diz: "ahh sim, eu também pensei nisso quando me perguntou!" e faz uma cara de satisfação. Em seguida, senta-se com a evolução da paciente em mãos, fica um tempo olhando para o impresso e depois começa a escrever sua evolução.
				Terceira consulta: revisão de DIU. Chama paciente para o consultório, faz uma entrevista geral, pergunta como está a adaptação com o DIU, se houve escape sanguíneo, se usou método contraceptivo no período entre a inserção até a revisão (hoje), etc. Em seguida solicita paciente a colocar a camisola no banheiro, enquanto isso anota as respostas da paciente no impresso de consulta. Então paciente se deita na maca e R3 explica o que fará, insere o espéculo e procura o fio do DIU, não encontra. Então diz a paciente que chamará preceptora para auxiliar na visualização.
				Preceptora chega à sala e também não visualiza o DIU. Neste momento, se posiciona atrás da preceptora, inclina corpo e fica na ponta dos pés para ver o que a preceptora está fazendo ao procurar o fio do DIU. Ao final, preceptora confirma que também não visualizou, então conversam sobre a situação e acham melhor solicitar um ultrassom. R3 explica a paciente o que será necessário fazer. Então R3 faz a solicitação do Ultrassom, vai no consultório médico para encaixar um horário para o ultrassom da paciente e ao final retorna ao seu consultório e orienta paciente.
19/09/2022	13:00	20:00		Preceptora retorna do horário de almoço, então R3 passa os eventos que aconteceram durante seu horário de descanso. Passa caso de duas admissões que receberão, utiliza prontuário para contar as histórias das pacientes.

			Unidade Gestante de Alto Risco	Ao finalizar, chega paciente 1 de admissão, R3 auxilia transferir paciente para cama, monitoriza e detecta hipotermia do RN. TE aquece a mãe e R3 autoriza pai entrar para o leito e orienta o pai manter contato pele a pele com a RN. Auxilia o pai a se posicionar na poltrona com o RN.
				TE comunica valor de glicemia capilar de RN, R3 diz não saber, então pega protocolo de controle glicêmico de RN e começa a ler, em seguida orienta TE colocar RN para amamentar e daqui 1 hora repetir a glicemia capilar.
				R3 avalia paciente 2 que está em crise de ansiedade, conversa com a preceptora sobre a importância de a equipe saber lidar com tal situação, disse que a equipe não está ligando para os relatos da paciente. Diz ainda para a preceptora solicitar a médica para prescrever alguma medicação para controlar a ansiedade da paciente. Ao final relata que já passou por crises de ansiedade e expressa o tanto que é ruim.
				A residente de medicina que se encontra no setor é da Angola, R3 e residente trocam experiências em relação aos programas de residência e como é o sistema de saúde de cada país.
				Evolui paciente 2 que chegou de admissão, em alguns momentos chega no acompanhante e pergunta algo sobre a paciente, como quanto tempo parou de tomar sertralina; qual a frequência das crises; e depois retorna para a bancada e continua sua evolução. Abre prontuário e faz leitura de evoluções de outros profissionais.
				Inicia a evolução da admissão do paciente 1, diz pensando alto que não entendeu o motivo que paciente foi recebida no setor, procura em prontuário evolução médica e lê. Em seguida, vai a sala do descanso médico e pergunta o motivo da paciente não ter ido para enfermaria. Médica diz que paciente está com hemoglobina baixa e que precisa ser monitorada.
				Atende o telefone, equipe do Paulo de Tarso solicitando passar o caso do paciente que irão receber, pega prontuário da paciente e realiza a transferência de cuidados com domínio sobre o caso. Em seguida comunica a TE que pode preparar paciente para o transporte.
				Preceptora avisa que precisa fazer um cardiotocografia (CTG) na paciente 3, R3 pega o aparelho e antes de conectar na paciente explica para a paciente e acompanhante para que serve o exame e em seguida conecta em paciente. Exame com muita interferência, movimenta conectores, não consegue resolver. Então, R3 parada olhando para a tela do aparelho e elevando as sobrancelhas, olha para a paciente e a solicita lateralizar para a esquerda. Após a mudança de posição, resolve problemas de interferência e diz a paciente que a deixará fazendo o exame por uns 30 minutos e sai para atender outra paciente.
				Realiza exame físico em recém-nascido, conta a frequência cardíaca, ausculta, avalia coloração, céu da boca e cabeça. Faz vários testes de reflexo. Ao examinar região genital, percebe diurese em fralda, então pergunta ao pai se sabe trocar fralda, diz que não. Então, R3 ensina o pai a realizar a primeira troca de fralda, em seguida coloca RN no peito da mãe e faz todas as orientações em relação a pega correta do peito. Realiza exame físico na mãe. Enquanto faz o atendimento do RN e da puérpera, preceptora organiza e sai para transporte de um paciente que será transferido para outra instituição de saúde. Preceptora orienta chamar enfermeiras do PA em caso de dúvidas ou intercorrência.
				Retorna para a paciente 3 e desliga o CTG. Pega o resultado e vai para a bancada do posto de enfermagem, fica um tempo fazendo a análise do exame e diz pensando alto "RN está taquicárdico", então caminha para o PA e procura enfermeira obstétrica, mostra e trocam uma ideia sobre o resultado do exame, concluem que não há alteração, mas enfermeira solicita R3 a mostrar para a equipe médica da UGAR. R3 vai a sala da equipe médica e ao mostrar o exame, explica o histórico da paciente enquanto médica olha o exame. Médica diz que com 23 semanas de gestação não tem indicação para fazer o CTG e pergunta quem solicitou o exame, R3 diz não saber e que apenas fez, pois preceptora a pediu. Então médica explica a R3 quais são as indicações e o motivo pelo qual não se pede CTG antes de 26 semanas de gestação, neste momento R3 escuta atentamente a fala da médica. R3 sem graça perante a situação, ao final da fala da médica, R3 diz que sabe as indicações do CTG, mas que ao realizar não pensou no caso do paciente, apenas executou o foi solicitado.

				Admite paciente em pós-operatório imediato de curetagem, orienta em relação ao funcionamento do setor, sobre a permanência do acompanhante, horário para liberar dieta e sobre alta após 6 horas de observação, caso permaneça estável. Em seguida, realiza uma breve entrevista para entender como procedeu o aborto e ao final avalia se paciente está tendo sangramento, diz a paciente que não há sangramento.
				TE comunica que paciente 4 está iniciando com sintomas de abstinência e se irá antecipar a medicação prescrita. R3 para por uns instantes, eleva sobrancelhas, lê prescrição médica e depois pergunta a TE o que ela acha, TE opina e ao final R3 diz para não administrar, justificando que paciente já recebeu uma dose da medicação e que é melhor observar.
				TE que está aferindo os sinais vitais da paciente admitida, comunica pressão de 80x50 mmHg, R3 que se encontra no posto de enfermagem vai em direção ao box e ao chegar, percebe que o tamanho do manguito está desproporcional a paciente e diz "esse manguito é o de obeso, melhor usar o menor, a paciente é magra...". R3 sai do box.
				Ao sair do box, passa pela paciente 3 e lembra que não retirou o aparelho de CTG da paciente. Então, entra no box, retira CTG e auxilia paciente ir ao banheiro.
				Retorna para o posto de enfermagem e visualiza os resultados de exames da paciente 3 na bancada, então avalia os resultados e vai ao PA para discutir resultados de exames com a enfermeira. Passa detalhes do caso clínico da paciente 3 para a enfermeira, fala sobre os resultados de exames, diz ser necessário solicitar novo exame de sangue, pois a amostra coagulou e ao final diz "acho que está caminhando para síndrome de help". Enfermeira concorda com R3 e orienta a mesma solicitar novos exames com urgência. Sai do PA e vai a sala da equipe médica e comunica resultados dos exames.
				Retorna para o setor e TE diz que chamaram paciente x para realização de US, então auxilia TE preparar paciente, TE diz estar com muita demanda e que não consegue descer com a paciente naquele momento, então R3 solicita residente médica para descer sozinha com a paciente para exame.
				Retorna para a bancada do posto de enfermagem e evoluiu resultados de exames da paciente 3 em prontuário, neste momento TE diz que acabou de coletar nova amostra de sangue da paciente 3, então R3 pede a ela para comunicar ao laboratório a urgência na emissão dos resultados. Após finalizar sua evolução, conversa com paciente 3 e acompanhante sobre o risco de a paciente estar evoluindo para Síndrome de Help e risco de evolução para aborto.
				Ao retornar para o posto de enfermagem inicia solicitação de exame de plaquetas, pede ajuda para TE, pois não sabe qual é o impresso correto para fazer a solicitação de exame. TE mostra qual é o impresso e começa a preencher. No meio do preenchimento, levanta estressada e se direciona para a sala da equipe médica e diz para algum médico solicitar o exame, pois está muito apertada e com muitas demandas para resolver.
				Ao retornar para o setor, se depara com a enfermeira da noite chegando para pegar o plantão, R3 diz que preceptora saiu para um transporte de paciente que ainda não voltou e diz que está com muita demanda ainda para resolver. Fala para a enfermeira que paciente 1 não urina desde a sua primeira cirurgia do dia, que naquele momento tinha completado 9 horas sem urinar, então seria necessário passar um CVD. Enfermeira pergunta o motivo da admissão, R3 diz ser hemorragia, enfermeira então responde que a paciente deveria ter sido sondada desde a primeira cirurgia do dia e que não era para ter esperado 9 horas para se passar a sonda. R3 então responde que não sabia sobre a existência do protocolo de CVD e também não sabia que paciente com hemorragia é indicação para sondagem.

				Enfermeira meio sem paciência começa a remanejar os pacientes de leito de forma que os mais graves fiquem próximos ao posto de enfermagem. Em alguns momentos cobra a passagem de plantão da residente, a mesma diz que irá atualizar passômetro para passar os casos, diz ainda ter muita coisa para evoluir. R3 atende o telefone, recebe e anota os resultados de exame da paciente 3, vai a sala da equipe médica e passa os resultados para a médica, retorna para o posto e continua sua evolução. Médica chega e começa a conversar com a enfermeira da noite sobre os próximos horários de solicitação de coletas de exames da paciente 3, dizem que daqui 6 horas precisa repetir a coleta, R3 então pergunta o motivo de repetir daqui 6 horas, em seguida diz "só para eu entender o motivo". Médica explica e R3 fica atenta olhando para a médica e concordando com a fala.
				Enfermeira do noturno pergunta a R3 se passou CVD em paciente 3, R3 diz que não. Enfermeira então, diz que paciente grave, evoluído para síndrome de help é necessário passar CVD, diz que o primeiro órgão a ser acometido é o rim e que tem certeza de que paciente não urinou durante a tarde, R3 concorda com enfermeira e diz "realmente não urinou desde cedo". R3 faz uma cara de chateada. Enfermeira se senta ao lado de R3 e explica a fisiopatologia da síndrome de help e os cuidados de enfermagem para a R3, que escuta com atenção, olhando para os olhos da enfermeira e concordando com a cabeça. Ao final, enfermeira diz "então, o que uma enfermeira obstetra precisa fazer nesta situação?" e então, enfermeira explica todas as condutas que R3 tinha que ter realizado. R3 diz que era o seu segundo dia no setor, que a sua preceptora estava em transporte externo e que tudo que estava acontecendo no plantão, ela estava discutindo com a enfermeira que estava como referência para ela no PA e que também estava passando tudo para a equipe médica, mas que a equipe médica não tinha tomado nenhuma conduta até o momento de sua chegada ao plantão.
23/09/2022	14:00	18:00	Ambulatório Saúde da Mulher	R3 chama paciente ao seu consultório e na ficha da paciente vê que é uma revisão do DIU, então inicia uma breve entrevista e à medida que escuta paciente falando, vai registrando. Ao final, solicita paciente para colocar camisola e se deitar na maca, enquanto isso R3 lê histórico da paciente.
				Inicialmente, faz o toque e explica para paciente que irá inserir o espéculo, ao inserir diz a paciente que está visualizando o fio do DIU. Ao medir, diz que está com 1,5 cm de fio, então explica que está bem-posicionado e que a partir daquele momento, pode suspender o anticoncepcional. Retira o espéculo e paciente vai ao banheiro se trocar. Enquanto isso, evolui o procedimento, em seguida, orienta paciente sobre a próxima revisão do DIU dizendo que pode fazê-la em um posto de saúde ou em consulta particular.
				Ao finalizar a consulta, R3 vai ao posto de enfermagem e pega nova ficha e diz "outra revisão de DIU". Então chega à sala de espera e chama pelo nome da paciente. Entram no consultório e R3 pergunta como a paciente passou após a inserção do DIU, paciente responde que está ótima e que não sentiu nada. R3 pede a ela para resumir sua experiência em uma palavra, paciente responde e R3 registra. Pergunta a data de sua última menstruação, paciente responde ter parado de tomar anticoncepcional a 2 semanas, então R3 faz as contas de quantas relações sexuais paciente teve sem o anticoncepcional e orienta sobre a importância de usar camisinha. Pergunta sobre o fluxo menstrual, duração da menstruação e se tentou tocar o fio do DIU. Paciente prolixa, R3 não consegue encurtar as respostas da paciente o que faz a entrevista ser bem demorada.
				Solicita a paciente a colocar a camisola e se deitar na maca. Faz o toque, paciente sente muita dor, então para o procedimento e pergunta o motivo da dor, paciente responde ter problema de lubrificação. R3 utiliza o lubrificante e faz o toque. Insere o espéculo e muda várias vezes sua postura corporal na tentativa de visualizar o fio do DIU. Pega o foco de luz e o posiciona, não resolve, então explica para paciente que irá passar uma gaze no local na tentativa de facilitar a visualização. Após um bom tempo tentando visualizar o fio, R3 o visualiza e faz a medição. Diz que finalizou e que pode trocar de roupa. Enquanto isso, R3 registra o procedimento. Em seguida, orienta paciente sobre as próximas revisões, sobre DST e explica as variações no tamanho do fio.

29/09/2022	13:00	19:00	Centro de Parto Normal	R3 preenche formulário de admissão de puérpera.
				Enfermeira chama R3 para auxiliá-la na sutura de parturiente. Busca materiais, os organiza em mesa de procedimento e auxilia a enfermeira, entregando os materiais a ela. Enquanto realiza sutura, R3 observa atentamente a técnica desenvolvida pela enfermeira e ao final diz em voz alta ter sido a primeira vez que tinha visto uma sutura.
				Solicita acompanhante a fazer a internação da parturiente e explica os documentos necessários. Em seguida organiza o prontuário da paciente em ordem cronológica.
				Enfermeira solicita R3 a fazer prescrição para dor, R3 prescreve medicações sem dificuldade.
				Realiza exame físico da parturiente, avalia mama, abdome, retração uterina e panturrilha. Orienta em relação aos cuidados do RN.
				Em seguida, registra o exame físico. Liga para o laboratório para solicitar resultado de COVID e HIV da parturiente, resultados negativos.
				R3 mostra sua evolução para a preceptora que lê e comenta. Preceptora diz que a evolução está boa e completa, ressalta a importância de escrever os resultados de exames e testes rápidos para COVID e HIV, como a R3 fez. Orienta R3 a escrever os sinais vitais da parturiente, explica a relevância destes dados na evolução, R3 diz "nossa... verdade, é importante mesmo!" agradece sua preceptora, vai a parturiente checa os sinais vitais e em seguida os registram em prontuário.
				R3 fala com a preceptora que tem dificuldades para interpretar os resultados de coagulação sanguínea, então preceptora abre o protocolo de tromboembolismo da instituição e vai explicando todas as etapas, à medida que a preceptora fala, R3 faz anotações em seu caderno. Ao final, R3 pega os resultados de exame da parturiente e vai explicando a partir destes resultados, o que entendeu do protocolo para a preceptora.
				TE comunica temperatura do RN de 34,3°, R3 orienta TE colocar RN no berço aquecido.
				R3 realiza exame físico no RN, verifica frequência respiratória, temperatura e realizar testes de reflexos. À medida que realiza os testes, vai explicando para a mãe as reações que o RN vai tendo. Ao final, evolui o RN.
30/11/2022	08:00	12:00	Auditório	Pautas: Combate ao incêndio; Falta de uniformes na instituição, comunica a retirada de uniformes danificados; Desabamento do telhado na Un. X, devido a chuva, informa o valor gasto de forma emergencial; Convite para a comunidade da Unidade de Análise 2 participar do grupo de conversa "Comunidade Preta"; Informa a repercussão positiva sobre a divulgação da inserção do DIU na Un. X; Representantes profissionais e da comunidade expuseram suas vivências no Congresso Abrasco que aconteceu em Salvador; Informa o aumento da COVID-19 no Brasil e solicita reforço em relação as medidas de prevenção e uso de máscara. Durante toda a reunião, R3 mexe no celular, visualiza seu Instagram por vários momentos; pega sua agenda pessoal e a organiza, incluindo e excluindo plantões da residência; conversa assuntos aleatórios e faz comentários superficiais sobre alguma pauta informada com sua colega de residência que está sentada ao seu lado.
				Psicóloga pergunta para as residentes quais são os instrumentos de trabalho que utilizam no dia a dia da residência. R3 participativa na interação, diz que utiliza a voz como instrumento de trabalho, psicóloga elogia sua resposta e R3 sorri. R3 com uma interação ativa durante toda a aula. Assentada confortavelmente na carteira, costas encostadas e pernas cruzadas. No decorrer da aula, R3 faz associações das falas da psicóloga com o seu dia a dia na residência e em vários momentos conta exemplos de diferentes tipos de comunicações que ela já vivenciou durante suas atividades práticas. Ao mesmo tempo que outras alunas também fazem o mesmo, R3 escuta com atenção e ao final ri sobre o exemplo contado; ou concorda com a fala,

				balançando a cabeça; ou diz alguma palavra em voz baixa, respondendo a indagação ou complementando a ideia expressa pela colega. R3 estica suas pernas, mantendo encostada. R3 traz um conhecimento da aula teórica para o seu comentário: na aula de pediatria aprendemos a importância de conversar com o paciente na mesma altura em que ele se encontra. Ontem, em um desfecho ruim de uma paciente, eu presenciei residentes, médicos, enfermeiros e doula, todos de pé ao redor da paciente, e a paciente alí (faz o gesto abrindo os braços e as penas, no sentido de estar ali deitada, exposta) .... muito ruim. R3 transmite a mensagem corporal que está bem à vontade na aula, interage e ri. À medida que a aula decorre, R3 faz comentários sobre assuntos da aula, cenas do seu dia a dia que associam com a fala da psicóloga, com a tutora que está ao seu lado ou então com a sua colega residente que está sentada do outro lado. Atenta a fala da psicóloga a todo o momento. Não utiliza o celular durante a aula. Psicóloga lê uma frase dita pelo Papa Francisco que está escrita no slide e depois pergunta para as residentes o que entenderam da fala, R3 explica sua interpretação sobre a frase. Em seguida passa para o próximo slide onde está escrito a palavra descomunicação (a frase do Papa Francisco é sobre a descomunicação). R3 conta um episódio de sua vivência com a descomunicação. Outra residente conta uma outra vivência, durante a fala da colega, R3 em 2 momentos complementa com palavras que a residente não encontrava/lembrava durante a sua fala. Psicóloga passa um vídeo sobre a importância da comunicação no trabalho em equipe, ao final R3 diz para a turma a frase que mais gostou do vídeo e explica o motivo, finaliza associando com a prática dos residentes, com a rotina de trabalho e carga horária extensa dos alunos.
				Primeira dinâmica: parte da aula em que é discutido os tipos de comunicação com os pacientes e colegas de trabalho, comunicação verbal e não verbal. Psicóloga realiza a dinâmica de telefone sem fio. R3 fica assistindo duas residentes fazer o teste do telefone sem fio com dois copos de plásticos conectados por um barbante. Acha engraçado, sorri e faz comentários engraçados.
				Segunda dinâmica: a psicóloga solicita a todas para ficarem de pé, formando um círculo e em seguida, pensarem em sua música preferida. R3 fica de pé, coloca suas mãos na cintura e fecha os olhos Psicóloga então diz para todas começarem a cantar sua música preferida ao mesmo tempo, enquanto isso, ela iria escutar e depois dizer qual era a música predileta de cada uma. Todas cantam sua música ao mesmo tempo e ao final psicóloga diz: não entendi nada. Moral da dinâmica: todas emitiram suas mensagens, no sentido de dizer qual a sua música predileta, mas a comunicação não foi efetiva, pois todas falaram ao mesmo tempo e com isso a psicóloga não escutou nada. R3 bem envolvida na dinâmica, ao final diz a tutora ao seu lado "isso acontece demais, né.... achamos que as pessoas estão entendendo o que estamos dizendo".
				Ao iniciar a aula sobre submissão de projetos de pesquisa na Plataforma Brasil, R3 assina lista de presença e inicia o preenchimento de folha de solicitação de férias. Ao finalizar o preenchimento, para e fica olhando de forma vaga para os slides, tira foto de dois slides. Pega seu caderno, escreve seu planejamento e organização dos seus plantões da residência para o mês de dezembro. Ao finalizar, preenche impresso de lista de presença das atividades práticas do mês de novembro. Escreve em cada dia do mês, o setor em que estava e depois assina. Bibliotecária faz perguntas para a turma, mas R3 parece nem escutar, continua focada no impresso de lista de presença. Ao finalizar o preenchimento, pega sua agenda pessoal e confere o que escreveu na lista de presença com os plantões planejados na agenda. Não interage na aula, não presta atenção na aula. Alunos mais dispersos com poucas perguntas.<br>Ao finalizar o preenchimento, coloca seus cotovelos na carteira e deita sua cabeça no braço e fica olhando de forma vaga para os slides. Olha o Instagram, escreve um recadinho para a colega residente e entrega para ela, colega responde e devolve para R3. Bibliotecária, ao finalizar a aula expositiva, abre o site da Plataforma Brasil e faz o passo a passo de uma submissão de projeto de pesquisa, enquanto isso R3 fica mexendo no celular. R3 aérea durante a aula. Sai da sala para buscar água e entregar lista de presença na secretaria, retorna em menos de 5 minutos. Ao final, professora pergunta se tem alguma dúvida e R3 diz: "mas essa submissão é no final de tudo, né? Quando eu finalizar o projeto de pesquisa!"

04/12/2022	07:00	12:30	Unidade Gestante de Alto Risco	Transferência de cuidados no posto de enfermagem, profissionais presentes: enfermeira do noturno, enfermeira do diurno, R3 e técnica de enfermagem. R3 pega impresso com os casos dos pacientes e acompanha a passagem de plantão. Em alguns momentos anota informações e destaca com caneta marca texto pontos relevantes da transferência de cuidados. Médica chegam, durante a transferência e ficam ouvindo a passagem dos casos. Ao final, médica solicita passar os casos de 3 pacientes que elas não escutaram. Finalizado a transferência, R3 lê todo o impresso e continua destacando partes relevantes.
				Preceptora chama a R3 para dividirem os pacientes do plantão. Neste momento, R3 diz que pelo fato de a enfermeira preceptora ser intensivista, ela como residente obstétrica, teria que avaliar os 3 RNs que estavam na unidade, então deixou a entender que ficou com muitos pacientes para avaliar. Preceptora então, diz que avaliaria uma puérpera a mais.
				R3 chega no box e diz para a mãe que irá avaliar o RN 1, então retira o RN do colo da mãe e coloca no berço. Inicia o exame físico, afere temperatura, frequência cardíaca e respiratória, ausculta, avalia coloração, céu da boca e cabeça. Faz vários testes de reflexo. Pergunta para a mãe se está amamentando, mãe diz que sim, mas diz que o RN está cuspindo um líquido transparente, R3 explica o motivo, diz que se o RN iniciar com engasgos ou náuseas tem a possibilidade de passar uma sonda gástrica para drenagem. Acompanhante pergunta se mãe realmente está produzindo leite, pois o RN chorou a noite toda. R3 diz que nos primeiros 3 dias é produzido o colostro e que isso alimenta o RN, mas diz que irá solicitar avaliação da pediatra. Diz sobre a possibilidade do próprio líquido que o RN está cuspindo estar incomodando o RN. R3 busca balança, retira roupa do RN e o pesa. Ao final diz que irá procurar pediatra e conversar com a mesma.
				R3 inicia evolução, para isso busca o prontuário da mãe e do RN, faz contas no celular para definir estatura e peso do RN. À medida que lê as informações em prontuário, vai construindo sua evolução. Ao finalizar evolução, retorna no box e diz para a mãe que de acordo com a evolução da pediatra em prontuário, o RN tem proposta de receber uma dose de penicilina para tratamento de VDRL e em seguida receberá alta.
				Sai do box e procura pediatra pelo hospital, ao encontrar solicita avaliação para o RN 1 para que possa liberar a alta da mãe e RN 1. Pediatra diz ser a única no plantão e que tem muitas urgências para atender e que provavelmente não passará na UGAR para avaliar o RN. Ainda assim, R3 passa o caso do RN para a pediatra e a mesma diz que vai ler o caso no sistema e prescrever a penicilina. R3 ao retornar para UGAR, comenta a situação com a preceptora.
				Preceptora chama R3 para vê-la avaliar uma paciente que está com proposta de receber transfusão sanguínea. R3 interessada em acompanhar, já que nunca teve esta experiência. Preceptora entrevista paciente, neste instante R3 se aproxima da paciente para escutar suas respostas, paciente fraca, falando baixo. Ao fazer exame físico, percebe que sonda Foley está dobrada, então mostra para a R3 e explica qual o melhor posicionamento e fixação do sistema de CVD a fim de prevenir que a sonda dobre. R3 então se aproxima da preceptora para visualizar como a sonda está posicionada. Preceptora solicita R3 aferir a frequência respiratória. Preceptora e médica diz a paciente que precisa aferir a pressão da paciente em dois momentos, sentada e em pé, então iniciam a manipulação da paciente. Enquanto isso, R3 observa com as mãos na cintura. Na manipulação, preceptora diz "R3 olha a bolsa aqui, àquela hora tinha 150 ml de diurese, depois que desdobramos, foi para 450 ml, temos que ficar atentas a essas sondas nas pacientes!". Durante a fala, R3 agacha, espantada com a fala da preceptora, para visualizar o volume e diz assustada "nossa é mesmo... temos que ficar atentas!".
				Ao finalizar a observação com a preceptora, R3 pega o prontuário da paciente que está com proposta de receber transfusão e começa a ler, preceptora passa ao seu lado e R3 diz "estou aqui procurando se essa paciente já recebeu algum volume". Preceptora diz, hemoglobina de 8, acho que podemos tratar de outras maneiras, antes de pensar na transfusão. Preceptora sai e R3 continua lendo, ao final, R3 diz "talvez ela nem precise de bolsa de sangue", levanta-se e vai até a preceptora e diz "paciente recebeu 500 ml de soro na madrugada". Preceptora fala com a residente que talvez seja indicado prescrever noripurum, R3 concorda.

				R3 avalia RN gemelar, inicia exame físico pelo RN 2, afere temperatura, frequência cardíaca e respiratória, ausculta, avalia coloração, céu da boca e cabeça e faz vários testes de reflexo. Conversa com o pai sobre as crianças. Ao finalizar, diz a mãe que irá confirmar a necessidade de pesar a RN 2 e 3, pois ainda não deu 24h de vida. Então, R3 vai a unidade de pré-parto perguntar para enfermeira obstétrica, diz que não precisa.
				Sai da Unidade de Pré-parto e vai para a Unidade de Urgência e Emergência, chega à unidade e ao encontrar outra enfermeira obstétrica, diz que precisa discutir um caso de um RN. Então R3 conta toda a história do RN 1 para a enfermeira, diz que o bebê está cuspindo um líquido transparente, diz que paciente está com proposta de receber uma dose de penicilina, devido ao VDRL. Enfermeira diz que prefere ir avaliar o RN, ao caminharem para a UGAR, vão conversando sobre o caso clínico do RN. R3 diz a enfermeira que nunca viu tratar VDRL apenas com uma dose de penicilina, enfermeira diz "verdade, eu também não". Chegam ao box do RN, R3 pega o bebê e enfermeira faz o exame físico, à medida que enfermeira avalia, R3 continua falando informações sobre o RN 1. Enfermeira diz que R3 pode liberar a alta do RN após receber a dose de penicilina. Enfermeira sai e R3 explica a conduta para mãe e acompanhante.
				R3 avalia o segundo RN gemelar, inicia exame físico, afere temperatura, frequência cardíaca e respiratória, ausculta, avalia coloração, céu da boca e cabeça e faz vários testes de reflexo. Durante a avaliação a vó chega e R3 explica toda a situação clínica da mãe e dos bebês. Orienta a mãe sobre a pega adequada para a amamentação.
				R3 retorna do café e já inicia a evolução dos RNs gemelares. Faz leitura do prontuário e registros no sistema eletrônico.
				Durante os registros e leitura do prontuário, R3 conta para a preceptora que semana passada ao analisar diversos resultados de exames de um determinado paciente, deixou passar um resultado de VDRL positivo. R3 diz que foi sua colega residente que contou para ela que não tinha visualizado o exame, diz ter ido embora para casa triste com a situação, mas que agora, à medida que avalia os resultados de exames, vai destacando de caneta marca texto o que já analisou, assim diminuiu o risco de deixar passar alguns resultados.
				TE comenta com R3 que o RN 2 se engasgou com a saliva e os pais assustaram muito, pois o bebê chegou a mudar a coloração da pele. R3 orienta a TE a chamar os pais e orientar como proceder durante estas situações. R3 diz para a TE mostrar para eles como executa a técnica de engasgo e orienta evoluir tal conduta.
				TE comunica temperatura corporal de RN 3, diz que aqueceu com cobertores e roupinhas, mas não melhorou. R3 orienta colocar o bebê em contato pele a pele com a mãe.

Fonte: Elaborado pelas autoras.

O quadro 4 apresenta as descrições das atividades desenvolvidas pelo Residente 4 (R4) do primeiro ano do PRAPS Modalidade Uniprofissional Intensivismo, Urgência e Trauma em 30 horas de observação.

Quadro 4 - Descrição das atividades desenvolvidas pelo R4 em 30 horas de observação.

**Data Observação**	**Horário Início**	**Horário Término**	**Setor Observação**	**Descrição das atividades realizadas pelos residentes**

04/04/2023	13:30	19:30	Sala de Emergência Clínica	R4 organiza paciente para ser transferido para outra sala de emergência, retira monitorização e desconecta medicações em infusão, TE encaminha paciente.
				R4 permanece no leito fazendo desinfecção de monitor e cabos de monitorização.
				Paciente chama R4 e solicita ajuda com CVP. Infusão de soroterapia finalizou, R4 saliniza CVP e sai do leito.
				TE transfere paciente para outra sala de emergência. R4 vai ao box e faz desinfecção de monitor e cabos de monitorização. Retira cama do box e coloca no corredor, preceptora diz a R4 para chamar a higienização para finalizar a limpeza do box e cama.
				Paciente chama R4 e comunica que medicação finalizou, R4 organiza material e saliniza CVP.
				Preenche passômetro dos enfermeiros com informações de acessos periféricos e propostas de exames.
				Passa por um paciente, para, começa a organizar o leito do paciente e eleva grades da maca. Orienta paciente a solicitar marreco, para parar de utilizar o coletor urinário. Prepara materiais para troca de fralda e com auxílio da TE realiza higienização do paciente e troca de fralda, ao final guarda biombo, recolhe roupa de cama e coloca no dispenser.
				Preceptora comunica que haverá uma admissão e fala sobre o caso do paciente de forma superficial e objetiva. R4 organiza leito para receber a admissão
				Realiza desinfecção das lâminas de laringo e em seguida as envolve com filme transparente.
				Bomba de infusão apita, medicação finalizada, olha prescrição médica, não entende se é para iniciar novo soro fisiológico, então pergunta para a TE que diz ser o último frasco. Vai ao paciente e saliniza CVP.
				Preceptora comunica a R4 que paciente será transferido, R4 então comunica paciente sobre a transferência de setor e inicia a organização para o transporte, em seguida com auxílio da TE encaminha paciente para a sala de observação.
				Realiza transferência de cuidados para a enfermeira responsável pela sala, R4 diz o diagnóstico e onde está o CVP e sai da sala de observação.
				Retorna para o leito vago e realiza higienização do monitor e cabos de monitorização.
				R4 ajuda TE a transferir paciente para outra cama, em seguida organiza paciente no leito.
				Preenche passômetro dos enfermeiros.
				Ajuda TE a fazer a higienização do box.
				Admite paciente, monitoriza, verifica pressão arterial e faz um breve exame físico no paciente.
				Inicia punção de CVP, ao procurar veia para puncionar, TE orienta com dicas para facilitar a punção. R4 fica atento as orientações e direcionando seu olhar para o dedo da TE que vai mostrando as veias do braço do paciente, explicando as características e dizendo quais são as melhores veias para puncionar. Em seguida, R4 consegue puncionar a veia, faz os testes de verificação de retorno venoso e fixa CVP.
				Auxilia TE a realizar ECG no paciente.
				Vai para o computador e realiza a admissão do paciente.
				Organiza paciente para a tomografia, com auxílio da TE encaminha paciente para o exame, ao retornar para o setor, após colocar paciente no box, TE explica para R4 que pacientes graves precisam ficar próximos ao carrinho de emergência. Assim, R4 coloca paciente mais afastado do carrinho de emergência e o monitoriza com ECG, PNI e oxímetro.

				Vai para o computador e inicia evolução de enfermagem.
				Preceptora solicita R4 para buscar materiais para sondagem na farmácia, TE aproveita e solicita buscar 6UI de insulina regular. R4 vai a farmácia e busca tudo o que foi solicitado. Entrega insulina para TE e organiza mesa com materiais para a sondagem para a preceptora fazer o procedimento. TE chama R4 e pergunta para ele quantas unidades de insulina que ele pegou, R4 responde 6UI, então TE mostra para ele que ele aspirou 60UI e mostra na seringa de 1ml as graduações, o ensinando o que é 60UI e o que é 6UI. R4 então diz "nossa, 6UI é pouco demais".
				Pega prancheta com o passômetro de enfermagem e vai passando nos pacientes atualizando nome, data de nascimentos, locais de acesso e medicações que estão infundindo.
				Avalia paciente, faz entrevista com o mesmo, exame físico, pergunta se sente dor e avalia MMII.
				Vai para o computador e inicia evolução de enfermagem. Durante suas evoluções, R4 analisa resultado de tomografia, evoluções médicas e lê prescrição médica. R4 fica um bom tempo procurando o prontuário do paciente que foi admitido. Vai em várias salas do Pronto Atendimento, pergunta para o enfermeiro que levou o paciente na admissão e depois de um bom tempo encontra o prontuário.
				Retorna para a evolução de enfermagem e finaliza o preenchimento do passômetro dos enfermeiros. Preceptora chega e comenta com R4 sobre a forma como se preenche o mapa transfusional e como deve proceder nas situações em que passa o plantão com uma transfusão em andamento. R4 ao receber a orientação, continua escrevendo sua evolução e ao final diz ok para a preceptora.
				Paciente que se encontra em VNI, chama R4, ventilador mecânico apita, R4 fica olhando tentando resolver, movimenta máscara, sem sucesso, sai do leito e deixa ventilador mecânico apitando.
				Enfermeira do plantão noturno chega e preceptora inicia a transferência de cuidados, R4 fica escutando, passa o caso de um paciente que admitiu e no restante dos pacientes, R4 fica escutando e em alguns momentos complementa a fala da enfermeira.
06/04/2023	07:00	13:00	Sala de Emergência Clínica	Início da transferência de cuidados, preceptora e enfermeira do noturno vão passando em cada leito e fazendo a passagem de plantão, R4 fica ao lado da preceptora escutando e em alguns momentos se aproxima da enfermeira da noite para escutar o que ela está falando. Ambiente com muito barulho.
				Realiza o teste do desfibrilador, ao final vai a sala da supervisão de enfermagem e cola impresso do teste no caderno específico.
				Auxilia a acadêmica de enfermagem a preencher o checklist de aparelhos presentes na unidade. Explica de forma rápida o impresso para a acadêmica.
				Olha em todos os armários e gavetas a quantidade de capotes, almotolias e kits de punções, em seguida vai ao arsenal buscar o que está faltando na unidade.
				Pega impresso de controle de paciente internados na unidade e começa a anotar o nome completo de todos os pacientes e n° do leito. Verifica se todos os pacientes estão com pulseira de identificação.
				Avalia paciente 1, faz exame físico, ausculta e pergunta sobre dor, diurese, fezes e se está com falta de ar.
				Preceptora comunica a R4 sobre a deficiência visual de uma paciente internada na sala e orienta como proceder. R4 vai escutando e caminhando em direção ao próximo paciente que fará avaliação.
				Avalia paciente 2, faz exame físico, anota as medicações que estão em infusão, mensura a FR, ausculta e pergunta se paciente está com dor.

				Atende o telefone e recebe comunicado que tem planejado 5 pacientes para fazer ECO, comunica para a preceptora e planejam os transportes.
				Organiza paciente para fazer o ECO, desconecta medicações e monitor, solicita acompanhante para auxiliá-lo no transporte da paciente, chega a Unidade do exame e deixa paciente na fila, sendo acompanhado pelo familiar. Retorna para a sala de emergência.
				TE pergunta a R4 o diagnóstico do paciente, R4 entrega o passômetro para a TE ler.
				Avalia paciente 3, pergunta sobre dor, se acesso periférico dói, diurese, fezes, alimentação, realiza ausculta e verifica perfusão. Durante o exame físico, paciente relata estar com vontade de urinar, mas não consegue urinar na fralda. R4 coloca comadre e fica um tempo aguardando, paciente não consegue urinar na comadre, então R4 diz que vai passar uma sonda. R4 então comenta com a preceptora que o orienta, inicialmente, estimular esta paciente, explica alguns métodos que R4 pode colocar em prática no intuito de estimular a diurese. Uma das orientações é sempre entrar com uma TE nestes momentos, por ser homem pode inibir a paciente. Orienta oferecer conforto. Então, R4 vai em direção a TE e a orienta em realizar as técnicas ensinadas pela preceptora.
				Inicia os registros de enfermagem, avalia TC de tórax do paciente que está indo para o bloco cirúrgico, mostra a imagem para a preceptora e confirma com ela se a imagem que está vendo é um empiema no pulmão. Preceptora confirma e diz, "empiema é pus". R4 então, lê evolução médica para entender a história do paciente. Em seguida inicia sua evolução de enfermagem.
				Médica solicita R4 para chamar o laboratório, preceptora que está ao lado diz a médica que ela pode ligar chamando o laboratório, então médica faz a ligação e sai da sala. Preceptora então diz a R4 que esses tipos de demandas, os próprios médicos podem fazer. Orienta a R4 a focar nas atividades da enfermagem e não ficar atendendo a pedidos que o faz perder o foco.
				R4 retorna para a evolução dos pacientes. Fica muito tempo fazendo os registros, além disso, lê evoluções de outros profissionais. Em alguns momentos, levanta-se para ver uma medicação que está infundindo, local de acesso periférico e local onde está a pulseira de identificação.
				Ao final, vai na paciente 3 e verifica se a mesma urinou. Avalia bexigoma e diz a preceptora que paciente não urinou e não tem sinal de bexigoma. Preceptora orienta acompanhar.
				Preceptora solicita a R4 fazer ECG em uma paciente que acabou de ser transferida para a sala amarela. Paciente encontra-se deambulando no corredor, o que faz R4 gastar muito tempo procurando a paciente na unidade. Ao encontrá-la auxilia paciente a retornar para o seu leito, explica a necessidade do fazer o ECG. Deita paciente na maca. Sai para buscar ECG, gasta um bom tempo tentando encontrar, inicia ECG, conta com cuidado os espaços intercostais para conectar os eletrodos. Ao final, mostra resultado para a médica e monitoriza paciente no monitor multiparâmetro.
				Oferece café ao paciente, vai dando aos poucos ao paciente que se encontra com dificuldade para movimentar os braços.
				Transporta paciente que está no box da urgência para o box 5, desmonitoriza para fazer a transferência e em seguida monitoriza novamente. Bomba de infusão apita acusando ar na linha, fica um bom tempo tentando retirar o ar do equipo.
				Avalia paciente que acabou de dar entrada na sala, anota invasões, sinais vitais e faz exame físico do paciente.
				Inicia os registros de enfermagem.
11/04/2023	13:30	19:30	Sala de Emergência Cirúrgica	Realiza o teste de 03 desfibriladores, ao final vai a sala da supervisão de enfermagem e cola impresso do teste no caderno específico.

				Retorna para a sala de emergência, preceptora solicita a R4 para finalizar as visitas. R4 então, vai passando nos leitos e comunicando que o horário de visita finalizou.
				Passa pelo corredor do Pronto Socorro e percebe paciente deitada em maca recebendo SF 0,9%, no entanto o frasco encontra-se apoiado na cama. Então começa a procurar um suporte de soro dentro da Unidade. Entra em todas as salas de emergências e fica um bom tempo procurando um suporte, ao encontrar, coloca SF pendurado no suporte e percebe que o acesso está obstruído. Então sai para buscar seringa com SF para lavar. Ao final lava acesso periférico e orienta paciente a não dobrar o braço.
				Diz a preceptora que vai fazer o curativo de um paciente, pergunta se é melhor usar óleo mineral ou petrolato, preceptora diz que não tem petrolato na instituição. Então sai da sala de emergência para procurar um frasco de óleo mineral, ao conseguir, prepara material para fazer curativo, chega ao paciente e explica o procedimento. Escoriações com muita sujidade, R4 higieniza bastante o local, aplica óleo mineral, gaze e enfaixa a coxa.
				Acompanhante chama R4 e solicita duas declarações, uma de acompanhamento e outra provando que paciente está internado. R4 solicita ao médico e depois chega ao paciente dizendo que sua acompanhante está providenciando as documentações necessárias para levar ao fórum.
				Admite paciente proveniente da UPA norte, faz exame físico, percebe que paciente está sem pulseira de identificação. Então vai a recepção para saber se paciente foi internado e solicita pulseira de identificação. Retorna e identifica o paciente.
				Vai para o computador e inicia os registros de enfermagem, descreve sobre a troca do curativo e evolui paciente que acabou de admitir. Ao final, lê evolução médica do paciente recém-admitido.
				Organiza paciente para ser encaminhado para a tomografia, paciente confuso e agitado, durante o transporte fala para paciente ficar quieto durante o exame. Ao chegar na tomografia, auxilia passar o paciente para a mesa do tomógrafo e sai para buscar outro paciente para fazer o exame.
				Chega à sala de emergência e começa a organizar outro paciente para fazer tomografia, chama a acompanhante no corredor para auxiliá-lo a levar o paciente para o exame. Ao chegar na unidade de tomografia, solicita a acompanhante para aguardar na fila com o paciente, diz que ao final do exame a equipe da tomografia retorna com eles para a sala de emergência.
				Retorna para a sala de emergência e começa monitorizar paciente que acabou de ser admitido, monitoriza paciente, reinicia infusão das medicações que são para infundir em bomba de infusão.
				Procura preceptora e pergunta se já pode levar paciente para fazer raio-x, preceptora diz que é necessário aguardar 1 hora após o paciente ser extubado.
				Profissional da Tomografia liga dizendo que paciente que levaram para fazer o exame está agitado na mesa do tomógrafo e que não foi possível fazer a tomografia. Então R4 diz a preceptora, "este paciente possivelmente está com bexigoma", então abre exame de Ultrassom do trato urinário e começa avaliar a bexiga, diz "não sei". Comenta agitação para preceptora e diz que será necessário sondar.

				Faz solicitação de materiais para fazer a sondagem, vai a farmácia buscar os materiais solicitados, ao retornar começa a organizar os materiais para fazer a sondagem de alívio. Chega no paciente, orienta sobre a sondagem e inicia a técnica de sondagem. Preceptora o auxilia durante todo o processo. À medida que executa os passos da técnica, preceptora vai explicando como deve proceder. Em alguns momentos preceptora corrige o que o R4 está fazendo e diz como deve proceder, R4 fica atento a explicação. Ao final, fica aguardando a drenagem da diurese, drenado 1300 ml de urina. Ao final recolhe materiais e troca a fralda do paciente.
				Médico inicia sondagem vesical de demora em outro paciente, técnica de enfermagem o acompanha. Médico diz que urina está da cor de água de arroz. R4 para uns instantes e fica observando o médico a passar a sonda.
				Pega o telefone e liga para a unidade de tomografia para cobrar alguns exames pendentes.
				Chega paciente baleado na sala de emergência, R4 inicia monitorização do paciente, mas médico solicita acionar onda vermelha. R4 aciona e já auxilia no transporte do paciente para o bloco cirúrgico.
				Prepara midazolam para levar paciente agitado novamente para a tomografia. Administra a dose do midazolam solicitado pelo médico, durante a infusão preceptora pergunta ao R4 se checou o acesso periférico. R4 testa o retorno venoso e em seguida continua a infusão da medicação. Ao final, encaminha paciente para a tomografia com auxílio do médico e técnico de enfermagem. Auxilia passar paciente para a mesa de exame, ao terminar a tomografia, retornam com paciente para a sala de emergência.
				Inicia evolução de enfermagem, realiza o registro da admissão do paciente baleado e outros registros de enfermagem.
				Preceptora chega com um paciente da tomografia, R4 auxilia a mesma a organizar e monitorizar paciente no leito.
				Organiza paciente para ser encaminhado para o raio-x, retira medicações que estão em infusão e monitorização. Pega um oxímetro de transporte e coloca no paciente. Encaminha paciente para o exame. Ao chegar no raio-x com o paciente, aguarda o atendimento, ao chamar o paciente, R4 auxilia colocá-lo na mesa e retorna para a sala de espera. Ao final, ajuda a passar o paciente novamente para a maca e retorna com paciente para a sala de emergência. Monitoriza e reconecta medicações no paciente. Ao final, anota sinais vitais e ausculta paciente.
				Passa em cada leito preenchendo o passômetro de enfermagem, confere o nome, data de nascimento, monitorização e invasões de cada paciente.
				Inicia evolução de enfermagem, avalia raio-x do paciente.
				Preceptora retorna para a sala de emergência, R4 passa todos os eventos que aconteceu em sua ausência. Preceptora está ajudando em uma intercorrência em outra sala de emergência.
				Chega paciente do SAMU, R4 escuta a transferência de cuidados. Paciente em convulsão, inicia tentativa de punção de CVP, não consegue. Chama preceptora que faz tentativas e também não consegue. Técnica de enfermagem depois de várias tentativas punciona acesso. Enquanto isso, R4 monitoriza paciente e organiza para passar paciente para a maca da instituição.
				R4 faz evolução de enfermagem do paciente recém-admitido.
				R4 realiza transferência de cuidados para enfermeira do plantão noturno.

20/04/2023	14:30	18:30	Sala de aula	R4 atento a tutora que inicia a conversa explicando a rede referenciamento e contra referenciamento para os residentes. Em seguida, tutora informa as pautas discutidas em reunião da Coremult que aconteceu na semana, fala sobre as disciplinas específicas e comuns, diferenças de currículos e modificações de cronogramas. R4 durante a fala da T1 abre o computador e depois fica escutando com a mão no rosto. colega residente conta sua trajetória de formação. Durante a fala da colega, R4 fica lendo slides em seu computador. Em seguida, T1 abre espaço para os residentes contarem suas experiências em campo. R4 fica em silêncio durante a fala das colegas, direciona seu olhar para as pessoas que falam e balança sua cabeça nos momentos em que concorda. T1 pergunta o que é um procedimento de emergência, R4 responde "PCR". Então, T1 explica o conceito de urgência e emergência e vias de acesso a uma urgência. Fala sobre os cuidados de enfermagem nestas situações, R4 fica olhando e balançando a cabeça em alguns momentos. Fala sobre os cuidados com CVP. R4 em alguns momentos abre a boca de sono e coça a cabeça. Colega residente conta sua experiência em assistir um paciente infartando. R4 diz "nossa... como impacta".
03/05/2023	14:00	16:00	Sala de aula	Iniciam a segunda fase do encontro de tutoria. T1 contextualiza o assunto de choque. Colega residente começa a apresentação sobre choque circulatório. Durante a apresentação, R4 fica olhando para os slides, come uma barrinha de doce e bebe água. T1 explica os mecanismos de compensação do choque e pergunta aos residentes o que é Pressão Arterial e Débito Cardíaco. R4 responde dizendo a fórmula da Pressão Arterial e do Débito Cardíaco. Em seguida, fica escutando a fala de T1 e colega residente e brinca com a garrafinha de água sobre a mesa. R4 descreve uma vivência na residência que simula ao que está sendo apresentado. Seguem para a apresentação dos demais choque pela mesma colega residente. T1 pergunta o que gera o choque cardiogênico, R4 responde de forma rápida.
				R4 apresenta estudo de caso sobre Acidente Vascular Cerebral por meio de slides. Apresentação demorada e bem detalhada sobre a história do paciente, fisiopatologia do AVC; primeiros passos do atendimento; tratamento; monitorização, diagnóstico de enfermagem e cuidados de enfermagem; etc. R4 faz sua apresentação se guiando pelos slides, demonstra domínio sobre o assunto.
				Ao final da apresentação, coordenadora elogia as informações apresentadas, mas reitera a importância de ter apresentações mais objetivas. Em seguida iniciam as discussões sobre o assunto e vários residentes descrevem vivências de seu dia a dia ao lhe dar com paciente de AVC. R4 fala sobre a dificuldade de encaminhar este paciente rapidamente para a tomografia e as conduções de cada médico.
10/05/2023	07:00	13:00	Sala de Emergência Clínica	R4 chega ao setor e já começa a acompanhar a transferência de cuidados entre preceptora da manhã e enfermeiro da noite. R4 fica escutando e escreve algumas informações em um rascunho.
				R4 começa a fazer os testes de laringoscópios e desfibrilador. Em seguida, verifica os estoques de campos, capotes, degermantes e kits de punção. Procura ECG na sala, não encontra, sai para encontrar o ECG que está sendo utilizado na sala de observação. Ao final, preenche checklist de controle de materiais e vai na sala da supervisão de enfermagem para colar o teste do desfibrilador.
				Imprime passômetro do dia e começa a passar por todos os pacientes para verificar nome completo, data de nascimento e pulseira de identificação. Vai para o computador e corrige o passômetro, em seguida imprime outra via. Ausência de folha na impressora, vai ao administrativo para buscar, demora, pois não tem folha no administrativo.
				Após conseguir a cópia do passômetro fica um tempo lendo as informações dos pacientes.
				Avalia paciente 1, faz exame físico, ausculta e palpa abdome e MMII. Anota dados vitais, dispositivos invasivos e medicações que estão em bomba de infusão. R4 fica um tempo maior neste paciente que se encontra intubado e com dispositivos invasivos

				Avalia paciente 2, faz exame físico, anota as medicações que estão em infusão, mensura a FR, ausculta e conversa com paciente sobre dor, alimentação, urina e fezes. Avalia permeabilidade de CVP. Pergunta ao paciente sobre doenças de base. Paciente conta com detalhe todo o seu histórico de doença e tratamento.
				Avalia paciente 3, faz exame físico, ausculta e palpa abdome e MMII. Anota dados vitais, dispositivos invasivos e medicações que estão em bomba de infusão. R4 fica um tempo maior neste paciente que se encontra intubado e com dispositivos invasivos.
				Paciente do leito próximo expressa sua vontade em urinar, R4 busca coletor urinário, corta uma parte do mesmo e explica para o paciente como deve utilizar. Paciente então urina e R4 despreza e anota o valor da diurese.
				R4 observa que preceptora avaliou a ferida de um paciente e pergunta se é ferida nova, preceptora explica que nem considera ferida, pois já está seca e antiga.
				Samu chega com um paciente, R4 faz uma avaliação rápida e pergunta para a preceptora se pode colocar o paciente no box 4, ela diz que sim, e R4 já organiza paciente no leito e o monitoriza.
				Inicia as evoluções de enfermagem, durante os registros abre outras evoluções de diferentes profissionais para ler.
				Técnica de enfermagem chama R4 e mostra que o CVD da paciente está com extravasamento em região uretral. R4 avalia região peri uretral e sonda, não identifica problema, mas visualiza diurese em fralda. Sai do leito e comunica a preceptora, a mesma sai para avaliar e R4 retorna para suas evoluções.
				Continua com os registros de enfermagem.
				Ao finalizar, ajuda TE a mobilizar o paciente e verificar a presença de fezes. R4 não tem dificuldades para manipular o paciente.
				Ajuda outra TE a subir paciente na cama.
				Volta para o computador e faz novos registros de enfermagem. Durante a evolução, levanta-se em alguns momentos para verificar/ confirmar posição de CVP e medicações que estão em infusão e retorna para a evolução. Ao finalizar evoluções imprime e guarda nos respectivos prontuários.
				Admite paciente, monitoriza, verifica pressão arterial e faz um breve exame físico no paciente. Anota dados vitais, nome e data de nascimento.
				Preceptora comunica ao R4 que irá puncionar PIA e CVC no paciente que acabou de chegar, assim R4 busca materiais na farmácia (kit de punção) e campos e capotes no arsenal, ao final organiza todo o material na mesa de procedimentos.
				R4 percebe que TE está com dificuldade para fazer um ECG, pois paciente está movimentando, então começa a ajudar a TE. Paciente começa a engasgar, não conseguem elevar a cabeceira do paciente, pois paciente foi colocado do lado errado na maca. Então rapidamente preceptora chega e a equipe começa a girar o colchão da maca e elevam cabeceira. Finalizam o ECG.
				R4 passa em todos os leitos atualizando os nomes dos pacientes em passômetro e confirmando as placas de identificação dos pacientes no box.
				Ajuda a equipe a transferir um paciente para maca e duas TEs levam paciente de transferência para outra unidade.

				Em seguida, R4 faz a desinfecção do box, coloca cama no corredor e procura por um bom tempo a equipe da higienização para limpar leito vago.
				Despreza e quantifica diurese de paciente.
				Admite paciente, faz exame físico. Pergunta sobre dor. Ao final solicita paciente a chamá-lo caso queira tomar o mingau.
				Prepara material para puncionar CVP, comunica paciente sobre a punção, e punciona acesso sem nenhuma dificuldade, faz fixação e identifica o acesso. Ao final vai na administração para buscar nova pulseira de identificação, retorna e coloca nova pulseira na paciente, pois a antiga estava muito apertada.
				Vai até a sala amarela e negocia um leito com a enfermeira para transferir um paciente, mas percebe que a sala está lotada, então, retorna para a sala vermelha e comunica a preceptora que não tem vaga.
				R4 ajuda a equipe a passar um paciente da maca para a cama, ajuda a posicionar paciente no leito, em seguida transferem o paciente do box 4 para o box 9. Monitoriza paciente e reinicia as medicações em bomba de infusão.
				Término de infusão da soroterapia, R4 prepara nova solução e reinicia no paciente. Percebe resistência no CVP e lava o acesso.
				R4 procura acompanhante de paciente no corredor, outra acompanhante diz que a pessoa saiu para fazer um lanche, então R4 encaminha paciente para o raio-x e fica na fila aguardando paciente ser atendida. Ao chegar na vez da paciente, R4 fica sentado aguardando término do exame. Ao final retorna com paciente para a sala.
				Passa pelos leitos atualizando os nomes dos pacientes internados no passômetro.
				Vai para o computador e inicia os registros de enfermagem.

Fonte: Elaborado pelas autoras.

O quadro 5 apresenta as descrições das atividades desenvolvidas pelo Residente 5 (R5) do segundo ano do PRAPS Modalidade Uniprofissional Enfermagem Obstétrica em 31 horas de observação.

Quadro 5 - Descrição das atividades desenvolvidas pelo R5 em 31 horas de observação.

Data Observação	Horário Início	Horário Término	Setor Observação	Descrição das atividades realizadas pelos residentes
10/07/2023	07:00	13:30	Pronto Atendimento	R5 chega ao setor com preceptora e inicia a transferência de cuidados. Enfermeira do noturno realiza a transferência de cuidados de pacientes que ficaram com pendência para o dia. Ao final ficam conversando assuntos aleatórios durante um tempo.
				**1º atendimento:** paciente entra no consultório com muita dor e chorando. R5 orienta paciente a sentar e solicita o cartão da gestante e os exames prévios. Inicia entrevista da paciente. Ao final, orienta paciente ir ao banheiro, urinar e vestir camisola. Enquanto paciente vai ao banheiro, R5 avalia os exames. Paciente se deita na maca, começa a chorar, relatando dor forte. R5 tenta acalmá-la. Passa o sonar no abdômen da paciente e fica um tempo contabilizando a FC. Em seguida coloca suas mãos

				em cima do abdome da paciente e conta as contrações. R5 explica para a paciente que fará o toque para ver quanto tem de dilatação, inicia o procedimento. Durante o toque, R5 se concentra, ficando em silêncio, em estado de alerta e com os olhos bem abertos. Durante o toque, paciente fica chorando alto. Ao final do toque e ao retirar a luva, observa o aspecto da secreção que se encontra na luva e cheira. Explica para a paciente que vai interná-la, pois está com 8 cm de dilatação. R5 solicita a TE para coletar os exames de sangue. TE chega ao consultório, faz a coleta e começa a ajudar a paciente a sentar na cadeira de rodas, em seguida encaminha paciente para a sala de parto. Enquanto isso, R5, diante do computador, faz algumas perguntas a paciente e vai anotando na evolução eletrônica. Em seguida, liga para a enfermeira da sala de parto normal e faz a transferência de cuidados. R5 segura, não demonstrando dificuldade em falar sobre a situação clínica da paciente. Ao final, pega os impressos para fazer a internação da paciente. Faz no sistema eletrônico os pedidos de exames. Enquanto preenche os impressos e solicita os pedidos de exames, R5 fica conversando assuntos aleatórios com a TE. Quando finaliza, R5 sai do consultório e vai ao consultório da médica de plantão, passa o caso da paciente, médica concorda com a conduta e assina as documentações de internação da paciente. R5 retorna ao seu consultório e preenche mais impressos de internação. Com todos os impressos em mãos, R5 vai até o setor de parto normal e entrega documentos e resultados de exames para enfermeira que está responsável pela paciente.
				**2º atendimento:** R5 chama por outra paciente, ao sentar-se no consultório, solicita caderneta da gestante e resultados de exames. R5 realiza várias perguntas sobre a gestação à medida que vai avaliando os exames. Paciente diz que sua gravidez é de alto risco e alega alergias. R5 recebe a informação com naturalidade e orienta paciente ir ao banheiro, urinar e vestir camisola. Enquanto isso, R5 faz anotações no sistema de evolução eletrônica. Paciente sai do banheiro e se deita na maca. R5 faz algumas medidas na barriga, utiliza o sonar por um tempo e ao final diz "128 bpm". Explica para paciente que irá fazer o toque, não encontra o espéculo, então sai do consultório e procura nos demais algum espéculo. Retorna para sua sala e volta a dizer para a paciente o que irá fazer. Passa o espéculo com cuidado, paciente relata dor. R5 acende o foco de luz e fica avaliando o colo uterino, preceptora entra na sala neste momento e começa a observar o colo do útero junto a R5. Ao final, preceptora diz "secreção fisiológica". R5 então começa a explicar para a paciente os diferentes aspectos da secreção à medida que a gestação evolui. Quando finaliza, começa a fazer o toque e identifica 1 cm de dilatação. R5 explica o que isso significa para a paciente. Mãe da paciente faz várias perguntas para R5, que vai respondendo com calma e segurança. Identifica a dor da paciente e diz que irá prescrever uma medicação antes de retornar para casa, mãe da paciente diz que a mesma tem alergia a muitas medicações, preceptora diz que vai prescrever prometazina, neste momento, R5 fica observando e escutando as explicações que a preceptora fornece a mãe no sentido de deixá-la segura de que a medicação não irá causar alergia na paciente. R5 analisa os exames e vai anotando os resultados no computador, diz a paciente que vai liberá-la para voltar para casa. No entanto, ao fazer a avaliação do ultrassom, identifica que o feto é CIUR, então pergunta a paciente o motivo pelo qual não fez o exame de doppler. Neste momento, preceptora diz a R5 para discutir o caso com médico. R5 vai ao setor de gestação de alto risco e discute o caso com o médico, que conversa com R5 no sentido de interromper a gestação. Assim, R5 retorna ao consultório e explica para a paciente que a definição será interromper a gestação. Paciente e sua mãe choram ao escutar a notícia sobre os riscos de um feto CIUR. R5 inicia o preenchimento dos impressos de internação. Enquanto R5 orienta paciente em relação aos TCLEs sobre o processo de indução do parto e de inserção do DIU, a preceptora vai evoluindo a paciente no sistema eletrônico. Preceptora solicita a R5 para pedir vaga na sala de indução de parto, R5 liga e faz a transferência de cuidados, R5 com domínio do caso. R5 sai do consultório, discute o caso com a médica que assina as documentações de transferência da paciente.
				**3º atendimento:** chama paciente na sala de espera, faz a entrevista com a paciente e calcula a idade gestacional. Avalia exames. Orienta paciente a deitar na maca e inicia a ausculta com o sonar. Ao final, R5 explica para a paciente sobre as dilatações, contrações, benefícios do parto normal, etc. Fica com a mão no abdômen da paciente e conta as contrações. Diz que fará o toque e inicia o procedimento, faz com rapidez e tranquilidade, ao final diz "2 cm de dilatação". Segue orientando

				como deve proceder quando a bolsa estourar, o que fazer para aliviar a dor, motivos que justificam a paciente a retornar ao hospital. Ao final das orientações, paciente vai embora e R5 fica no computador fazendo a evolução no sistema eletrônico.
				**4º atendimento:** R5 chama paciente na sala de espera, avalia idade gestacional e faz entrevista com a paciente. Em seguida, R5 sai do consultório e procura preceptora para dizer que paciente está com 40 semanas e 6 dias, pergunta como proceder. Preceptora diz a R5 para ela oferecer a paciente o descolamento de placenta. R5 retorna ao consultório, continua a entrevista com a paciente e solicita os exames prévios. Ao analisar o ultrassom, identifica que o feto é PIG, assim R5 sai do consultório novamente e procura preceptora. Ao encontrar, explica a situação para a preceptora e pergunta se acha viável pedir um doppler. Neste momento, R5, preceptora e enfermeira trocam ideia sobre qual conduta tomar. Concluem que o ideal é interromper a gravidez, mas preceptora solicita R5 discutir o caso com um médico. R5 então, vai ao médico da sala de parto e discute o caso, R5 fala com calma e segurança. Médica diz para solicitar o doppler, por segurança. R5 retorna ao seu consultório, explica para a paciente sobre a importância de fazer o doppler. Assim, liga no setor de exames, solicita a médica um encaixe para o exame e faz a transferência de cuidados para a médica que irá fazer o doppler. Em seguida, paciente se deita na maca e R5 faz o sonar. Ao final, R5 começa a evoluir, a verificar os resultados de exames e faz pedido de doppler. Ao término da consulta, paciente sai para fazer o doppler. R5 comenta com preceptora "estranho né, diante deste resultado de ultrassom não ter pedido um doppler, era importante ter pedido". Em seguida, confirma com preceptora a idade gestacional ideal para retirar um feto CIUR e um PIG.
				**5º atendimento:** chama paciente na sala de espera, faz entrevista com paciente e solicita exames. Ao avaliar ultrassons fica em dúvida sobre a idade gestacional. Chama a preceptora e mostra a ela que em cada ultrassom mostra uma data da última menstruação diferente. Então preceptora faz algumas perguntas a paciente e ao final diz para considerar a data do primeiro ultrassom, neste momento R5 fica atenta às perguntas da preceptora para a paciente e em ao final pergunta o motivo de considerar aquele primeiro ultrassom, preceptora explica. R5 dá continuidade ao atendimento e preceptora fica ao seu lado. R5 identifica uma diabetes gestacional, R5 faz as contas para ver se paciente realmente está com diabetes, preceptora ao final diz "isso mesmo, é diabetes". Preceptora diz para R5 discutir o caso com médico, R5 vai a sala de parto normal e discute o caso com o médico, que diz para solicitar um novo ultrassom. R5 retorna ao seu consultório e liga para sala de exames para solicitar o ultrassom, explica para médica que está na ligação, a importância de fazer o exame naquele momento. Médica autoriza fazer o encaixe e faz a transferência de cuidados. Então R5 explica para a paciente o motivo de fazer novos exames. Enquanto isso, preceptora foi avaliando a paciente, verificando contrações e dilatação do colo do útero. Ao final, paciente vai ao banheiro, troca de roupa e vai para a sala de medicação tomar remédio, R5 orienta paciente ir para sala de exames após tomar a medicação para dor. R5 então, evolui paciente no sistema eletrônico e preceptora vai dizendo o que identificou no exame físico da paciente.
				Enfermeira da classificação de risco chama R5 para cobrir o seu horário de almoço, assim R5 vai para a sala de classificação de risco e enfermeira explica para a R5 como usar o sistema eletrônico de classificação do paciente. R5 neste momento escuta com atenção a explicação. Enfermeira sai para o almoço e R5 fica um tempo manuseando o sistema na tentativa de conhecer e por em prática o que escutou de orientação.
				R5 faz a primeira classificação de risco, faz a entrevista do paciente de acordo com o que o sistema vai direcionando, verifica todos os sinais vitais, ao final tem dificuldade de finalizar o atendimento no sistema, TE chega à sala, percebe a dificuldade e tenta auxiliá-la. Depois de um tempo, R5 executa o que TE orientou, para por um tempo e identifica o erro que estava cometendo e consegue terminar a consulta.
				Chama a segunda paciente para classificação do risco. Faz a entrevista, verifica os sinais vitais, auxilia paciente a sentar na cadeira, paciente com muita dor. R5 registra os dados aferidos e finaliza a evolução, imprime e leva paciente para a sala de espera, em seguida coloca a ficha da paciente na frente das demais e diz "o atendimento desta aqui precisa ser rápido".

16/07/2023	07:00	19:30	Alojamento Conjunto	R5 chega ao plantão e começa a escutar a transferência de cuidados entre as duas enfermeiras do plantão diurno com a enfermeira do plantão noturno. Ambiente com barulho e em local de passagem de pessoas. R5 em alguns momentos da Transferência de cuidados conversa assuntos aleatórios com colega residente.
				1° avaliação: R5 chega no berço do RN, se apresenta e diz que vai avaliar o RN. Faz entrevista com os pais sobre o RN e exame físico minucioso. Afere FR, FC, ausculta, etc. Explica para os pais o motivo do RN estar espirrando e saindo líquido da boca. Orienta a mãe sobre a frequência da amamentação. Tira a roupa do RN e avalia as articulações, sucção, abdômen, testículos, etc. Faz curativo do coto umbilical e troca fralda devido a presença de fezes. Faz higiene no local e coloca nova fralda. Ao final, anota informações relevantes em passômetro. R5 segura durante toda a avaliação.
				2° avaliação: R5 chega no berço do RN, se apresenta e diz que vai avaliar o RN. Mãe relata dor, dificuldade para amamentar e presença de fissura na mama. R5 diz que pode tomar um remédio para dor e que vai auxiliá-la na amamentação. R5 pergunta se RN evacuou, urinou, mamou, etc. Afere FC, FR, temperatura e ausculta. Prossegue com exame físico minucioso. Presença de fezes e urina em fralda, faz a higiene e troca de fralda. Troca curativo de coto umbilical. Orienta a mãe sobre troca do curativo, assadura e demais cuidados. Ao final coloca RN no peito da mãe e a auxilia na forma correta da amamentação. Fica um tempo observando a mãe na amamentação. Ao final, faz o registro manual da evolução em passômetro. R5 segura durante toda a avaliação.
				3° avaliação: R5 chega no berço do RN, se apresenta e diz que vai avaliar o RN. Afere FC, FR, temperatura e ausculta. Orienta sobre a frequência da amamentação. Faz exame físico completo do RN. Orienta o cuidado com o coto umbilical e assadura. Explica para a mãe sobre algumas situações que é necessário retornar com o RN para o hospital. Orienta sobre cuidados para o RN não se engasgar ou sufocar. Presença de fezes e urina em fralda, faz a higiene e troca de fralda. Ao final, faz o registro manual da evolução em passômetro. R5 segura durante toda a avaliação.
				4° avaliação: R5 chega no berço do RN, se apresenta e diz que vai avaliar o RN. Orienta os pais de como colocar o RN para arrotar. RN chorando muito, R5 fica um tempo com o RN no colo. Avalia sucção, FC, FR, temperatura e ausculta. Presença de fezes e urina em fralda, faz a higiene e troca de fralda. Faz exame físico minucioso no RN. Orienta sobre o aspecto das fezes, cuidados com o coto umbilical e cuidados com a posição do RN ao colocá-lo para dormir. Ensina a mãe a vestir o RN e temperatura corporal, diz para não colocar muita roupa de frio. Ensina mãe a colocar RN no peito, fala sobre a pega e posição do RN durante a amamentação. Ao final, faz o registro manual da evolução em passômetro.
				Mãe pergunta se glicemia do RN será verificada novamente. R5 orienta a mãe sobre o motivo e a importância do controle glicêmico. Diz que fará o monitoramento da glicemia por 24h.
				Mãe chama R5 para avaliar a qualidade da pega durante a amamentação. R5 explica como mãe deve proceder, corrige a postura da mãe e posição do RN. Ensina a mãe uma técnica para estimular a protusão do bico do peito.
				Colega residente pergunta para a R5 sobre como proceder nas situações em que é necessário coletar colostro para exame. R5 explica para a colega residente o fluxo sem dificuldade.
				Vai para o computador fazer as evoluções dos 4 RN avaliados. Para a evolução utiliza a caderneta da gestante, resultados de exames e anotações do passômetro. Na evolução descreve as condutas e prescreve os cuidados. Prescreve frequência de aferição dos dados vitais e glicemia capilar. Lê evoluções da equipe multiprofissional e em alguns momentos vai na mãe do RN tirar alguma dúvida sobre o RN. Usa muito a calculadora do celular para fazer contas de horas de vida. R5 gasta muito tempo fazendo as evoluções. Ao escrever a conduta de um RN, vai até uma enfermeira e fala sobre o frênulo alterado do RN. Enfermeira orienta a solicitar avaliação com a fonoaudiologia. Então procura fono pelo hospital e não encontra. Descobre que fono não trabalha no final de semana. Então retorna para o computador e faz a solicitação de interconsulta. Finaliza as evoluções, imprime e depois organiza os impressos no prontuário.

					Orienta TE sobre um RN, diz que modificou a frequência de aferição dos sinais vitais e Glicemia capilar.
					Avalia mama da paciente, orienta sobre os cuidados para a cicatrização da fissura e diz que o RN vai passar pela consulta da fono amanhã.
					Assenta com TE e fala sobre as condutas de todos os RNs. Trocam informações sobre resultados de glicemia. R5 vai abrindo cada prontuário e discutindo com TE.
					Vai na preceptora para discutir os casos dos 4 RNs avaliados. Passa cada caso para a preceptora com detalhes. À medida que passa os casos, preceptora vai carimbando as evoluções junto com a R5. 1° discussão: Preceptora orienta a R5 solicitar o exame de toxoplasmose por segurança e explica a importância do exame.
					2° discussão: Preceptora orienta pesar o RN, pela dificuldade de amamentação e fissuras na mama da mãe, diz ser importante monitorar o peso do RN.
					3° discussão: sem comentários da preceptora.
					4° discussão: sem comentários da preceptora.
					Vai na TE e orienta sobre o que discutiu com preceptora.
					Chega para uma mãe e explica que vai pesar o RN, fala do motivo de acompanhar o peso do RN, tira sua roupinha e leva para a balança. Após pesar, coloca a roupa no RN e entrega para a mãe.
					Chega em uma paciente e solicita exames do pré-natal, verifica o resultado de toxoplasmose e depois chega para a preceptora e diz que o exame está normal.
					TE pergunta para R5 se pode administrar dipirona na paciente, R5 verifica prescrição médica e autoriza administrar.
					5° avaliação: enfermeira solicita a R5 avaliar uma mãe e RN para dar alta. R5 chega no berço do RN, se apresenta e diz que vai avaliar o RN. R5 orienta a mãe sobre amamentação, por ser o primeiro filho, detalha suas explicações para a mãe entender. Faz exame físico detalhado, afere FC, FR, temperatura e ausculta. Explica como faz curativo no coto, troca o curativo do coto e a fralda, por estar com urina. Orienta mãe sobre a frequência do banho.
					6° avaliação: avalia a mãe do RN e faz exame físico completo. Orienta sobre cuidados com a mama, regressão do útero, sangramento vaginal, uso de lenço umedecido e frequência da amamentação. Afere a saturação de oxigênio. Coloca RN no peito para avaliar a pega do RN, diz a mãe que a pega está errada. Então explica como estimular a pega correta.
					Ao finalizar exame físico, vai para a sala de evolução para ler o prontuário e evoluir. Durante a leitura detecta que paciente está com hipertensão pós-parto. Discute com enfermeira o motivo que acha que não deve dar alta na paciente, enfermeira concorda.
					Vai para o computador para fazer as 4 evoluções (2 RNs e 2 mães). Para a evolução utiliza a caderneta da gestante, resultados de exames e anotações do passômetro. Na evolução descreve as condutas e prescreve os cuidados. Prescreve frequência de aferição dos dados vitais e anota o motivo de cancelar a alta. Lê evoluções da equipe multiprofissional. R5 gasta muito tempo fazendo as evoluções. Finaliza as evoluções, imprime e depois organiza os impressos no prontuário.
					Colega residente pergunta para R5 sobre a necessidade de pedir Ultrassom de vias urinárias em casos de hidronefrose no RN. R5 diz não saber, conversam um pouco, mas não chega a nenhuma conclusão. Colega residente liga para a preceptora para perguntar, ao desligar o telefone, R5 pergunta o que foi conversado. Residente diz que a resposta não acrescentou em nada. Outra preceptora chega no local e residente pergunta novamente, preceptora responde e colega residente e R5 escutam a resposta com atenção e concordam ao final.

				Colega residente pergunta para R5 se tais condições podem ser considerada hemorragia, R5 reponde que sim. Volta a evoluir.
				Pai de RN chama R5 e pergunta se a paciente pode tomar banho. R5 avalia a mãe e a libera para tomar o banho. Pega a RN e diz ao pai que vai colocá-la na incubadora para aquecê-la. Ao colocar na aquecedora, colega residente fala com R5 que RN tem face sindrômica, R5 concorda e ficam um tempo comparando as características da face sindrômica com a face da RN. Chamam enfermeira e pergunta, enfermeira mostra algumas características que descarta a possibilidade.
				R5 fala com enfermeira que hemoglobina está de 8.1, pergunta o que ela acha, enfermeira fala para discutir com médico. Discute caso com médico sobre o início do noripurum, médico escuta e já vai prescrevendo a medicação. Imprime e entrega para R5. R5 deixa prescrição no prontuário e avisa para colega residente e preceptora.
				Colega residente pede para R5 fazer teste do olhinho, pois está achando que está alterado. R5 faz o teste e diz que não há alteração.
				Afere temperatura do RN e faz teste do olhinho, diz ao pai que temperatura recuperou e o teste está normal. Retira RN da aquecedora e entrega para o pai.
				Realiza evolução no sistema eletrônico.
				Paciente com história de hemorragia no parto, vai no médico para dizer que hemoglobina está baixa e que paciente está com fraqueza. Médico solicita administrar noripurum e infundir 500 ml de SF. Retorna no posto de enfermagem e explica a prescrição para a TE.
				Avalia paciente que está terminando de receber solução de noripurum. Ao conversar com paciente, diz que já observou melhora.
				R5 discute o primeiro caso do paciente que avaliou com a preceptora, que escuta e carimba suas evoluções.
				R5 discute o segundo caso que avaliou com a preceptora, que escuta e carimba as evoluções.
				R5 pergunta para preceptora as pacientes que vão de alta amanhã para adiantar o sumário de alta. Chama R1 para ensiná-la como faz sumário de alta. Sentam-se no computador e iniciam. R1 vai lendo o prontuário e R5 vai escrevendo no sumário de alta. À medida que escreve vai explicando para R1 o que é relevante ter no sumário. Vai explicando para R1 as maldades que ela precisa ter ao liberar uma alta. Explica o fluxo, número de vias de cada impresso. Ensina R1 a fazer sumário de alta da mãe e do RN. Após fazerem o sumário de alta do binômio mãe e filho, R5 deixa a R1 fazer o próximo sumário.
				Colega residente traz um caso para discutir, R5 ouve e diz que é importante discutir o caso com o médico.
				Enfermeira do plantão noturno chega e preceptora e R5 inicia a transferência de cuidados.
18/07/2023	07:00	13:00	Alojamento Conjunto	Pega plantão junto com a preceptora e enfermeira. Fica escutando e mexendo no celular.
				Preceptora diz a R5 que vão receber uma paciente, realiza a transferência de cuidados. R5 fica escutando e usando o celular.
				TE pergunta para R5 sobre um caso de uma indução ao parto que participou e que achou estranha a conduta da médica. R5 escuta o caso e ao final diz que na situação da paciente não era indicado fazer a indução.
				Preceptora diz para R5 ir para o alojamento conjunto pois na sala de pré-parto está com pouco paciente. Chega no alojamento conjunto e enfermeira pede a R5 para avaliar dois pacientes. Realiza a transferência de cuidados para a R5.
				1° avaliação: entrevista mãe e orienta sobre sutura, retirada dos pontos e cuidados de higiene. Faz exame físico e vai orientando sobre a amamentação, retração do útero, edema corporal e sangramento vaginal.

				2° avaliação: avalia RN, faz exame físico e faz perguntas para a mãe se urinou, evacuou e amamentou. Verifica FC, FR, temperatura e ausculta. Explica sobre a fase oral do RN. Avalia sucção, articulações e cabeça. Troca curativo do coto umbilical e fralda, devido a presença de urina. Orienta a mãe e avó sobre os cuidados com o coto, uso de pomadas e lenços umedecidos. R5 coloca RN no peito para avaliar a qualidade da pega. Explica a frequência do banho, fala sobre registro no cartório e alta programada para amanhã. Ao final, anota dados no passômetro.
				3° avaliação: entrevista mãe. Pergunta sobre dor, sono, amamentação, etc. Faz o exame físico na mãe. Orienta sobre o uso adequado de sutiã, ingurgitamento das mamas, massagem nas mamas e a correta forma de amamentar. Explica sobre o sangramento e risco de hemorragia.
				4° avaliação: orienta sobre a face equimótica, se já urinou, evacuou e mamou. Faz exame físico completo do RN. Avalia FR, FC, temperatura, ausculta, articulações, sucção e região cefálica. Troca curativo do coto e fralda, devido a presença de diurese. Ao avaliar os MMII identifica umas vesículas no RN e chama preceptora para avaliar. Preceptora avalia, explica para a R5 algumas situações que pode gerar as lesões. Preceptora diz que vai pedir para pediatra avaliar. R5 coloca RN no peito para avaliar a qualidade da pega. Ao final, anota dados no passômetro.
				Chegam na sala de evolução e preceptora e R5 conversam sobre a lesão do RN, discutem algumas possibilidades, falam dos aspectos das lesões com detalhe, dizem nunca terem visto e preceptora liga para pediatra solicitando avaliação. R5 diz que gostaria de estar presente na avaliação médica.
				Assenta no computador para fazer as evoluções. Utiliza prontuário, caderneta da gestante e passômetro com suas anotações. Lê exames, evoluções de outros profissionais e vai evoluindo. À medida que evolui, vai preenchendo algumas informações na caderneta do RN. Faz análise de exames. Sai para tomar café e retorna para suas evoluções.
				Preceptora chama R5 para acompanhar a consulta da pediatra. R5 fica observando a conversa da pediatra com a família e ao final diz ser uma vesícula. Pediatra sai e R5 comenta com preceptora sobre a avaliação e diz não ser nada.
				R5 retorna para o computador e evolui. R5 fica um bom tempo evoluindo e vendo resultados de exames. Imprime evoluções e organiza o prontuário.
				Vai na mãe para dizer para não ficar preocupada com as lesões. Diz que vai sumir. Orienta em relação a face equimótica e diz que provavelmente vai receber alta no final do dia. Aproveita e pede a mãe o resultado do último exame de urina. Vai para o computador, evolui e faz sumário de alta. Ao final devolve exames para a mãe.
				Passa dados vitais dos pacientes para a TE. Orienta TE sobre a frequência de aferir os dados vitais.
				Discute os dois binômios com a preceptora.
				5° avaliação: exame físico da mãe e orientações.
				6° avaliação: exame físico do RN. Troca curativo do coto e fralda, presença de diurese e fezes. Avalia qualidade da pega durante a amamentação e faz orientações.
24/07/2023	07:00	13:00	Pronto Socorro	Transferência de cuidados
				1° Consulta - entrevista e avalia gestante, avalia os exames. Marido fica sentado acompanhando a consulta. Orienta em relação à qualidade e frequência das contrações para o parto.

				2° Consulta - Preceptora faz toda a entrevista da paciente. Enquanto isso, R5 vai evoluindo no sistema eletrônico. Em alguns momentos faz perguntas adicionais para a paciente. Preceptora pede a TE para coletar exame de sangue e começa a preencher impressos de internação. Preceptora orienta sobre TCLE e medidas para a segurança do paciente. Enquanto a preceptora faz o exame físico, R5 vai anotando os dados na evolução. Ao fazer a prescrição dos exames coletados, R5 apenas confirma com a preceptora os exames coletados, preceptora explica o motivo de ter solicitado o hemograma. Preceptora faz a transferência de cuidados e R5 vai anotando. Ao final, preceptora lê a evolução da R5, preceptora pede para fazer a escala de Morse e Braden.
				3° Consulta - preceptora conduz toda a entrevista e exame físico e R5 observa. Enquanto preceptora entrevista e evolui, R5 preenche impressos de internação. Enquanto preceptora explica para a paciente como funciona a indução, R5 está focada em preencher os impressos. Preceptora começa a perguntar sobre as medicações, paciente responde errado, R5 começa a ler as medicações que a paciente utiliza. R5 solicita paciente assinar o TCLE de indução ao parto. R5 faz o exame físico na paciente, mede altura uterina, ausculta FR do feto e realiza o toque. Durante o toque descreve o aspecto do colo para a preceptora evoluir. Explica para a paciente o tipo de colo que a paciente tem e o tipo de indução que vai receber. R5 fica observando a preceptora evoluir no sistema. R5 vai na sala da médica e passa o caso, a mesma assina e autoriza a indução.
				Avalia as fichas, preceptora chega e diz que paciente com dor torácica é melhor a médica atender. Pegam a próxima ficha e chamam.
				4° Consulta - preceptora inicia a entrevista e pede exame. R5 e preceptora ficam lendo o Ultrassom, R5 faz perguntas para a preceptora sobre algumas dúvidas do Ultrassom, preceptora aproveita e explica para R5 e paciente. Paciente vai ao banheiro para trocar de roupa e R5 organiza os materiais. R5 faz exame físico na paciente, vai orientando paciente sobre o hematoma detectado no Ultrassom. Passa o espéculo na paciente e avalia o sangramento. Descreve para a preceptora evoluir. Faz o toque na paciente. Descreve para a preceptora evoluir. Ao final, preceptora explica as possíveis causas do sangramento. R5 fica escutando e complementa em alguns momentos.
				Paciente que teve o parto no posto de saúde chega ao pronto atendimento através do SAMU. Preceptora inicia o atendimento na urgência, R5 fica um pouco perdida em relação a conduta. Levam para a sala de parto. Enquanto equipe organiza paciente e RN na sala, R5 lê o cartão da gestante e faz perguntas rápidas para a paciente enquanto a avalia. Ao final, senta-se no computador para internar a paciente, a todo momento, R5 vai a sala da paciente para perguntar algo sobre a gestação. Pega os exames prévios e o cartão da gestante e leva para o computador para dar continuidade a admissão. Ao final orienta o pai e vai na médica passar o caso e assinar a internação.
				5° Consulta - paciente chega com muita dor, 6 a 7 cm de dilatação. Preceptora conduz a consulta e exame físico, R5 observa preceptora e preenche impressos de internação. Paciente deambulando pelo consultório para aliviar a dor. R5 pede vaga em dois setores, faz a transferência de cuidados. TE faz o teste para COVID-19, R5 pergunta para TE como funciona o teste rápido, TE explica. Preceptora termina de conduzir o caso e R5 vai para a sala de classificação de risco.
				6° Consulta de classificação de risco - entrevista paciente, avalia Ultrassom e afere dados vitais. À medida que entrevista paciente, vai anotando no sistema eletrônico. Ao final, paciente sai do consultório e R5 comenta "posto de saúde é muito difícil, não orienta o paciente!". Sai para discutir o caso com a médica, médica explica que não é demanda de pronto socorro. R5 então, chama paciente novamente, explica o fluxo de atendimento ao paciente, explica o que é endometriose... R5 diz que pode tentar marcar a troca do DIU de cobre para o mirena. Paciente concorda. R5 faz os registros e paciente fica aguardando.

Fonte: Elaborado pelas autoras.
