# Supplementary material for: Reflective practice of nurse residents in the teaching-learning process in teaching hospitals
Source: Rev Bras Enferm. 2024 Sep 6;77(4):e20230540. doi: 10.1590/0034-7167-2023-0540 (PMC11382670; doi:10.1590/0034-7167-2023-0540)
Supplement: Translation

# Translation: A prática reflexiva de enfermeiros residentes no processo ensino-aprendizagem em hospitais de ensino

**DOI:** 10.1590/0034-7167-2023-0540pt

**Authors:** Ana Carolina de Oliveira Paiva, Kênia Lara Silva

**Affiliations:** I Universidade Federal de Minas Gerais. Belo Horizonte, Minas Gerais, Brasil

**Keywords:** Aprendizagem, Enfermeiras e Enfermeiros, Hospitais de Ensino, Prática Profissional, Preceptoria

## Abstract

**Objetivo:**

Analisar a prática reflexiva no processo ensino-aprendizagem de enfermeiros em programas de residência em hospitais de ensino de Minas Gerais, Brasil.

**Métodos:**

Estudo de caso, baseado no referencial da prática reflexiva, desenvolvido em dois hospitais de ensino. Realizaram-se observação e entrevista de residentes do primeiro e segundo ano, e incluídos, para análise em profundidade, cinco participantes, cujos dados foram submetidos à análise de distribuição de frequência e à Análise do Discurso Crítica.

**Resultados:**

Em 519 atividades observadas, houve identificação de elementos de reflexão em 22,2%, especialmente escuta com atenção operativa e expressão de dúvidas. Os discursos indicaram a prática como o melhor momento para o ensino-aprendizagem pelo potencial de produzir reflexões. Aprender fazendo e discussão de casos foram consideradas estratégias potenciais para a aprendizagem reflexiva.

**Considerações Finais:**

Evidenciou-se a reflexão conhecer-na-ação como a predominante na formação dos residentes, com poucas oportunidades para reflexão sobre a reflexão-na-ação.

## INTRODUÇÃO

A Residência em Area Profissional da Saúde, instituída pela Lei n° 11.129 de 2005, é caracterizada como educação em serviço e destinada aos profissionais de saúde, com exceção da categoria médica^(1)^. Desempenha um papel primordial na formação de especialistas, alinhada aos princípios e diretrizes do Sistema Único de Saúde (SUS) e propõe a associação entre teoria e prática, de forma a promover nos residentes a reflexão crítica sobre a realidade experienciada^(2, 3)^.

A implantação dos Programas de Residência em Área Profissional da Saúde (PRAPS) no Brasil data de mais de 10 anos. Contudo, estudos apontam desafios relacionados ao processo de formação dos residentes que ainda precisam ser superados. Dentre eles, destacam-se o modelo de ensino tradicional, a centralidade no saber médico, a ausência de interação entre os profissionais de saúde, as práticas fragmentadas e desconectadas do contexto social do paciente e a desqualificação dos profissionais para a preceptoria e o ensino crítico e reflexivo sobre as vivências da prática profissional^(2, 4, 5)^.

Nesse sentido, as evidências sugerem a formação do residente voltada para o modelo biologicista, cuja visão do paciente é reducionista e fragmentada, além da maximização das habilidades técnicas em detrimento das relacionais, o que posiciona o aluno em um papel pouco ativo^(2, 4, 5)^. Com isso, infere-se que a prática reflexiva no processo ensino-aprendizagem dos PRAPS se encontra fragilizada, com foco nos procedimentos, na repetição de técnicas e na transmissão de conhecimento, além da escassa discussão teórica e da pouca interação entre preceptor-tutor e ensino-serviço. Associa-se, ainda, a presença de preceptores sem formação pedagógica que acumulam muitas funções e, às vezes, não se identificam com o papel de formadores em saúde. Isso gera um ciclo de transferência de tarefas para os residentes, o que repercute em sua aprendizagem.

Nesse contexto, questiona-se: como ocorre a prática reflexiva no processo ensino-aprendizagem de enfermeiros em programas de residência? Quais elementos da prática reflexiva são revelados nesse processo? Investigar a prática reflexiva no ensino-aprendizagem de enfermeiros em PRAPS se justifica pela relevância desse processo formativo, na formação de profissionais críticos e criativos que refletem em e sobre as ações desenvolvidas. Há reconhecimento do papel das residências como estratégia de defesa e formação para o SUS. Assim, os resultados do estudo podem fortalecer esse modelo de ensino, além de contribuir com as reformulações dos projetos políticos pedagógicos dos PRAPS.

A presente pesquisa está ancorada no referencial teórico de Donald Schon sobre a prática reflexiva no processo de ensino-aprendizagem^(6)^. De acordo com o pedagogo, a formação profissional ainda é direcionada para a excelência técnica e científica, voltada para o modelo cartesiano, no qual a teoria se encontra desvinculada da prática, sendo esta última vista como consequência da formação teórica. Esse processo não favorece o desenvolvimento de profissionais criativos capazes de atender às demandas do mundo do trabalho^(6)^.

A partir dessa perspectiva, propõe-se uma associação entre teoria e prática para a formação profissional em um ensino reflexivo, cuja capacidade de reflexão seja incentivada por meio da interação entre professor e aluno em diferentes situações práticas, ou seja, a aprendizagem por meio do fazer. O referencial sobre a prática reflexiva postula que uma atuação profissional eficaz é alcançada somente por meio da reflexão sobre as situações incertas e conflituosas da prática diária^(6)^.

Destaca-se que a literatura sobre a prática reflexiva, proposta por Donald Schön, no processo ensino-aprendizagem dos programas de residência em enfermagem, ainda, é escassa nacionalmente^(2, 3, 7, 8, 9, 10, 11)^. Os estudos realizados estão voltados para a formação de preceptores, a percepção dos residentes e preceptores sobre o processo ensino-aprendizagem nos PRAPS, além da educação interprofissional e da educação permanente nos PRAPS. Assim, persiste a necessidade de analisar o processo ensino-aprendizagem nos PRAPS, orientado por um referencial teórico que ofereça sustentação aos princípios inerentes à residência, qual seja, a vivência prática reflexiva.

## OBJETIVO

Analisar a prática reflexiva no processo ensino-aprendizagem de enfermeiros em programas de residência em hospitais de ensino de Minas Gerais, Brasil.

## MÉTODOS

### Aspectos éticos

Pesquisa aprovada, em 2021, pelo Comitê de Ética em Pesquisa da Universidade Federal de Minas Gerais, atendendo aos princípios éticos da Resolução 466/2012. Os participantes assinaram o Termo de Consentimento Livre e Esclarecido e foram codificados para garantir sigilo e anonimato.

### Tipo de estudo

Trata-se de uma pesquisa qualitativa, do tipo estudo de caso único. Definiu-se como caso a prática reflexiva no processo ensino-aprendizagem de enfermeiros em programas de residência. Adotaram-se os critérios propostos pelo *Consolidated Criteria for Reporting Qualitative Research* (COREQ)^(12)^.

### Procedimentos metodológicos

Este estudo faz parte de uma tese de doutorado, realizada em três fases: análise documental, observação participante periférica e entrevista semiestruturada^(13)^, formando uma triangulação de dados para o estudo de caso. Neste artigo, apresentam-se dados da observação e entrevista.

### Cenário do estudo

O contexto do caso no qual se desenvolve a prática reflexiva no processo ensino-aprendizagem foi o PRAPS uniprofissional e multiprofissional. As unidades de análise foram dois hospitais de ensino localizados em Minas Gerais, Brasil. Um dos hospitais, denominado unidade de análise 1, é um hospital geral com vocação para atendimento em trauma, contando com PRAPS multiprofissional em saúde do idoso e PRAPS uniprofissional em Intensivismo, Urgência e Trauma; o outro, denominado unidade de análise 2, é especializado em saúde da mulher com PRAPS uniprofissional em enfermagem obstétrica.

### Fonte de dados

A população do estudo foi composta por 47 residentes de enfermagem, dos 53 que estavam matriculados nos PRAPS nos hospitais cenários, após aplicação dos critérios de exclusão. Três residentes do PRAPS enfermagem obstétrica estavam de licença-saúde no período da coleta de dados; um residente do primeiro ano do PRAPS saúde do idoso contribuiu com o teste piloto; e dois residentes do segundo ano do PRAPS saúde do idoso desistiram da especialização à época da coleta.

Para a seleção dos participantes, utilizou-se o método não probabilístico do tipo amostragem aleatória estratificada proporcional, com a finalidade de fortalecer a representatividade da amostra^(13)^. A população foi dividida em grupos homogêneos: um estrato referente aos residentes do primeiro ano de residência e outro, do segundo ano, considerando que a prática reflexiva é progressiva durante a formação profissional. A interrupção da coleta foi determinada pela saturação temática dos dados, confirmada pela constatação de redundância e recorrência de informações. Este modelo de saturação observa a recorrência de códigos ou temas identificados, exemplificados nos dados^(14)^. Esse processo foi confirmado a partir da leitura e pré-análise de cada material coletado referente aos participantes, individualmente, após a inclusão de três residentes do primeiro ano de residência e dois do segundo ano.

O Quadro 1 apresenta a população do estudo e a distribuição dos residentes, de acordo com as unidades de análise.

**Quadro 1:** População do estudo e distribuição dos residentes de enfermagem observados por unidade de análise, Belo Horizonte, Minas Gerais, Brasil, 2023

Unidades de análise	PRAPS	Ano residência	Nº residentes matriculados nos PRAPS	Código residente selecionado
1	Multiprofissional Saúde do Idoso	1º ano	2	R1
		2º ano	2	-
	Uniprofissional Intensivismo, Urgência e Trauma	1º ano	2	R4
		2º ano	1	R2
2	Uniprofissional Enfermagem Obstétrica	1º ano	24	R3
		2º ano	22	R5

*Nota: PRAPS - Programas de Residência em Area Profissional da Saúde.*

### Coleta e organização dos dados

A coleta foi realizada de agosto de 2022 a julho de 2023. Antes da coleta de dados, efetuou-se um estudo piloto. Realizou-se uma observação de 2 horas, seguida por uma entrevista com duração de 10 minutos e 36 segundos com um residente do primeiro ano do PRAPS de Saúde do Idoso. Ao final da observação, confirmou-se a aplicabilidade do roteiro de observação proposto. Em relação à entrevista, identificou-se a necessidade de adaptar as perguntas para garantir a compreensão pelo residente. Após reescritas, as perguntas foram testadas em uma nova entrevista piloto, na qual se confirmou novamente a aplicabilidade. Os dados do estudo piloto não foram incluídos na pesquisa final.

Na observação, o foco foi a prática reflexiva a partir do residente. Observaram-se os residentes individualmente com a finalidade de detectar cenas de ensino-aprendizagem. Ao percebê-las, registravam-se notas em um diário digital do pesquisador, que incluíam: (1) descrição da atividade observada; (2) elementos da prática reflexiva expressos pelo residente; (3) atores que potencializavam essa reflexão; e (4) momentos em que essas cenas de ensino-aprendizagem aconteciam, seja durante uma atividade prática, teoria, estudo de caso ou tutoria.

É importante enfatizar que os alunos demonstram reflexão por meio das suas performances (6). Assim, ao observar os residentes, a pesquisadora identificou algumas pistas das performances reflexivas em situações em que o aluno: (1) se surpreendia com algo; (2) fazia pausa durante a execução de uma atividade, acompanhada por silêncio; (3) comentava ou criticava algo sobre a atividade; (4) escutava com atenção operativa; e (5) expressava dúvidas. Essas pistas foram denominadas na pesquisa como elementos da prática reflexiva.

Essa etapa resultou na observação de cinco residentes por um total de 156 horas e 10 minutos. As observações ocorreram durante dias de semana e finais de semana, nos turnos matutino e vespertino. O tempo médio por dia de observação foi de 6 horas, sendo cada residente observado por aproximadamente 31 horas.

Após finalizar todas as observações de cada residente, prosseguia-se para a entrevista desse aluno. A entrevista foi guiada por um roteiro semiestruturado que continha breves relatos das cenas de ensino-aprendizagem observadas pela pesquisadora durante a coleta. A partir desses relatos, o residente era estimulado a refletir sobre suas percepções da cena, bem como sobre fatores que favoreceram ou dificultaram a reflexividade naquela situação e em outros momentos de ensino-aprendizagem. As entrevistas com os cinco residentes observados foram gravadas em áudio, com duração média de 19 minutos e 25 segundos, totalizando 1 hora, 37 minutos e 5 segundos.

### Análise dos dados

A fim de garantir o anonimato, cada residente recebeu um código (R1, R2, R3, R4 e R5), sendo o mesmo atribuído ao residente tanto na observação quanto na entrevista. Os dados da observação foram organizados em uma planilha Excel e, a cada observação, descreviam-se as atividades desenvolvidas pelo residente e observadas pela pesquisadora. Em seguida, nomeava-se a atividade descrita e a analisava-se quanto à identificação de elementos da prática reflexiva expressos pelo residente, aos atores que potencializavam essa reflexão e ao momento do processo de ensino-aprendizagem em que a atividade se desenvolvia. Ao finalizar a descrição e análise das atividades executadas pelos cinco residentes, durante as 156 horas e 10 minutos de observação, a pesquisadora prosseguiu para a fase de compilação dos dados e análise, por meio da distribuição de frequência absoluta e relativa.

As entrevistas foram transcritas utilizando o *software* InqScribe, em sua versão gratuita. A fim de sinalizar no texto as justaposições entre falantes, pausas, silêncios, entonação, interrupções e trechos incompreensíveis, adotaram-se convenções de transcrição de Marcuschi^(15)^. Essa convenção expressa não somente a fala, mas também elementos não verbais, capazes de demonstrar reflexão, como pausas, silêncios, gestos típicos de surpresa e atenção.

Realizou-se a Análise do Discurso Crítica, na perspectiva de Fairclough^(16)^, com o intuito de analisar os dados empíricos a partir do conteúdo manifesto nos discursos, correlacionando-os ao referencial teórico. De modo operacional, elaborou-se uma matriz analítica para extração dos dados de cada entrevista, que foi minuciosamente examinada, identificando-se, linha a linha, parágrafo a parágrafo, os elementos discursivos. Após essa etapa, realizaram-se a leitura vertical de cada eixo e a leitura transversal do material, procurando-se compreender, de forma aprofundada, o corpus de análise que permitisse a reconstrução dos conhecimentos sobre o fenômeno em questão.

## RESULTADOS

Os residentes participantes do estudo tinham um tempo médio de formação na graduação de 2,6 anos e estavam todos no primeiro curso de especialização. Apenas dois deles apresentavam experiência profissional prévia antes de ingressar no PRAPS.

Durante 156 horas e 10 minutos de observação, foram analisadas 519 atividades desenvolvidas pelos residentes ao longo dos respectivos PRAPS. Essas atividades foram categorizadas em quatro momentos de ensino-aprendizagem: atividade prática, teórica, estudo de caso e tutoria (Tabela 1).

**Tabela 1:** Número de atividades com elementos de reflexão, elementos da prática reflexiva, identificados e atores, de acordo com os momentos de ensino-aprendizagem, Belo Horizonte, Minas Gerais, Brasil, 2023

Momentos ensino-aprendizagem	Tempo de observação	Quantitativo de atividades observadas	Quantitativo de atividades com elementos da prática reflexiva (n,%)	Quantitativo de Elementos da prática reflexiva	Quantitativo de atores que potencializaram a prática reflexiva
Atividade Prática	137h10m	500	101 (20,2)	163	143
Atividade Teórica	4h	4	3 (75)	4	4
Estudo de Caso	6h	8	7 (87,5)	9	9
Tutoria	9h	7	4 (57,1)	7	7
Total	156h10m	519	115 (22,2)	183	163

As 519 atividades foram categorizadas em 30 tipos, conforme a Tabela 2, que destaca o número de atividades em que houve a identificação de pelo menos um elemento da prática reflexiva. Além disso, a Tabela 2 apresenta as 13 categorias de elementos da prática reflexiva verificadas nas atividades desenvolvidas pelos residentes: a. escuta com atenção operativa (43); b. expressa dúvidas (34); c. diz ou critica algo sobre a atividade (19); d. associação (18); e. troca de ideias/discussão (18); f. pausa durante a execução da atividade, acompanhada pelo silêncio (9); g. detecta a necessidade de mudar ou fazer algo diferente durante ou após a ação (8); h. explica a própria atividade (7); i. insight (7); j. investigação (6); k. avalia o que realizou (5); l. surpreende com algo (5); m. comparação (4).

**Tabela 2:** Número de atividades com identificação de elementos da prática reflexiva e categorias de elementos da prática reflexiva, Belo Horizonte, Minas Gerais, Brasil, 2023

Atividades (n)	Atividades com elementos da prática reflexiva (n,%)	Quantitativo de elementos da prática reflexiva	Elementos da prática reflexiva identificados nas atividades em ordem decrescente
Avaliação paciente (91)	14 (15,4)	23	b, d, a, f, j, e, k, c, l
Prescrição ou registro de enfermagem (73)	7 (9,6)	11	b, j, e, m, g, c, a
Assistência ao paciente (57)	10 (17,5)	17	m, h, b, f, i, k, a, e
Orientação (52)	-	-	-
Transferência de cuidados (36)	3 (8,3)	3	b, a
Discussão caso clínico (33)	14 (42,4)	18	a, c, b, e, g, i, j, l
Gestão dos processos de trabalho (31)	2 (6,5)	2	c, b
Execução de técnica ou procedimento (23)	8 (34,8)	12	g, a, h, f, c, j
Gestão equipe de enfermagem (23)	3 (13)	5	a, d, k, g
Observa profissional executar atividade (21)	14 (66,7)	25	a, b, e, d, g, c, h, i, l
Análise de exames (19)	8 (42,1)	14	b, c, e, d, a, l
Gestão de leitos (11)	4 (36,4)	7	b, i, c, a, f,
Esclarece dúvidas (9)	6 (66,7)	13	b, a, e, k, c
Limpeza e desinfecção (6)	-	-	-
Recebe orientação (5)	2 (40)	2	a, l
Apresentação estudo de caso (3)	2 (66,7)	3	d, c
Discussão do estudo de caso (3)	3 (100)	4	d, a, h
Elaboração TCR (3)	3 (100)	7	c, d, k, g, a, b
Feedback (3)	1 (33,3)	1	i
Assiste apresentação de trabalho (2)	2 (100)	3	e, d, c
Assiste apresentação estudo de caso (2)	2 (100)	2	a
Aula expositiva (2)	1 (50)	2	d, c
Dinâmica (2)	1 (50)	1	d
Elaboração estudo de caso (2)	2 (100)	3	e, d
Expressa vivências na residência (2)	-	-	-
Roda de Conversa (2)	2 (100)	4	c, d, a
Apresentação de trabalho (1)	1 (100)	1	d
Reunião Colegiado (1)	-	-	-
Reunião PRAPS (1)	-	-	-

*Notas: TCR - Trabalho de Conclusão de Residência; PRAPS - Programas de Residência em Área Profissional da Saúde.*

Por meio da Tabela 2, evidenciam-se sete atividades nas quais os residentes, em 100% das cenas, expressaram pelo menos um elemento de reflexão. Das sete identificadas, duas foram categorizadas como momentos de ensino-aprendizagem de estudo de caso (assistem a apresentações e discutem estudos de caso); duas como atividades teóricas (apresentam e assistem a apresentações de trabalhos); e uma como tutoria (roda de conversa). As duas atividades restantes foram categorizadas como atividades práticas, uma vez que ocorreram dentro da carga horária destinada para isso. No entanto, destaca-se que a elaboração de Trabalho de Conclusão de Residência (TCR) e de estudo de caso são atividades que estão associadas aos momentos de tutoria e estudo de caso, respectivamente. Logo, nenhuma das sete atividades identificadas está relacionada diretamente à atividade prática.

A escuta com atenção operativa foi o elemento de reflexão mais expresso pelos residentes, seguido pelos elementos “expressa dúvidas” e “diz ou critica algo sobre a atividade”. Já os elementos “avalia o que realizou”, “surpreende com algo” e “comparação” foram os menos expressos pelos residentes.

Os próprios residentes, em autorreflexão, foram os que mais potencializaram a reflexão (42,9%), seguidos pelos preceptores (25,8%) e colegas residentes de enfermagem (9,2%). Já os tutores (1,2%), pacientes (1,2%) e os professores (0,6%) foram os atores que menos potencializaram a reflexão dos residentes.

A atividade com maior frequência de observação foi a de avaliação do paciente, com 91 cenas observadas, das quais, em 14 (15,4%), houve identificação de nove tipos de elementos da prática reflexiva e de seis atores estimulando a reflexão. Destaca-se o elemento “expressa dúvidas”, que se repetiu em sete ocasiões, e os residentes em “autorreflexão”, em nove situações.

A atividade de prescrição ou registros de enfermagem foi a segunda mais observada, com 73 cenas, das quais, em sete (9,6%), houve detecção de sete tipos de elementos de reflexão, com três atores potencializando a reflexão. A relação da maior frequência de execução da atividade com o baixo número de identificação de elementos de reflexão é consonante ao discurso de R1. Em entrevista, o participante caracterizou esse processo como “cansativo” e “repetitivo”. Por meio do operador argumentativo “mas”, R1 estabeleceu uma relação adversativa sobre a atividade de registro de enfermagem, afirmando que, na trajetória como residente, inicialmente gostava de evoluir os pacientes, mas que, atualmente, considera a atividade repetitiva:

[...] *evoluir é uma coisa que eu gostava muito de fazer antes. Mas hoje tá, hoje tá se tornando cansativo pra mim, sabe? Evoluir. Porque é muito repetitivo. Aquele negócio repetido.* (R1)

Esse discurso difere da fala de R2 que sinalizou o registro de enfermagem como a atividade que mais estimula a reflexão. O residente percebeu a atividade como oportunidade para ler as evoluções da equipe multidisciplinar e compreender a história clínica do paciente.

*Eu acho que é quando EU tô no, no computador pra EVOLUIR, e aí, eu sempre busco ver qual é a abordagem de outros profissionais frente àquele paciente também. Então, eu tenho o meu cuidado. E aí, eu tento ver, é,* (+) *entender mesmo, assim, todo o contexto do paciente, né? Então, eu acho que, assim, quando eu tô mesmo* (+)*. Eu vou se... Eu avalio o meu paciente e vou sentar pra evoluir, eu tento entender toda a história dele, né? Então eu acho que* (é) *mais quando eu tô na frente do computador.* (R2)

Além disso, os residentes mencionaram como estratégia de ensino-aprendizagem a prática do aprender fazendo:

[...] *a técnica de ensino-aprendizagem é assim: “hum, nunca fiz isso. Vou fazer”. Aí, eu ponho a mão na massa e faço, sabe?* (R1)

*E, aí, eu vejo muito que é isso. Às vezes, a gente tá ali na assistência, a gente aprende muito a esse, esse fazer. A resolver aquele problema, né? E nem sempre entender o que que tá por trás. Mas a gente aprende, sim.* (R2)

No primeiro excerto, a estratégia de ensino-aprendizagem “aprender fazendo” foi considerada habitual. O operador argumentativo “mas”, utilizado por R2 no último excerto, ilustra que, mesmo sem compreender completamente “esse fazer”, ele conseguia aprender. Ademais, R2 destacou a discussão de casos clínicos como uma estratégia potencial para o ensino-aprendizado. Esta afirmativa é sustentada pelos elementos discursivos “estratégia (que) talvez fosse melhor, que agregaria mais, fixaria melhor o conhecimento” Ratifica-se, assim, a necessidade de envolvimento do preceptor nas discussões, com o intuito de exercer uma posição mais ativa no processo.

*E aí, eu acho que* (+) *uma estratégia* [que] *talvez fosse melhor, que agregaria mais, fixaria melhor o conhecimento, seria as discussões em, é, dentro do setor mesmo, sabe? É, envolvendo, é, discussão mesmo DA situação da clínica do paciente, é, tanto do ponto de vista DO residente, quanto de vista, o ponto de vista do preceptor, mesmo.* (R2)

R1, ainda, ressaltou a importância das discussões de casos para além dos momentos de dúvida dos residentes, frisou a necessidade de “aprofundar” e “entender melhor o caso dele (paciente)”.

*Às vezes, eu sinto falta mais de a gente sentar e preceptor e, e residente, e discutir mais os casos, sabe? Não apenas, não apenas de, de, de no momento que precise* [...]. [...] *acaba que tem paciente que a gente não consegue aprofundar, tipo, entender melhor o caso dele, sabe?* [...] *eu sinto falta de, dessa discussão, mais assim, de trabalhar mais essa questão dos diagnósticos de enfermagem, das intervenções, não ficar meio, só aquela, a situação de repetição, sabe?* (R1)

Na entrevista de R3 e R4, é perceptível a expressão de alguns elementos de reflexão, como a expressão de dúvidas.

*Eu sempre vou em busca de um preceptor pra tirar minhas dúvidas, sempre.* (R3)

*Geralmente, eu me sinto bem confortável, é, por exemplo, de, de se eu tiver alguma dúvida, de perguntar.* (R4)

Nesse contexto, o léxico “dúvida” foi citado por 11 vezes no discurso de R2, correlacionado ao fazer e em operação ideológica de racionalização para justificar o conjunto de relações, isto é, a partir da dúvida, a R2 não faz e, então, procura a preceptora.

*Eu lembro exatamente que a minha dúvida, na verdade, era se eu poderia tentar lavar ou se eu tinha que aspirar. Então eu preferi não fazer. Mas é isso. Acho que na... Geralmente, a gente se vê em situações, assim, de dúvida mesmo... E, aí, a gente precisa do auxílio do preceptor.* (R2)

Em relação aos atores, os próprios residentes em autorreflexão (38%) foram os que mais potencializaram a prática reflexiva durante o momento de atividade prática. Em seguida, vêm os preceptores (25,8%) e outros enfermeiros (8%) presentes no cotidiano dos residentes. Nos excertos abaixo, identificam-se dois atores: o próprio residente e o preceptor. O próprio residente em autorreflexão expressa: “Estou com dificuldade” e “Eu achei que ia ser supertranquilo passar a sonda. Cheguei lá, e a mulher ainda estava numa maca, isso, aí eu pedi ajuda da Preceptora Y”, momentos em que R1 analisa a situação e reflete sobre as próprias dificuldades.

*Aí, eu tenho dificuldade, eu chamo Preceptora Y, “ô Preceptora Y, tô com dificuldade”. Aí ela vai me norteando, sabe?* [...] *quando eu fui passar a sonda nela* [paciente], *eu jurava que ela* [paciente] *era muito mais jovem que eu, inclusive, então eu achei que ia ser supertranquilo passar a* [sonda]. *Cheguei lá, a mulher ainda tava numa maca, uma maca, isso, aí eu pedi ajuda da Preceptora Y, aí Preceptora Y veio na... ela foi pra paciente, parece. Ela gosta de ensinar.* [...] *É, executando a técnica, que eu aprendi.* (R1)

O papel da preceptora em estimular a reflexão é evidenciado quando o residente explica: “Aí ela (preceptora) vai me norteando” e “aí a Preceptora Y veio na... ela foi para a paciente, parece. Ela gosta de ensinar... É, executando a técnica, que eu aprendi”

No discurso de R1, a dúvida e a tentativa de fazer são apontadas como elementos disparadores e mobilizadores da aprendizagem. Por meio da dúvida, o participante apresenta a oportunidade de aprender por iniciativa própria, e não por direcionamento do preceptor. Entretanto, mesmo o preceptor sendo um dos atores mais presentes, evidencia-se, pelo discurso de R2, a posição passiva do preceptor em alguns momentos do processo de ensino-aprendizagem:

*Mas é, mas a maioria é, das situações de ensino-aprendizado, eu vejo que sou eu indo atrás do preceptor, né?* (R2)

A observação dos residentes no momento de ensino-aprendizagem de atividades teóricas foi realizada durante quatro horas, nas quais foram identificados três elementos de reflexão (associação; diz ou critica algo sobre a atividade; e troca de ideias e discussão), com dois atores (autorreflexão e colega residente de enfermagem) potencializando essa reflexão.

No momento de estudo de caso, a observação dos residentes foi realizada durante seis horas, nas quais foram identificados quatro elementos de reflexão (associação; escuta com atenção operativa; diz ou critica algo sobre a atividade; e explicação da atividade), com três atores (colega residente de enfermagem, o próprio residente em autorreflexão e enfermeiro) potencializando essa reflexão.

Por fim, a observação dos residentes no momento de tutoria foi realizada durante nove horas. Das cinco atividades observadas, em três foram identificados três elementos de reflexão (associação; diz ou critica algo sobre a atividade; e escuta com atenção operativa), com quatro atores (o próprio participante em autorreflexão, tutor, colega residente de enfermagem e professor) potencializando essa reflexão.

Sobre esses momentos do ensino-aprendizagem, os participantes expressaram:

*A gente, n, na residência a gente tem essa FALTA, assim, dessa parte teórica de aprendizado.* [...] *Mas eu acho que, também, direcionar conteúdos de eixo específico,* [...]. *Ah, eu, eu acho que se eles fornecessem pra gente, ah, conteúdos, mesmo, de aprofundamento teórico, seria, é, interessante. Assim* (+)*, ajudaria bastante nesse processo de unir, né? Prática e teoria.* (R2)

*Eu acho que os estudos de caso, mesmo. Pegar um caso e a gente falar sobre as, as possíveis complicações daquilo... Ou possíveis desfechos positivos. Que exames a gente pode ficar mais atento, como interpretar. Acho que essas coisas que vai, vai realmente estimular, assim, o raciocínio da gente.* (R3)

*Geralmente a parte teórica a gente não tá tendo muito* [...] *Agora vai ter só a tutoria, tipo. Só de aula mesmo, mas não é, assim, não é muito voltado pra parte prática de enfermagem. A gente tá vendo mais num contexto, é, um contexto geral. Então, a gente tá vendo mais sobre gestão do cuidado, assim. Não é nada, tipo, direcionado PRA parte prática de enfermagem. Ai, como acaba que não tem muito essa parte prática, meio que tento compensar em casa, é, tipo, tentando estudar no final de semana.* (R4)

## DISCUSSÃO

Uma base educacional sólida e de boa qualidade, somada à diversidade de experiências na prática clínica, favorecem tanto a aquisição de conhecimentos e habilidades de forma mais segura e rápida quanto a progressão do profissional para a proficiência^(17)^. Desta forma, é importante salientar que atividades de ensino teóricas são imprescindíveis, mas são as atividades de ensino práticas que possibilitam a aplicação dessa teoria e o desenvolvimento de habilidades, trabalho em equipe, liderança e autoconfiança. As situações práticas expõem o aluno a vivenciar distintas experiências necessárias para a reflexão-na-ação diante de situações de incerteza, singularidade e conflito.

Os resultados da pesquisa revelaram, por meio das observações dos residentes, que as atividades em que houve identificação de 100% dos elementos de reflexão, em número absoluto, foram pouco desenvolvidas pelos residentes, uma vez que estão relacionadas às estratégias educacionais teóricas dos PRAPS. A baixa oferta desses tipos de atividades está de acordo com o estabelecido em normativas que definem 20% da carga horária total dos PRAPS para atividades teóricas e 80% sob a forma de estratégias educacionais práticas e teórico-práticas^(18)^.

Verificou-se que as atividades mais executadas pelos residentes no dia a dia do PRAPS estiveram relacionadas ao momento de ensino-aprendizagem de atividade prática. Ou seja, atividades como avaliar o paciente, registrar a assistência de enfermagem e prestar assistência ao paciente são as mais frequentes no cotidiano dos aprendizes, mas que apresentam baixo percentual de identificação de elementos de reflexão.

Ao repetir frequentemente a execução de determinada tarefa, o estudante desenvolve o agir tácito, ou seja, o conhecer-na-ação. Isso significa que o aprendiz poderá, em dado momento, descobrir que internalizou a performance habilidosa^(6)^. Assim, a atividade que começou como uma construção imitadora, agora, permite ao aluno experimentar algo próprio, um conhecimento novo do próprio repertório, disponível para uso^(6, 10)^.

Em vista disso, infere-se que, no cotidiano dos residentes observados, prevaleceu a reflexão do tipo conhecer-na-ação, ato espontâneo e automático, em que o aprendiz executa sequências fáceis de atividades, sem ter que pensar a respeito. É um processo tácito, que se coloca espontaneamente, sem deliberação consciente e que funciona proporcionando os resultados pretendidos, enquanto a situação estiver dentro dos limites do que se aprende a tratar como normal^(3, 6, 9)^. Esse resultado coaduna com um estudo canadense, que afirma que o estudante utiliza a racionalidade técnica para resolver situações lineares, em que as mesmas soluções podem funcionar em casos diferentes. No entanto, destaca que, diante da situação de incerteza, única e não totalmente conhecida, como as vivências dos enfermeiros na pandemia da Covid-19, a racionalidade técnica nem sempre é suficiente para resolver a situação. Nessas ocasiões, é necessária a prática reflexiva para alcançar tentativas de resolução^(19)^.

Nessas circunstâncias, em que o limite da normalidade é ultrapassado, os residentes deixam de agir automaticamente e se deparam com situações inesperadas, denominadas como surpresas, que podem abalar as categorias construídas do conhecimento-na-ação^(6)^. Identifica-se, assim, a ocorrência da prática reflexiva no cotidiano dos processos de ensino-aprendizagem nos PRAPS.

Diante desse contexto, evidenciam-se os residentes subindo a escada da reflexão ao desenvolverem atividades como observar um profissional executando uma tarefa, esclarecer dúvidas e discutir casos clínicos. Isto é, quando os residentes são capazes de agir diante das situações inusitadas da prática profissional, eles mobilizam, além do conhecimento tácito, o conhecimento proveniente de experimentações de outras performances^(^6-7^)^. Nessas ocasiões, ocorre a superação do conhecer-na-ação, que se refere ao nível mais elementar da reflexão, e os conhecimentos vão se revelando por meio de ações inteligentes, ou seja, performances físicas publicamente observáveis.

Nesta pesquisa, captaram-se performances habilidosas dos residentes de escutar com atenção operativa; expressar dúvidas; e trocar ideias com outros profissionais. Este achado se respalda em Schön^(6)^, que afirma que, durante as tentativas do estudante de produzir o design, o dizer e o ouvir do instrutor têm elevado potencial para a eficácia, uma vez que o estudante escuta a instrução com atenção operativa, ou seja, com prontidão especial para traduzir aquilo que ele ouve em ações.

A ocorrência da prática reflexiva e a consequente expressão dos elementos de reflexão produzem, no profissional, nova compreensão da experiência vivenciada, que, por sua vez, ocasionam a incorporação de novos aprendizados da prática e, consequentemente, estimulam o crescimento pessoal e profissional. O aprendiz que passa por esse processo cíclico se torna um profissional mais reflexivo e capaz de propor novas performances de produção de design^(19)^.

Diante do exposto, entende-se o motivo da atividade “observar o profissional desenvolvendo uma tarefa” ser uma das que mais estimularam os residentes a expressarem elementos de reflexão, sendo o mais frequente a escuta com atenção operativa. Nessas interações entre aluno e instrutor, estabelece-se o ensino prático reflexivo, em que os residentes, além de observarem as performances habilidosas do profissional observado, têm a oportunidade de estabelecer um diálogo reciprocamente reflexivo com o profissional. Essa troca recíproca entre o instrutor e o estudante tende a promover uma sequência de projetos de designs, que motivam a crescente capacidade do estudante em produzir um design competente^(6)^.

Nesses cenários, apresentam-se os próprios residentes como os que mais potencializaram as próprias reflexões, seguidos pelos preceptores. Portanto, indica-se que a autorreflexão potencializa a prática reflexiva no cotidiano da residência, nas situações em que os residentes exercem papel ativo no processo ensino-aprendizado. Os achados das entrevistas foram confirmatórios e os participantes assumiram atitudes críticas nos discursos, apontando os momentos de maior reflexão e indicando oportunidades para ampliar esse processo, no sentido de exercer posição mais ativa na aprendizagem.

No entanto, evidencia-se que os elementos de reflexão expressos pelos residentes voltaram-se para a reflexão-na-ação e reflexão sobre a ação, com poucas oportunidades para a reflexão sobre a reflexão-na-ação. Esta última refere-se ao ato do profissional refletir a posteriori sobre a reflexão-na-ação passada, o que irá consolidar o entendimento da situação e, assim, proporcionar a instituição de novas estratégias^(6, 7, 9)^. Aponta-se a relevância do enfermeiro desenvolver o pensamento crítico-reflexivo no decorrer da formação, uma vez que a realidade prática exige capacidade de tomada de decisões assertivas, baseadas em evidências científicas e análises críticas dos contextos clínicos e sociais em que está inserido. Além disso, requer deste profissional a capacidade de promover questionamentos e discussões interprofissionais para garantir a qualidade do cuidado^(11)^.

Um estudo de revisão integrativa contribui nesta análise, ao afirmar que as estratégias de ensino utilizadas pelos PRAPS precisam contribuir para que o residente seja um ator ativo do próprio processo de aprendizagem^(20)^. O aprendizado no serviço é necessário para despertar a criatividade e a independência do aluno, bem como fornecer experiências que o levem a problematizar e propor alternativas de intervenção condizentes com os cenários vivenciados^(8, 20)^.

Reitera-se que o PRAPS propõe a formação qualificada dos profissionais de saúde para o SUS, capacitados para transformar as práticas diárias e compreender as necessidades da saúde pública. Para isso, constitui-se como estratégia formativa que opera contra o modelo biologicista e a reprodução acrítica dos aprendizes ainda advindas das instituições de ensino^(21)^.

Nesse contexto, discutem-se os desafios que o enfermeiro preceptor enfrenta para inserir na rotina as atividades de supervisão e a orientação de alunos, estimulando uma postura mais ativa que promova atitudes de investigação, crítica e reflexão do estudante. Estudos apontam o acúmulo do trabalho assistencial, docente e de gestão, o qual reduz a preceptoria às atividades de repasse das rotinas de trabalho e atividades assistenciais, prejudicando o adequado processo de formação dos residentes^(2, 22, 23)^. Destaca-se a relevância da formação pedagógica do preceptor para implementar um processo ensino-aprendizagem voltado para o pensamento e ação crítico-reflexiva^(2, 20)^.

Nos discursos dos residentes, destacam-se as situações incomuns como os momentos que mais estimulam a reflexão e, consequentemente, o aprendizado, sendo o preceptor a primeira referência diante da dúvida. As situações consideradas incomuns refletem o repertório de conhecimentos, ou seja, a cada nova experiência do aprendiz, o profissional enriquece o repertório de conhecimentos e experiências^(6, 10)^. Assim, situações não familiares tornam-se familiares, porque o residente acumulou um repertório que o torna capaz de lidar com diferentes situações. Ou seja, cada experiência nova de reflexão-na-ação enriquece o repertório^(6, 22)^.

Além disso, outra circunstância que ocorre nos momentos de atividades práticas e que agrega repertório de conhecimento é a prática do aprender fazendo. O aprender fazendo corresponde ao que se denomina como experimentação ativa, isto é, a atividade pela qual o investigador em reflexão-na-ação confirma ou refuta uma ação ou hipótese, o que promove a autonomia do residente^(6, 9)^. Os entrevistados, em seus discursos, além de tratarem esta prática como um acontecimento natural, por meio de afirmações avaliativas explícitas, declararam que “aprendem muito”.

A dúvida e a tentativa do fazer também são elementos disparadores e mobilizadores da aprendizagem. O talento artístico profissional é definido como a habilidade que o profissional possui de lidar com as zonas indeterminadas da prática. Respondendo às surpresas pela reformulação de estratégias, “ele comporta-se mais como um pesquisador tentando modelar um sistema especializado do que como um ‘especialista’ cujo comportamento é modelado”^(6)^. Por meio do ensino prático e reflexivo, os estudantes adquirem tipos de talento artístico essenciais para a competência em zonas indeterminadas da prática^(6)^.

Uma revisão integrativa, desenvolvida nos Estados Unidos, elencou as atividades mais frequentes encontradas na literatura que estimulam a prática reflexiva em cursos de graduação em enfermagem. A primeira mencionada foi as escritas reflexivas, nas quais os alunos constroem diários reflexivos críticos para avaliar o curso, refletir sobre os problemas durante estágios clínicos ou identificar pontos fortes e descobrir as necessidades de treinamento. Além disso, o estudo trouxe as discussões reflexivas como a segunda atividade que mais potencializa a reflexão. Segundo os pesquisadores, as discussões reduzem o estresse e a ansiedade dos aprendizes^(24)^.

Entende-se que o cotidiano da residência pode ser permeado por várias situações de dúvidas e incertezas, as quais devem ser tomadas como oportunidades de reflexão e proporcionar a aprendizagem. Para isso, é importante haver espaço para o residente expressar dúvidas e compartilhar com preceptores e tutores as vivências.

### Limitações do estudo

As limitações do estudo podem estar vinculadas à capacidade da pesquisadora de captar os elementos de reflexão expressos pelos residentes. Atentou-se a todos os acontecimentos, durante a coleta de dados, mas se admite que essa limitação é inerente ao método de observação. Deve-se ressaltar que, pela adesão e colaboração de todos os participantes do estudo, a pesquisadora conseguiu estar presente em todos os momentos de ensino-aprendizagem. Ademais, pode-se afirmar pela busca do rigor dessa investigação, por meio da triangulação, do trabalho de campo estendido e da lógica da replicação.

### Contribuições para a área da enfermagem, saúde ou política pública

A pesquisa contribui para o avanço do conhecimento na área de formação da residência em saúde, além de apontar adequações na organização e articulação do processo ensino-aprendizagem. Considera-se que o PRAPS é uma importante estratégia de formação para o SUS no Brasil.

## CONSIDERAÇÕES FINAIS

No cotidiano dos PRAPS, as atividades mais executadas pelos residentes foram aquelas em que menos expressaram elementos da prática reflexiva. Dessa forma, a reflexão do tipo conhecer-na-ação predominou no processo de ensino-aprendizagem de enfermeiros em programas de residência, especialmente durante as atividades práticas. Essa reflexão foi seguida pelas reflexões do tipo reflexão-na-ação e reflexão sobre a ação, com poucas oportunidades para os aprendizes expandirem a reflexão para a reflexão sobre a reflexão-na-ação.

A análise permitiu identificar que os elementos de reflexão “escutar com atenção operativa” e “expressar dúvidas” foram os mais expressos pelos residentes, e os próprios residentes, em autorreflexão, foram aqueles que mais potencializaram a reflexão. A prática de aprender fazendo e a discussão de casos clínicos foram consideradas estratégias potenciais para o processo de ensino-aprendizagem, destacando-se a dúvida e a tentativa do fazer como elementos disparadores e mobilizadores da aprendizagem. É necessário expandir os espaços, tempos e oportunidades para discutir as situações de dúvidas e incertezas que favoreçam a reflexão sobre a reflexão-na-ação, permitindo, assim, performances mais críticas dos residentes.

Situações de ensino-aprendizagem devem ser promovidas para intensificar as oportunidades de os alunos vivenciarem experiências diversas. Essa diversidade favorece o acúmulo de repertório de conhecimentos e experiências pelos residentes, preparando-os para lidar com as demandas impostas pela rotina de trabalho.

## Data Availability

https://doi.org/10.48331/scielodata.DXHQUL
